# Enhanced flight performance and adaptive evolution of Mesozoic giant cicadas

**DOI:** 10.1126/sciadv.adr2201

**Published:** 2024-10-25

**Authors:** Chunpeng Xu, Jun Chen, Florian T. Muijres, Yilun Yu, Edmund A. Jarzembowski, Haichun Zhang, Bo Wang

**Affiliations:** ^1^ State Key Laboratory of Palaeobiology and Stratigraphy, Nanjing Institute of Geology and Palaeontology, Chinese Academy of Sciences, Nanjing 210008, China.; ^2^ Institut für Zoologie und Evolutionsforschung, Friedrich-Schiller-Universität Jena, Jena 07743, Germany.; ^3^ Institute of Geology and Paleontology, Linyi University, Linyi 276000, China.; ^4^ Experimental Zoology Group, Wageningen University, Wageningen 6708 WD, Netherlands.; ^5^ Key Laboratory of Vertebrate Evolution and Human Origins, Institute of Vertebrate Paleontology and Paleoanthropology, Chinese Academy of Sciences, Beijing 100044, China.; ^6^ University of Chinese Academy of Sciences, Beijing 100049, China.; ^7^ Department of Earth Sciences, Natural History Museum, London SW7 5BD, UK.

## Abstract

Insects have evolved diverse ecological flight behaviors and adaptations that played a key role in their large-scale evolutionary patterns. However, the evolution of their flight performance is poorly understood because reconstructing flight abilities of extinct insects is highly challenging. Here, we propose an integrated approach to reveal the evolution of flight performance of Palaeontinidae (giant cicadas), a Mesozoic arboreal insect clade with large bodies and wings. Our analyses unveil a faunal turnover from early to late Palaeontinidae during the latest Jurassic–earliest Cretaceous, accompanied by a morphological adaptive shift and remarkable improvement in flight abilities including increased flight speed and enhanced maneuverability. The adaptive aerodynamic evolution of Palaeontinidae may have been stimulated by the rise of early birds, supporting the hypothesis of an aerial evolutionary arms race (Air Race) between Palaeontinidae and birds. Our results provide a potential example of predator-induced morphological and behavioral macroevolution and contribute to our understanding of how powered flight has shaped animal evolution.

## Teaser

The evolution of flight performance in Mesozoic giant cicadas suggests a potential aerial evolutionary arms race.

## INTRODUCTION

Powered flight is one of the most important behavioral innovations in animal locomotion and provides flyers several ecological advantages, such as long-distance migration and high-speed or great maneuverable motion unrealistic for nonflyers (*1*–*5*). Powered flight has evolved independently in four faunal lineages, insects, pterosaurs, birds, and bats (*2*, *3*). Insecta represents the earliest and most speciose flying group, displaying diverse wing morphologies, ranging from two-paired to one-paired, from folded to unfolded, and from membranous to elytral (*6*). These morphologies correspond to various ecological flight behaviors and adaptations, from long-distance migration to instant hovering, from flapping to gliding, and from predator avoidance to courtship behavior (*6*, *7*). The evolutionary history of insects has demonstrated their noticeable diversification in flight strategies since their origins as the first flying animals (*8*–*10*). Investigating the evolution of insect flight is therefore crucial to understanding how powered flight affects large-scale evolutionary patterns (*11*–*15*). Moreover, it may provide substantial information for elucidating how insects adjust their flight mechanisms to improve flight performance and aerial adaptation (*1*, *16*), which provides bioinspiration for the development of human-build aircraft such as micro air vehicles (*3*, *17*). However, reconstructing the flight performance and behavior of extinct insects has been proven to be highly challenging (*1*, *8*).

Predator-prey interactions between insects and other flying animals have been considered to be an important driving force of insects’ evolution, which bring about major changes in their morphology and behavior (*18*–*20*). For instance, the rise of bats affected the evolution of multiple morphological and behavioral traits in prey species, including the bat-induced macroevolution of insect hearing organs (*19*), insect wing shape (*11*), and insect flight performance (*18*). As ubiquitous flyers in extant terrestrial ecosystem, birds (Aves) and their insect prey constitute one of the most sophisticated predator-prey interactions known. Bird predation is a powerful selective force shaping many behavioral and morphological traits in insect prey, e.g., flight performance (*18*, *20*), body size (*21*), flight muscles (*22*), wing shape, and color pattern (*23*, *24*). In the Late Jurassic, paravians radiated and diversified, resulting in the appearance of birds capable of powered flight (*25*–*27*). Therefore, the rise of birds from the latest Jurassic might provide a natural experiment to test the importance of predation and decode predator-induced macroevolutionary trends. Although the origin and early evolution of birds have been in the spotlight since the finding of the classic “missing link” *Archaeopteryx* (*25*, *26*, *28*, *29*), little is known about the ecological influence of these early birds via predator-prey interactions.

**Fig. 1.**
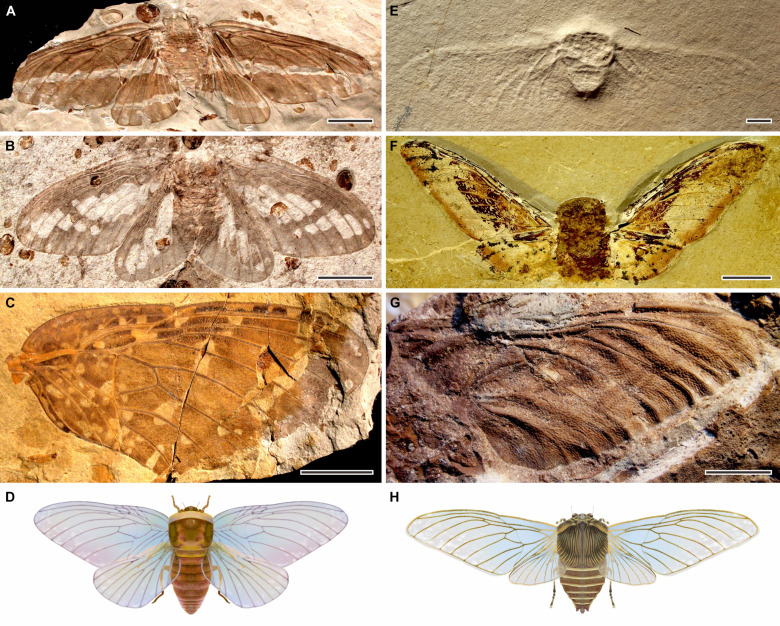
Representatives of Palaeontinidae. Early Palaeontinidae (**A** to **D**) and late Palaeontinidae (**E** to **H**). (A) *Palaeontinodes reshuitangensis*, from the Middle Jurassic Daohugou Konservat-Lagerstätte, China (NIGP156791). (B) *Sinopalaeocossus fangi*, from the Middle Jurassic Daohugou Konservat-Lagerstätte, China (NIGP150277). (C) *Martynovocossus punctulosus*, from the Middle Jurassic Daohugou Konservat-Lagerstätte, China (NIGP147878a). (D) Reconstruction of early Palaeontinidae based on *M. punctulosus*. (E) *Eocicada microcephala*, from the Upper Jurassic Limestone of Solnhofen, Germany (a well-preserved specimen deposited at the Museum Bergér). (F) *Baeocossus fortunatus*, from the Lower Cretaceous Crato Formation, Brazil (SMNS 65546). (G) *Ilerdocossus prowsei*, from the Lower Cretaceous Weald Clay Formation, England (BMB 014927). (H) Reconstruction of late Palaeontinidae based on *B. fortunatus*. Scale bars, 10 mm.

Palaeontinidae (Hemiptera: Cicadomorpha) is an extinct group of large arboreal insects ranging from the Middle Triassic to Late Cretaceous. They have an extremely long rostrum, even extending beyond the end of the abdomen, indicating that these insects were primarily xylem feeders, like modern cicadas (Cicadoidea) (*30*). Among the most diverse Mesozoic insects, they exquisitely preserve a vast array of morphological information on bodies and wings (Fig. 1 and figs. S1 to S4), thus representing an outstanding epitome of Mesozoic flying insects and an ideal model for investigating the relationship between wing morphology and flight behavior in the evolution. The Late Mesozoic macroevolutionary trends in wing shapes of Palaeontinidae have been noted by many researchers (*30*–*36*). However, the macroevolutionary trends have never been tested quantitatively because of the lack of systematic revision of some key fossils and thus resulting in the absence of a phylogeny for this group. Reconstructing the flight performance and behavior of ancient insects is notoriously difficult. Therefore, the relationship between wing morphology and flight abilities in palaeontinid evolution remains elusive.

In this study, we re-examined all representative Palaeontinidae and their closest relatives (Dunstaniidae) over a period of ~160 million years (Ma) and compiled a database of detailed morphometric information on their bodies and wings. Using this, we inferred the first phylogeny of Palaeontinidae and reconstructed their evolutionary history based on Bayesian tip dating, phylogenetic morphospace, morphological disparity, and geometric morphometric analyses. In addition, we developed an aerodynamic modeling approach to estimate the flight performance of Palaeontinidae on the basis of their wing and body characteristics and used this to analyze the evolution of their flight ability. Our results reveal an abrupt morphological transition from early Palaeontinidae to late Palaeontinidae during the latest Jurassic to earliest Cretaceous, leading to a distinct increase in flight performance. Our study provides evidence for the following two questions: (i) how to assess the evolution of flight abilities of extinct insects; (ii) how the rise of early birds might influence the evolution of flying insects.

## RESULTS

### Morphology and phylogeny of Palaeontinidae

We examined almost all representatives of Palaeontinidae and Dunstaniidae based mostly on direct specimen observation and occasionally on new high-resolution images (data S1). Palaeontinidae and Dunstaniidae share the same bauplan composed of a robust body with a small head and a large mesothorax, and a pair of membranous forewings and hind wings supported primarily by several longitudinal main veins (Fig. 1 and figs. S1 to S6). We further compiled a series of databases containing detailed morphological information on their wings (data S2 to S4). Subsequently, we reconstructed the first phylogeny of Palaeontinidae and their closest relatives (Dunstaniidae) based on the analysis of wing characters using maximum parsimony and Bayesian inference.

**Fig. 2.**
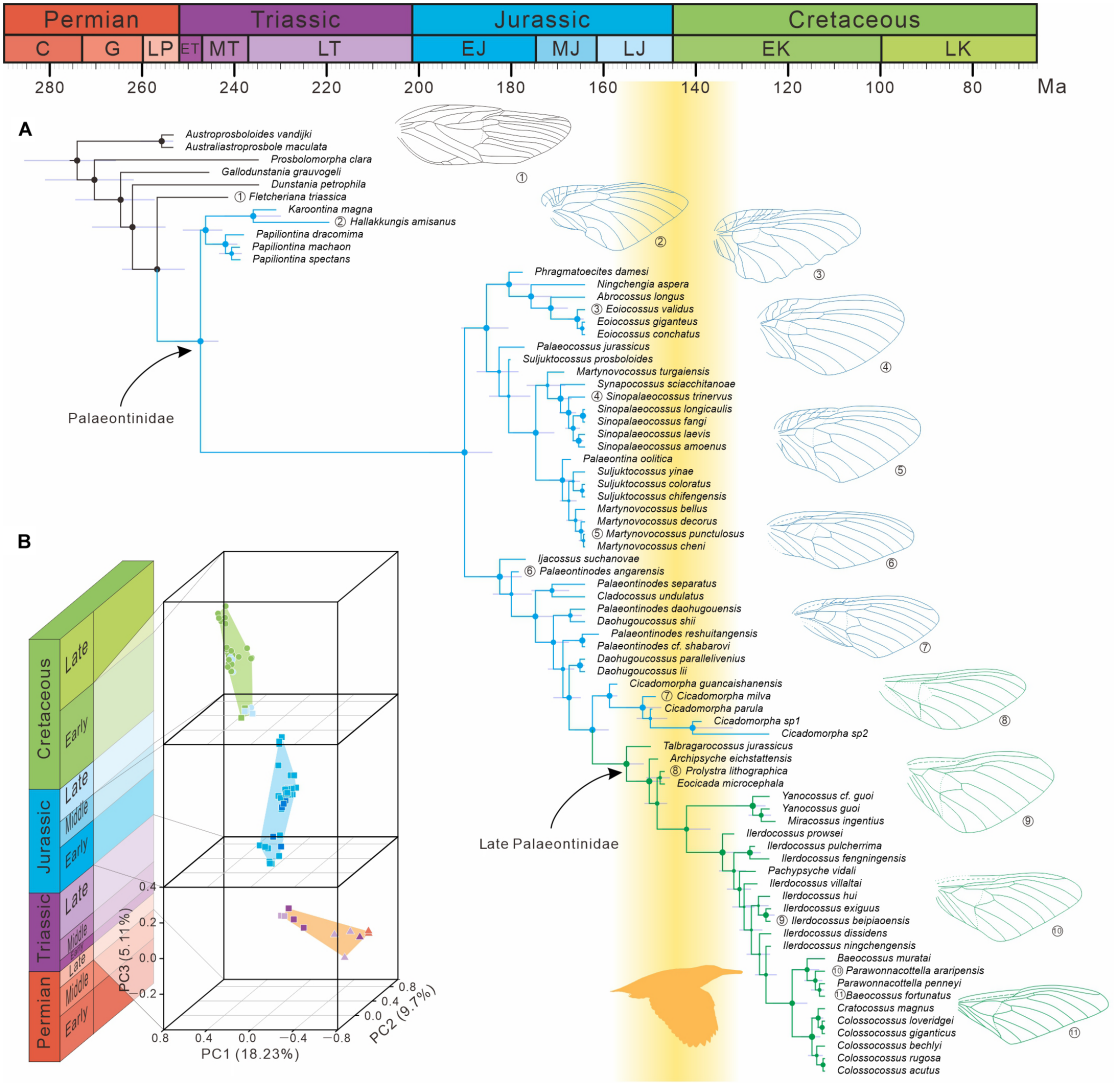
The evolutionary history of Palaeontinidae. (**A**) Simplified consensus tree using Bayesian phylogenetic tip-dating analysis. On the simplified consensus tree (50% majority rule), size of node spots indicates posterior probabilities to two significant digits. The error bars (in violet) at the internal nodes denote the 95% HPD intervals of age estimates. Black branches represent Dunstaniidae, blue branches represent early Palaeontinidae, and green branches represent late Palaeontinidae. Representatives of Dunstaniidae, early Palaeontinidae, and late Palaeontinidae are indicated in black, blue, and green line drawings, respectively. The yellow area represents the Air Race hypothesized as induced by the diversification of early birds from latest Jurassic to earliest Cretaceous. (**B**) Morphospace comparisons of Dunstaniidae and Palaeontinidae from the Late Permian to Early Cretaceous, divided into three periods: period 1, Late Permian–Late Triassic; period 2, Early-Middle Jurassic; period 3, Late Jurassic–Early Cretaceous. C, Cisuralian; G, Guadalupian; LP, Lopingian; ET, Early Triassic; MT, Middle Triassic; LT, Late Triassic; EJ, Early Jurassic; MJ, Middle Jurassic; LJ, Late Jurassic; EK, Early Cretaceous; LK; Late Cretaceous.

The general tree topologies based on three different morphological matrices (all characters and taxa; excluding taxa with only hind wing data; and excluding taxa with only hind wing data and hind wing characters) are largely consistent with a robust topology. Our results provide strong support for the monophyly of Palaeontinidae (Fig. 2A and figs. S7 to S13). The Dunstaniidae is obviously a paraphyletic group, with the genus *Fletcheriana* as the sister group of Palaeontinidae. In addition, our tip-dating tree provides the estimated divergence times for major clades of Palaeontinidae (Fig. 2A and fig. S13). Palaeontinidae are estimated to have originated near the beginning of the Middle Triassic [about 247.4 Ma; Highest Posterior Density (HPD), 252.9 to 243.5 Ma] and became extinct in the Late Cretaceous, with the last occurrence from mid-Cretaceous Kachin amber (fig. S4F).

### Late Mesozoic palaeontinid faunal turnover

**Fig. 3.**
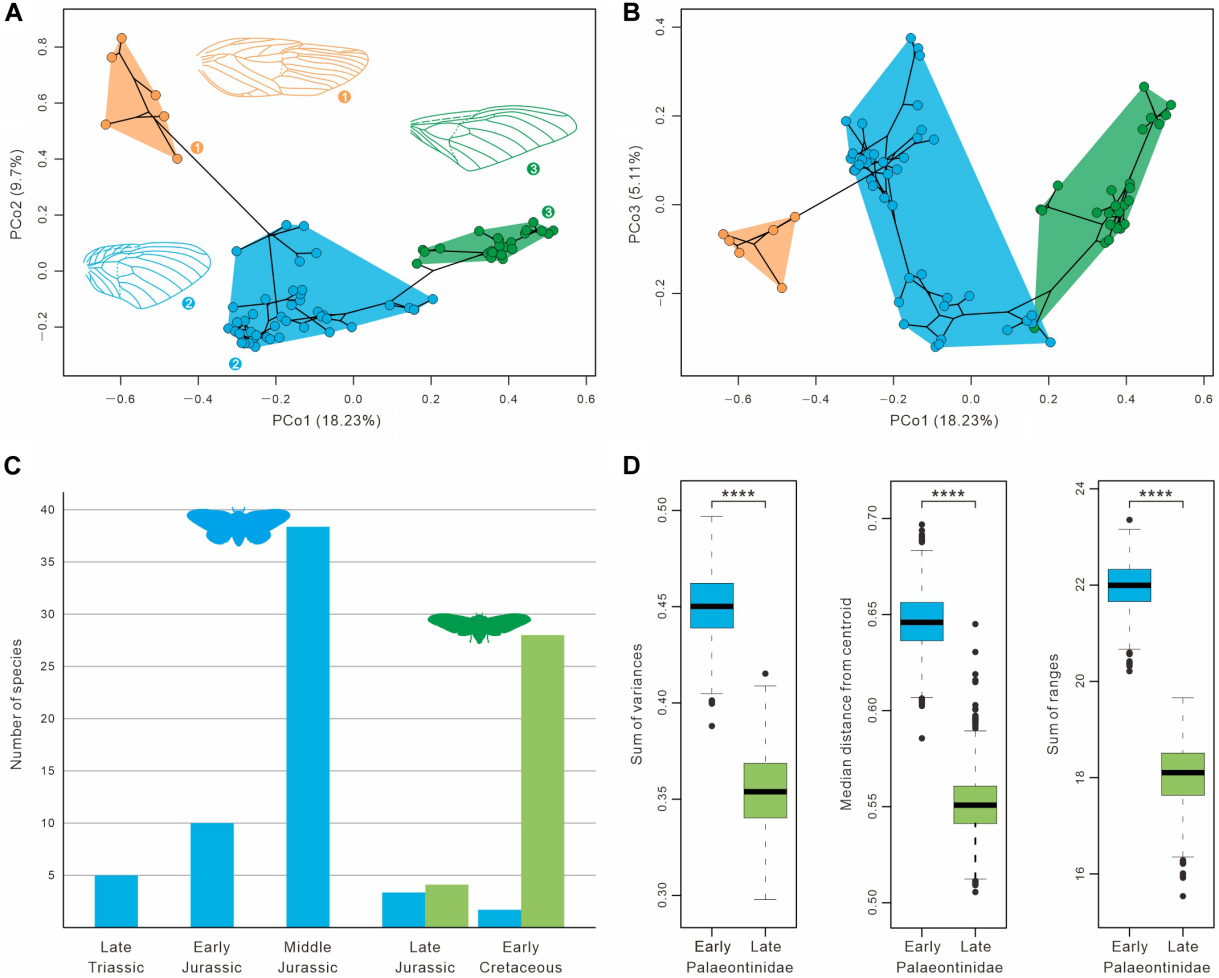
Morphological disparity of forewings of Palaeontinidae and Dunstaniidae. (**A** and **B**) The first three principal components (PCo1, PCo2, and PCo3) within the phylogenetic morphospace of forewings, partitioned among Dunstaniidae, early Palaeontinidae, and late Palaeontinidae (see also figs. S15 to S17). (**C**) Temporal dynamics of the diversity of Early and Late Mesozoic Palaeontinidae, from the Late Triassic to Early Cretaceous. (**D**) Comparison of morphological disparity among early and late Palaeontinidae (the boxes represent the median and the first and third quartiles of morphological disparity; *n* = 76 species). Morphological disparity was compared using a Welch’s *t* test for statistical significance (two-sided ****P value < 0.001). In all panels, orange data are of Dunstaniidae, blue data represent early Palaeontinidae, and green data represent late Palaeontinidae.

During its long evolutionary history from the Triassic to the Cretaceous, Palaeontinidae reached its pinnacle in taxonomic diversity in the Middle Jurassic, slightly decreased in the Late Jurassic but recovered in the Early Cretaceous (Fig. 3C). The Palaeontinidae was previously divided broadly into two groupings: early Palaeontinidae with elliptical or subtriangular forewings and large hind wings and late Palaeontinidae with triangular forewings and comparatively small hind wings (*30*, *36*). Early Palaeontinidae are clearly a paraphyletic group comprising all Late Triassic–Middle Jurassic palaeontinids (Late Triassic, 5 species; Early Jurassic, 10 species; Middle Jurassic, 38 species) and 5 Late Jurassic–Early Cretaceous species (all belonging to *Cicadomorpha*; Late Jurassic, 3 species; Early Cretaceous, 2 species). Our phylogenetic analysis supports the monophyly of the late Palaeontinidae and shows that the genus *Cicadomorpha* is its sister group (Fig. 2A and fig. S13). Late Palaeontinidae are distinguished from most early Palaeontinidae by forewings with the reduced costal area and clavus and much smaller hind wings (Fig. 2A and figs. S7 to S9). They probably originated near the beginning of the Late Jurassic (Fig. 2A and fig. S13) and became widespread during the latest Jurassic (one genus and species in Australia and three genera and species in Germany). In the Early Cretaceous, they are quite abundant with an extremely high diversity (more than 26 species). Thus, a distinct palaeontinid transition from early Palaeontinidae– to late Palaeontinidae–dominated fauna occurred during the latest Jurassic–earliest Cretaceous (Figs. 2A and 3C and fig. S13).

### Forewing evolution of Palaeontinidae

The flight of Palaeontinidae and Dunstaniidae is anteromotoric, being driven primarily by the forewings, with hind wings coupled mechanically to the forewings and flapping in synchrony with them. The characters of the forewings, such as shape, venational pattern, and wing cells, are involved in controlling the extent of wing deformation and bending during flight. Therefore, their forewings provide the most important clues for decoding the flight capacity.

To precisely quantify the evolutionary history of forewing gross morphology (shape, venational pattern, wing cells, and other structures), we carried out phylogenetic morphospace and morphological disparity analyses using all forewing characteristics (data S5). Dunstaniidae and Palaeontinidae together exhibit a distinct migration in morphospace through their whole evolutionary trajectory, and their disparity reached a climax during the Early-Middle Jurassic (Fig. 2B, fig. S14, and movie S1). Our phylogenetic morphospace analyses further show that a distinctive morphospace partition occurs between Dunstaniidae, early Palaeontinidae and late Palaeontinidae, and the late Palaeontinidae occupied a smaller morphospace than that of early Palaeontinidae (Fig. 3, A and B, and figs. S15 to S17). Our permutational multivariate analyses of variance (PERMANOVA) tests also demonstrate a statistical separation between the early and late Palaeontinidae in morphospace (table S1). These results are robust in both principal coordinates analysis (PCoA) and nonmetric multidimensional scaling (NMDS) analysis (figs. S15 to S17 and table S2). In addition, our analyses of forewing morphological disparity show that early and late Palaeontinidae are statistically distinct from each other in all disparity metrics [*P* value < 0.001 in sum of variances (SOV), median distance from centroid (MDC), and sum of ranges (SOR); table S3]. Also, early Palaeontinidae exhibit greater disparity than late Palaeontinidae in all disparity metrics (Fig. 3D and fig. S18). Rarefaction analyses show that these results are not significantly affected by sampling bias (fig. S19).

**Fig. 4.**
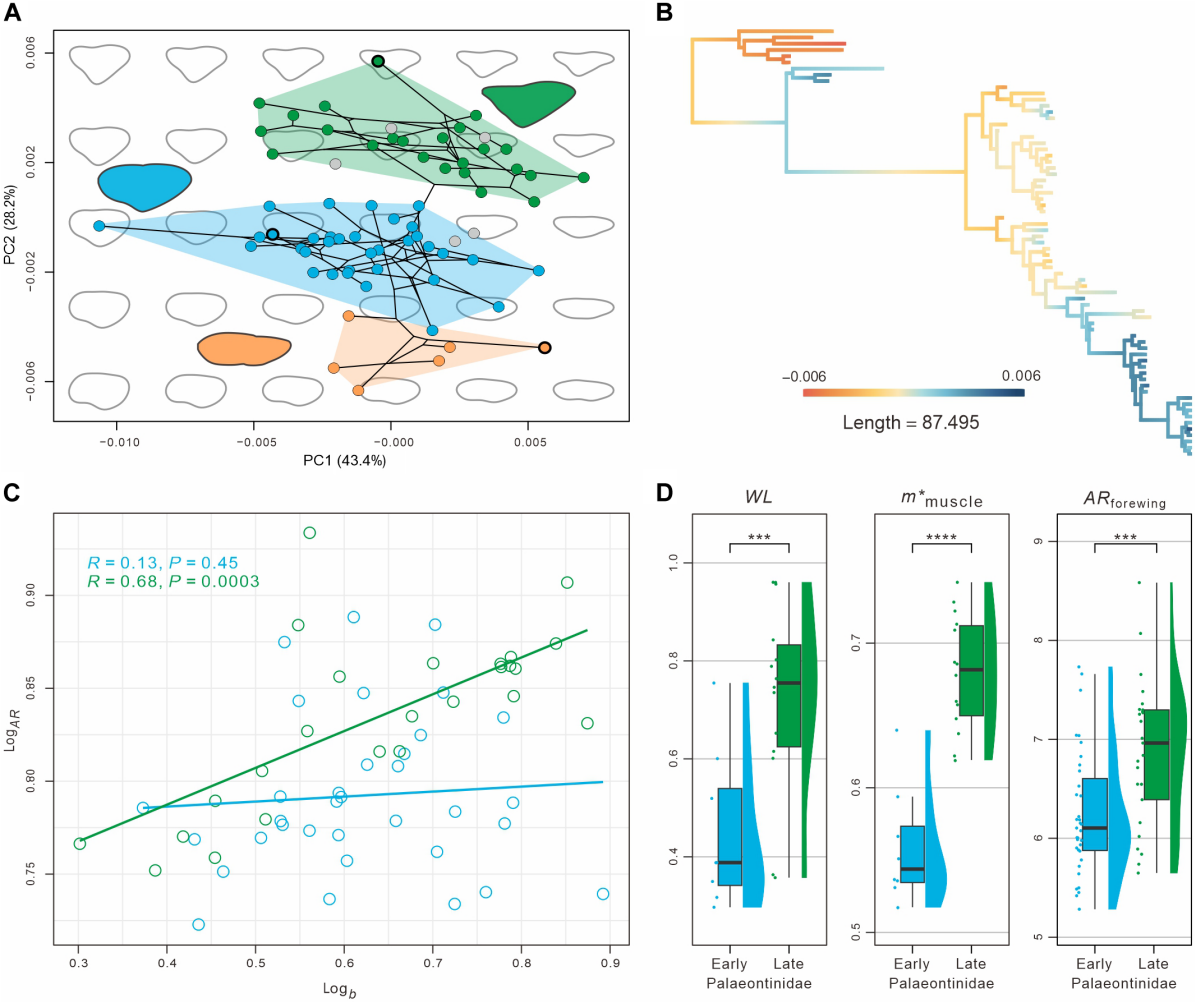
Geometric morphometric analysis of forewing shapes and flight performance traits of Palaeontinidae. (**A**) The first and second principal components (PC1 and PC2, respectively) of the forewing shape morphospace, partitioned among Dunstaniidae, early Palaeontinidae, and late Palaeontinidae. (**B**) Changes in PC2 across the time-calibrated phylogeny. (**C**) Allometric relationships between wingspan *b* and forewing aspect ratio *AR*_forewing_ of early and late Palaeontinidae species (in blue and green, respectively). Regression slopes differ significantly between early and late Palaeontinidae (ANCOVA, *P* value < 0.001). (**D**) Comparison of flight performance traits among early and late Palaeontinidae, including wing loading *WL*, the muscle mass normalized with body mass m*_muscle_, and forewing aspect ratio *AR*_forewing_. Each dataset is illustrated by raincloud (individual data points, a half violin plot, and a box plot with the median and the first and third quartiles of values). Flight traits were compared using the two-sided Welch’s *t* test for statistical significance (****P value < 0.001, ***P value < 0.01). Orange data are of Dunstaniidae, blue data represent early Palaeontinidae, and green data represent late Palaeontinidae.

Wing shape evolves optimizing flight performance under a balance of costs and benefits, and thus, it is closely related to the flight performance. To further investigate the evolution of forewing shape in detail, we assembled an unscaled forewing outline dataset of Palaeontinidae and Dunstaniidae and performed a geometric morphometric analysis via principal components analyses (PCA). Our analyses of forewing shape disparity show that early Palaeontinidae are more disparate than late Palaeontinidae (*P* value < 0.001 in SOV, MDC, and SOR; fig. S20 and table S4). The scatterplot of the first two principal component axes (PC1 and PC2) shows that early and late Palaeontinidae each have clustered wing shapes in morphospace, with a distinctive partition (Fig. 4A, fig. S21, and tables S5 and S6). Early Palaeontinidae are concentrated in a region of morphospace that is distributed across PC1 and in the median of PC2. On the other hand, most late Palaeontinidae are concentrated in a region of morphospace that is distributed across PC1 and in the upper half of PC2. PC1 approximately corresponds to the length/width ratio from low to high, and PC2 describes the contour features of forewings from suboval to subtriangular. Differences along PC2 distinguish late Palaeontinidae from early Palaeontinidae, and mapped changes of PC2 across the phylogeny exhibit a clear trend of forewing triangularization along the palaeontinid lineage (Fig. 4, A and B, and figs. S22 and S23).

The early Palaeontinidae have higher ratios of areal proportion of the costal area to the forewing (S*_costal_, median values are 0.081 in early Palaeontinidae and 0.054 in late Palaeontinidae), and areal proportion of the clavus to the forewing (S*_clavus_, median values are 0.085 in early Palaeontinidae and 0.049 in late Palaeontinidae) than those of late Palaeontinidae (Welch’s *t* test, *P* value < 0.001; figs. S5, S6, and S24 and table S7). The costal area and clavus are situated at the base of the forewing, and thus, their reduction causes the posterior margin to become more acute and the forewing shape to become more triquetrous (Fig. 2 and figs. S5 and S6). The triangularization of forewings along the palaeontinid lineage is mainly caused by the reduction of the costal area and clavus.

### Flight performance of Palaeontinidae

We hypothesize that late Palaeontinidae have evolved improved flight abilities, as a result of the aerial evolutionary arms race between early predatory birds and Palaeontinidae as prey. The primary flight performance metrics under evolutionary pressure in such predator-prey arms race are locomotory speed, efficiency, and maneuverability (*12*, *16*). We therefore developed a set of simplified qualitative flight performance models to estimate flight performance on the basis of three morphology-derived metrics (fig. S25; see Materials and Methods): (i) Flight speed scales with the square root of wing loading [*U* ~ √(*WL*)] (*7*), and thus, we used wing loading as an estimate for the flight speed of flying Palaeontinidae; (ii) the power required for both high-speed flight and rapid flight maneuvers is produced by the flight power muscles, where available power scales linearly with flight muscle mass (*P*_avail_ ~ *m*_muscle_) (*37*). We therefore estimated body mass-normalized power availability (P*_avail_ ~ *P*_avail_/*m*_body_) on the basis of the equivalent normalized flight muscle mass, as m*_muscle_ = *m*_muscle_/*m*_body_; (iii) maximum flight speed scales also with flight efficiency, as this directly affects the power required for high-speed flight; this required power scales inversely with the aspect ratio of the wing (*P*_req_ ~ 1/*AR*) (*7*). Therefore, we quantified flight efficiency using the wing aspect ratio of both the forewings and the forewings and hind wings combined (*AR*_forewing_ and *AR*_combined_, respectively).

We determined the wing morphology parameters on forewings of 65 species and combined wings of 21 species; in addition, the body morphology parameters were estimated on the bodies of 22 species (data S6 to S9; see Materials and Methods). Comparing the resulting flight performance parameters between early and late Palaeontinidae shows that all three metrics are significantly higher in the late Palaeontinidae (Fig. 4D, fig. S26, and table S8). This suggests that late Palaeontinidae have superior flight performance compared to early Palaeontinidae.

The median wing loading of late Palaeontinidae is 92% higher than that of early Palaeontinidae [early Palaeontinidae: *WL* = 0.39 (0.30 to 0.75); late Palaeontinidae: *WL* = 0.75 (0.36 to 0.96); Welch’s *t* test, *P* value < 0.005], which would approximately lead to a 39% increase in flight speed [*U* ~ √(*WL*)]. Moreover, the median relative flight muscle mass of late Palaeontinidae is 19% higher than that of early Palaeontinidae [early Palaeontinidae: m*_muscle_ = 0.54 (0.52 to 0.64); late Palaeontinidae: m*_muscle_ = 0.68 (0.62 to 0.74); Welch’s *t* test, *P* value < 0.001], suggesting a similar increase in relative flight power availability (P*_avail_ ~ m*_muscle_). The median wing aspect ratio of the forewings and combined wings is 15% and 26% higher in late Palaeontinidae than in early Palaeontinidae, respectively [early Palaeontinidae: *AR*_forewing_ = 6.10 (5.28 to 7.73), *AR*_combined_ = 3.4 (2.8 to 4.0); late Palaeontinidae: *AR*_forewing_ = 7.0 (5.7 to 8.6), *AR*_combined_ = 4.3 (4.0 to 5.5); Welch’s *t* test, *p*_forewings_ < 0.005, *p*_combined_ < 0.001]. This suggests that late Palaeontinidae were more efficient flyers, allowing them to reach higher flight speeds and perform more rapid maneuvers with a given flight power availability.

In addition, to test whether a correlation existed between size and flight abilities in Palaeontinidae, we correlated forewing aspect ratio *AR*_forewing_ with wingspan *b* (Fig. 4C). The Pearson test was used to further explore the *AR*_forewing_-*b* relationship in early and late Palaeontinidae, respectively. The results show that the aspect ratio is significantly positively correlated with wingspan in the late Palaeontinidae (*P* value < 0.001, Cor.coeff = 0.68), but it is not correlated with wingspan in the early Palaeontinidae (*P* value = 0.45) (Fig. 4C). Our analysis of covariance (ANCOVA) shows that the *AR*_forewing_-*b* allometric slopes differ significantly between early and late Palaeontinidae (ANCOVA, *P* value < 0.05; fig. S27). Thus, for early Palaeontinidae, wing aspect ratio does not vary with size, while for late Palaeontinidae, it does, suggesting increased flight efficiency in large species of late Palaeontinidae compared to smaller ones.

## DISCUSSION

Our results reveal a latest Jurassic–earliest Cretaceous transition from an early Palaeontinidae– to a late Palaeontinidae–dominated fauna with regards to the phylogeny, taxonomic diversity, and phenotypic evolution. After its appearance in the Late Jurassic, late Palaeontinidae diversified rapidly and dominated the palaeontinid fauna in the Early Cretaceous (Figs. 2A and 3C and fig. S13). Our phylogenetic morphospace and morphological disparity analyses show that late Palaeontinidae occupied a smaller morphospace in terms of forewing shapes and gross morphology (Fig. 3 and figs. S15 to S19). There is a distinct forewing shape transition from the oval condition in early Palaeontinidae to the triangularization of late Palaeontinidae, which mainly resulted from the reduction of the costal area and clavus (Fig. 4A and fig. S24). In addition, late Palaeontinidae have a significantly higher wing loading (*WL*), relative flight power muscle mass (m*_muscle_), and wing aspect ratio (*AR*) than early Palaeontinidae (Fig. 4D and fig. S26).

Combining these results with our insect flight model demonstrates that this adaptive shift in morphology of the flight motor system underlies an improvement of flight abilities among palaeontinids, corresponding to their latest Jurassic–earliest Cretaceous faunal turnover. It is noteworthy that Palaeontinidae and Cicadoidea (modern cicadas), both belonging to the clade Cicadomorpha, have similar wing structures including the wing-coupling apparatus and the nodal line (*38*). In particular, the nodal line, a transverse flexion line that crosses the main longitudinal veins in the forewing and separates the basal supporting and distal deformable areas, is a highly specialized structural adaptation for ventral transverse bending occurring at the bottom of the downstroke or during the upstroke (*39*). It further suggests that Palaeontinidae and modern cicadas have adopted the same flight mechanism (*31*). Over the past couple of years, there has been tremendous progress in understanding the flight system of insects such as in living cicadas (*40*–*42*), which augment our recognition of wing functional morphology in fossil insects. Wing morphometric variables are essential to flight and are universally acknowledged to have aerodynamic consequences for flying animals, including flight speed and maneuverability (*7*). Wing loading (*WL*), relative flight power muscle mass (m*_muscle_), and wing aspect ratio (*AR*) are widely acknowledged as three indicative parameters of flight ability among insects, including cicadas (*12*, *13*, *16*, *43*, *44*).

Wing loading is a body-wing interactive parameter that is positively correlated to flight speed in both insects and vertebrates, as flight speed *U* scales with the square root of wing loading [*U* ~ √(*WL*)] (*12*, *45*–*47*). The observed 92% higher wing loading thus indicates a 39% faster flight speed for late Palaeontinidae compared to early Palaeontinidae (Fig. 4D). In addition, higher wing loading is frequently associated with a greater proportion of flight power muscle mass, which results in a higher relative power availability for flight (*12*, *48*). Late Palaeontinidae correspondingly evolved a larger mesothoracic volume than early Palaeontinidae, resulting in a 19% higher relative flight muscle mass (m*_muscle_). This increase in mesothoracic flight muscle mass provides late Palaeontinidae with higher flight power availability, enabling them to fly faster and maneuver more rapidly (Fig. 4D). Aspect ratio is a wing morphometric parameter that is positively correlated with flight efficiency (see Materials and Methods) (*48*). Having long, slender wings with a higher aspect ratio reduces the power requirements of flight through a reduction of wing-tip vortex circulation and consequently an increase in lift-to-drag ratio (*12*, *16*, *45*, *49*, *50*). The higher–aspect ratio wings of late Palaeontinidae demonstrate that they were more efficient flyers than early Palaeontinidae (Fig. 4D and fig. S26), and the same phenomenon was also observed in cicadas (*51*). Our allometric analyses further suggest that larger species of late Palaeontinidae required more energy-saving flight (Fig. 4C and fig. S27). Enhanced flight efficiency does not only reduce energetic cost of transport (*52*), but it also reduces the power requirement for high-speed flight and rapid evasive maneuvers.

The combination of high wing loading, high relative flight muscle mass, and high aspect ratio of wings is a typical pattern of high-speed flying insects (*12*, *53*), confirming the high-speed flight of late Palaeontinidae. Moreover, the high relative flight muscle mass and high–aspect ratio wings of late Palaeontinidae results in high power availability and low power requirement for flight, respectively. This not only enables late Palaeontinidae to achieve high flight speeds but also allows them to carry out rapid evasive maneuvers that are often power limited. In conclusion, compared to early Palaeontinidae, late Palaeontinidae had evolved a remarkably more advanced flight capability, including higher flight speed and greater maneuverability.

In addition, the forewing triangularization along the palaeontinid lineage suggests an evolutionary trend for increased flight abilities. Dunstaniidae, such as species of the genus *Fletcheriana*, have a forewing with a wide costal area supported by veins R (radius), CP (costa posterior), and ScP (subcosta posterior) veinlets (Fig. 2A and fig. S1, B to E). In contrast, Palaeontinidae have a narrow costal area in the forewing, while late Palaeontinidae have an even narrower costal area and vein ScP fused with vein R basally (Fig. 1 and figs. S5 and S6). In forewings of extant cicadas, veins ScP and RA (radius anterior) are coalesced as the thickest vein, to strengthen the leading edge of the forewing, and this fused vein is located close to the costal margin to form a very narrow costal area (*54*). In forewings of Palaeontinidae and modern cicadas, the main longitudinal veins gradually become thinner toward the distal end (Fig. 1 and figs. S1 to S4) (*54*). On the leading edge of the forewing, the longitudinal veins form a rigid span supporting the wing as it moves through the air (*6*). Insect flight relies heavily on the production of unsteady leading-edge vortices that reduce in strength along the span toward the wingtip (*55*, *56*). For species of living cicada with forewings of the same size, those with rigid leading edges can generate larger lift coefficients than those with flexible leading edges (*44*). Therefore, the reduced costal and rigid leading edge, as present in the forewing of late Palaeontinidae, suggest effective wing support and increased lift production capability. In addition, the reduced clavus in late Palaeontinidae indicates an evolutionary trend toward smaller hind wings. The forewing clavus is locked with the hind wing by the wing-coupling fold and lobe, and therefore, its size is related to that of the hind wing. Triassic Dunstaniidae and early Palaeontinidae retain large hind wings, with forewing lengths less than or equal to 1.8 times the hind wing (*57*). However, late Palaeontinidae have smaller hind wings with the ratio of forewing to hind wing length usually over 1.8 (Fig. 1 and figs. S3 and S4). Because of the decrease of the hind wing size of late Palaeontinidae, their forewing and hind wing jointly form a distinct triangular shape when coupled together (Fig. 1 and fig. S3), further suggesting more agile flight over a wide speed range (*31*). In conclusion, our comparative morphological analyses provide additional support for the superior flight abilities of late Palaeontinidae.

**Fig. 5.**
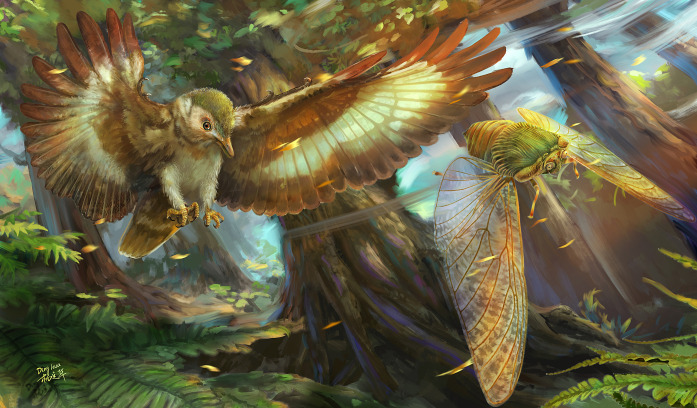
Ecological restoration of predator-prey interaction (chasing flights) between early birds (*Longipteryx chaoyangensis*) and late Palaeontinidae (*B. fortunatus*) in the Early Cretaceous. Artistic reconstruction by D. Yang.

Our results support the hypothesis of an aerial evolutionary predator-prey arms race between birds (predators) and Palaeontinidae (prey) (Fig. 2) (*36*), which from here on we refer to as an evolutionary “Air Race.” From the Late Jurassic to Early Cretaceous, paravians underwent sustained diversification including the origination of birds, with the acquirement of powered flight largely ascribed to limb and feather evolution (*25*, *26*). Early birds (stem group of birds) emerged as dominant volant predators in the forest ecosystem, and most of them probably fed regularly, or even exclusively, on insects (*25*, *58*). Palaeontinidae were notable flyers in arboreal habitats, and their large bodies must have made them an easy target for predators and a prior food source for early insectivorous birds. Like extant cicadas (*43*, *51*), Palaeontinidae could evade aerial predation by fast and agile flight when chased by birds. From the Late Jurassic, early Palaeontinidae distinctly declined while late Palaeontinidae emerged and spread rapidly throughout the Pangean supercontinent (Figs. 2A and 3C). Coinciding with the origination and diversification of early birds during the latest Jurassic–earliest Cretaceous, the palaeontinid faunal turnover may have been influenced by the powered flight achievement of early insectivorous birds, which likely imposed strong selective stress on Palaeontinidae (Fig. 2). Large flying insects such as Palaeontinidae, an almost untapped source of animal protein, may have been a stimulus for the evolution of early birds’ arboreal and aerial habitats, as well as their intricate flight systems. Conversely, early birds with progressive flight abilities could have exerted selective pressure on the evolution of Palaeontinidae. Thus, the Air Race between early birds and Palaeontinidae might have had reciprocal effects and led to progressive evolutionary transformations during the Late Mesozoic, partly shaping the complex blueprint of bird and palaeontinid evolution (Fig. 5).

The rise of birds in the Late Mesozoic has probably influenced the evolution of two pre-existing flyers–pterosaurs and insects, arousing a remarkable evolutionary Air Race event during the latest Jurassic–earliest Cretaceous (Fig. 2). This proposed event is coeval with the well-known pterosaur faunal turnover from basal to pterodactyloid pterosaurs, accompanied by the increase of body size, migration to water-related habitats, as well as dietary habits from insectivorous dominated to multifarious (*59*–*61*). The competition between birds and pterosaurs is probably the main reason for pterosaur faunal turnover (*25*, *59*, *61*, *62*).

In addition, the rise of birds inevitably imposed greater predation pressure on insects. For instance, the reduction in both the maximum size of insects and the size of endophytic insect eggs, roughly coincident with the Air Race event, is thought to have resulted from the selective predation pressure by birds on larger insects (*21*, *63*). The Air Race event also overlapped with a turnover in the Odonata (dragonfly and damselfly) fauna from being dominated by Anisozygoptera to being dominated by Anisoptera and Zygoptera (*64*). At the same time, Odonata probably evolved more species with color patterns (*65*), and dragonflies evolved time-saving exophytic oviposition with a reduced ovipositor (*66*). In addition to Palaeontinidae and Odonata, other insects may have developed a variety of defensive adaptations, both morphological and behavioral, in response to the sudden increase in bird predation pressure. More detailed paleoecological studies of early bird-insect associations can provide a more comprehensive view of the Air Race event posited herein. In conclusion, the Air Race event may well have influenced the evolution of pterosaurs and insects, thus reshaping the aerial ecosystem.

## MATERIALS AND METHODS

### Materials

To investigate the phylogenetic, morphospace, and macroevolutionary history of Dunstaniidae and Palaeontinidae, we compiled an updated database of Palaeontinoidea (Mesogereonidae excluded; data S1). The new database includes all well-preserved taxa (Dunstaniidae 6 species of 6 genera; Palaeontinidae 88 species of 38 genera). We examined most Dunstaniidae (67%, 4 of 6 genera; 45%, 4 of 6 species) and Palaeontinidae (87%, 33 of 38 genera; 83%, 73 of 88 species) on the basis of mostly direct specimen observation and occasionally new high-resolution photos. Dunstaniidae include one genus and one species from the Middle Triassic of France (fig. S1A), one genus and one species from the Middle-Late Triassic of Australia (fig. S1B), and two genera and two species from the Late Triassic of South Africa (fig. S1, C and D). Early Palaeontinidae include 1 genus and 1 species from the Late Triassic of South Korea (fig. S1E), 1 genus and 1 species from the Early Jurassic of China (Xinjiang), 1 genus and 1 species from the Middle Jurassic of China (Gansu), 4 genera and 4 species from the Early Jurassic of Russia, 4 genera and 5 species from the Early Jurassic of Kyrgyzstan, 1 genus and 1 species from the Early to Middle Jurassic of Kazakhstan, 15 genera and 33 species from the Middle Jurassic Daohugou Konservat-Lagerstätte of China (Fig. 1, A to C, and fig. S2, A to H), 1 genus and 1 species from the Middle Jurassic of China (Reshuitang, Inner Mongolia), 1 genus and 1 species from the Middle Jurassic of China (Hebei), 1 genus and 1 species from the Late Jurassic of China, 1 genus and 1 species from the Early Cretaceous of Russia (fig. S2I), and 1 genus and 1 species from the Early Cretaceous Yixian Formation of China (fig. S2J). Late Palaeontinidae include three genera and 3 species from the Late Jurassic Solnhofen Konservat-Lagerstätte of Germany (Fig. 1E and fig. S3A), one genus and 1 species from the Late Jurassic Purlawaugh Formation of Australia (fig. S3B), three genera and 5 species from the Early Cretaceous Crato Formation of Brazil (Fig. 1F and figs. S3, C and E, and S4, A to D), three genera and 10 species from the Early Cretaceous Yixian Formation of China (fig. S3, D and F), two genera and 3 species from the Early Cretaceous Weald Clay Formation of England (Fig. 1G and fig. S3G), one genus and 1 species from the Early Cretaceous La Pedrera de Rúbies Formation of Spain (fig. S4E), and one unnamed genus and species from mid-Cretaceous Kachin amber from Myanmar (fig. S4F). Photographs were taken using a Sony α7 camera and a Zeiss SteREO Discovery V20 microscope system with specimens moistened in 95% alcohol or dry. The figures were prepared with CorelDraw 2021 and Adobe Photoshop CS6.

All specimens are from Australian Museum, Sydney, Australia (prefix AMF); Bernard Price Institute, South Africa (prefix C-Dt.); Booth Museum of Natural History, Brighton, UK (prefix BMB); Capital Normal University, China (prefix CNU); Department of Earth Science Education, Kongju National University, Korea (prefix KNU); Institut de Géologie, Université Louis Pasteur, Strasbourg, France (*Gallodunstania grauvogeli* holotype); Jura Museum Eichstaett (prefix JME); Maidstone Museum & Bentlif Art Gallery, UK (prefix MNEMG); Museum Bergér, Germany; Museum of Natural History, Vienna, Austria (prefix NHMW); Nanjing Institute of Geology and Palaeontology, Chinese Academy of Sciences (prefix NIGP); Natural History Museum, London (prefix NHMUK); National Museum, Prague, Czech (prefix NM); Palaeontological Institute, Russian Academy of Sciences (prefix PIN); Senckenberg Naturhistorische Sammlungen Dresden, Germany (prefix BaJ); Shandong Tianyu Museum of Nature, China (prefix STMN), and Staatliches Museum for Naturkunde Stuttgart, Germany (prefix SMNS).

### Phylogenetic analyses

We developed a comprehensive phylogenetic framework for the Mesozoic Dunstaniidae and Palaeontinidae. The family Mesogereonidae, which is tentatively attributed to Palaeontinoidea, was not included in our analysis because of its controversial superfamilial assignment. We conducted phylogenetic analyses using maximum parsimony and Bayesian inference optimality criteria and models. The 50% majority-rule consensus trees are displayed in figs. S7 to S9, and the Bayesian nonclock trees are displayed in figs. S10 to S12.

Our study used a total of 89 operational taxonomic units: 6 species of Dunstaniidae and 83 species of Palaeontinidae. The selection of taxa contains all definite dunstaniid and palaeontinid species preserved with sufficient morphological data, ranging from the Late Permian to Early Cretaceous. Data matrices were constructed using characters from forewings and hind wings, while the morphology of the body was excluded because of the rarity of preservation (data S1). To investigate the phylogeny of Dunstaniidae and Palaeontinidae more precisely, we compiled three data matrices: Phylogenetic matrix 1 includes 119 characters of 92 species (all characters and species; data S2), phylogenetic matrix 2 includes 119 characters of 76 species (all characters; species with only hind wing information are excluded; data S3), and phylogenetic matrix 3 includes 77 characters of 76 species (characters of hind wings are excluded; species with only hind wing information are also excluded; data S4). *Austroprosboloides vandijki* (or *A. vandijki* and *Austroprosbole maculata*) was placed as the ultimate outgroup herein. Phylogenetic analyses were conducted using parsimony-based analyses PAUP*, version 4.0a169 (*67*) and likelihood-based Bayesian estimation MrBayes 3.2.7a (*68*).

For maximum parsimony analyses, the heuristic search was conducted with the following settings: All characters were unordered and given equal weight; the starting tree(s) was obtained via stepwise addition; the addition sequence was random with the starting seed generated automatically; the number of replicates was 100; tree-bisection-reconnection was used and set up with reconnection limit equal to eight; the steepest descent option was not in effect; the MulTrees option was not in effect, and one tree was saved per replicate; no topological constraint was in effect; trees were unrooted. Nonparametric bootstrap analyses with 1000 replicates were performed to assess nodal reliabilities for the resulting topologies. Three relevant logs of PAUP analyses are provided in the Supplementary Materials.

For Bayesian analyses, we first inferred the phylogeny from the morphological data only (nonclock analyses). The Lewis Mkv model with gamma rate variation across characters (Mkv + Γ) was used for the morphological characters. The Mkv + Γ model has only one free parameter (the gamma shape), which was assigned an exponential (1.0) prior by default. We used the uniform prior for the topology and gamma-Dirichlet prior for the branch lengths. We executed two independent runs and four chains per run (one cold chain and three hot chains with temperature 0.06) in the Markov chain Monte Carlo. Each run was executed for 20 million generations and sampled every 2000 generations. The first 25% of samples were discarded as burn-in, and the remaining samples from the two runs were combined. The posterior trees were summarized as a 50% majority-rule consensus tree, displaying all branches. We also performed a tip-dating analysis in which the fossil ages were incorporated as tip calibrations so that the divergence times and evolutionary rates in the tree can be coestimated. The chronological constraints for the taxa used in our analyses are erected from the PBDB (The Paleobiology Database) (data S1). In the tip-dating analyses, the prior for the time tree was modeled by the fossilized birth-death process. The process was conditioned on the time of the most recent common ancestor (root age) and had hyperparameters of speciation rate, extinction rate, fossil-sampling rate, and extant-sampling probability. The ages of the fossil taxa were given uniform distributions on the basis of their corresponding stratigraphic ranges. For analysis purpose, the age of the youngest taxa (*Parawonnacottella*, *Cratocossus*, *Colossocossus*, and *Baeocossus*) were set to zero, with all other ages rescaled accordingly. The extant-sampling probability was set as random sampling. The fossil sampling was set with three rate shifts at 157(45), 174(62), and 208(96) Ma at the middle of the Oxfordian, end of Toarcian, and Norian. To root the tree properly and infer the evolutionary rates more reliably, we applied three topology constraints: Triassic palaeontinids, early Palaeontinidae, and late Palaeontinidae. The same Markov chain Monte Carlo settings were used in the nonclock analyses. The nonclock trees are presented in figs. S10 to S12, and the tip-dating tree is presented in Fig. 2 and fig. S13. The trees were drawn using FigTree v.1.4.4 (http://tree.bio.ed.ac.uk/software/figtree).

### Morphospace and morphological disparity

We performed the morphospace analyses of Palaeontinidae and Dunstaniidae on the forewing morphological dataset (morphospace matrix 1) consisting of 86 characters of 76 species (complied herein; data S5), by periods and clades, respectively. Two multivariate morphospaces were ordinated using both PCoA and NMDS that exhibit coincident results. Both the Maximum Observable Rescaled Distance matrix and Generalized Euclidean Distance matrix were calculated and applied to PCoA ordination. To compare morphological disparity between early and late Palaeontinidae, three widely used disparity metrics were calculated: the sum of ranges (SOR), the sum of variances (SOV), and the median distance from centroids (MDC) (*26*, *69*–*71*). The sum of ranges captures the spread of the distribution of a group, and sum of variances represents the total dissimilarity among specimens of a group in morphospace. Both sum of ranges and variances describe the total size of a morphospace, of which larger values indicate more extreme trait combinations (*26*, *69*–*71*). The median distance from centroids represents the median spacing of individual specimens from the morphospace centroid (*26*, *70*).

We performed the analyses using the free software R. 4.0.4, Claddis for PCoA (*72*), vegan for NMDS (*73*), disRity for disparity analysis (*74*), and ggplot (*75*) and Phytools for plotting (*76*). Given the uneven species numbers among these groups, Dunstaniidae was excluded from the disparity indices. A *t* test was used to test whether the disparity matrices were statistically different among major groups using the test.dispRity function in the R package disRity (*74*). To test whether some groups were statistically distinct from other groups in morphospace occupation, a nonparametric multivariate analysis of variance (PERMANOVA) test was performed using the adonis function of the R package vegan (*73*).

### Forewing shape analyses

We conducted geometric morphometric analysis on a dataset of unscaled forewing outlines, covering 67 species that have well-preserved forewings (fig. S28 and data S1). We measured shape variation of forewings using elliptical Fourier descriptor (EFD) analysis in the R program Momocs (*77*) by placing pseudolandmarks around a closed outline (that is, forewing shape) and eliminating size as a variable. A total of 300 points (autoassigned by the software) were established for outlining the forewings. The first harmonic was used to normalize the harmonic coefficients and make them invariant to size and rotation, and seven harmonics gather 99% of the harmonic power here. This approach allows quantifiable analysis of shape when there are few or no identifiable homologous landmarks (*11*, *77*, *78*). The EFD uses the first ellipse to normalize for rotation, translation, size, and orientation and then uses harmonic coefficients for subsequent statistical analysis, PCA, and visualization.

To further explore the forewing shape evolution of Palaeontinidae and Dunstaniidae, we measured the forewing area and the proportion of several partitioning areas that greatly contribute to forewing shape, including costal area, extended costal rea, and clavus (figs. S5 and S6 and data S6). We then calculated the area proportion of the costal area to the forewing (S*_costal_), the extended costal area to the forewing (*Peco*), and the clavus to the forewing (S*_clavus_). All measurements were calculated using ImageJ 1.53k (*79*).

### Modeling insect flight performance

Estimating locomotory performance from fossil records is notoriously difficult, because information about movement kinematics is absent. Therefore, we developed a simplified qualitative insect flight model to estimate how flight performance of Palaeontinidae and Dunstaniidae scales with variations in body and wing morphology. The primary flight performance metrics under evolutionary pressure in a predator-prey arms race are locomotory speed and maneuverability (*4*, *80*). We therefore developed flight performance models for quantifying both.

In steady horizontal flight, aerodynamic lift required for weight support scales as (*7*)

$$L=½\;\rho_{air}U^{2}{\mathrm{SC}}_{L}=m_{\text{body}}g$$ (1)

where *L* is aerodynamic lift, ρ_air_ is air density, *U* is flight speed, *S* is wing surface area, *C*_L_ is the wingbeat-average lift coefficient of the wings, *m*_body_ is body mass, and *g* is gravitational acceleration. Rewriting this equation shows that flight speed *U* scales with the square root of wing loading *WL*, defined as ratio of body mass and wing surface area (*WL* = *m*_body_*/S*). Thus, we used wing loading to estimate the flight speed of flying Palaeontinidae [*U* ~ √(*WL*)]. Here, we did not take account of any effect of variations in wingbeat-average lift coefficient that could result from differences in wingbeat kinematics.

The power required for both high-speed flight and rapid flight maneuvers is produced by the flight power muscles. This power available from the muscles scales linearly with the muscle mass (*P*_avail_ ~ *m*_muscle_) (*37*). Because flying animals need to generate at foremost weight support (Eq. 1), it is useful for comparative research to normalize the available flight power with body mass (P*_avail_ ~ *P*_avail_/*m*_body_). The resulting body mass–normalized available power thus scales linearly with the equivalent ratio between the muscle mass and total body mass as m*_muscle_ = *m*_muscle_ */m*_body_. Here, we used this relative muscle mass to estimate variations in relative flight power available between Palaeontinidae, required for producing both high-speed flight and rapid flight maneuvers.

Maximum flight speed and maneuverability depend not only on flight power available but also on power required for flight *P*_req_. In flapping flight, the instantaneous power required to flap a wing equals the product of aerodynamics drag *D* and the wing speed, in the body reference frame. This drag experienced by a flapping wing depends on both wingbeat kinematics and morphology. Arguably, one of the primary morphological parameters that affect drag on a wing is the wing aspect ratio, as lift-induced drag tends to scale inversely with this metric (*48*). Here, aspect ratio *AR* is defined as the ratio between the wingspan *b* and mean chord length *c* as *AR* = *b*/*c* (fig. S25). Thus, a slender high–aspect ratio wing produces low lift-induced drag, resulting in a reduced power requirement and consequently efficient flight (*41*, *43*, *48*, *81*). Here, we therefore used wing aspect ratio as a metric for flight efficiency, which enhances both maximum flight speed and maneuverability at a given flight power availability.

Note that we here do not take account of any effect of variations wingbeat kinematics on the flight performance of extinct cicadas, as we cannot estimate kinematics from fossil records. Instead, we use our simplified qualitative aerodynamic models to estimate how variations in morphology only affect relative variations in flight performance.

### Estimating flight performance metrics from body and wing morphology

With the above defined simplified insect flight model, we can test how variations in body and wing morphology affect flight speed and maneuverability, on the basis of the morphological parameters wing loading *WL*, relative flight power muscle mass m*_muscle_, and wing aspect ratio *AR*.

We estimated the wing morphology parameters on an assembled dataset of scaled forewing outlines with 65 species that have well-preserved forewings (data S7) and a dataset of scaled combined wing outlines of 21 species that have well-preserved forewings and hind wings (data S8). The combined wing outline was created by using the nonoverlapping portions of the rotated forewing and hind wing. To ensure comparability, wing measurements among all species were taken after reorienting each wing to a consistent position: The forewings and combined wings were rotated so that their long axis was perpendicular to the long axis of the body. Using the method defined by Ellington (*43*), we estimated the following wing parameters (fig. S25): wingspan (*b*), wing area (*S*), average chord (*c* = *S*/*b*), and aspect ratio (*AR* = *b*/*c* = *b*^2^/*S*) of the forewings and of the forewing and hind wing combined (*AR*_forewing_ and *AR*_combined_, respectively).

We estimated the body morphology parameters on the well-preserved bodies of 22 species (data S9). We further estimated body and muscle mass using simplified geometric body models (fig. S25). We assumed that the density of the insect was equal to that of water, and thus, body mass is equal to *m*_body_ = ρ_water_*V*_body_, where ρ_water_ and *V*_body_ are water density and the volume of the body model, respectively. As the thorax of cicadas mostly constitutes flight power muscles, we estimated the muscle mass as *m*_muscle_ = ρ_water_*V*_mesothorax_, where *V*_mesothorax_ is the volume of the mesothorax model. To calculate these volumes, we measured the following body morphology parameters: thorax width (*w*_thorax_), thorax length (*l*_thorax_), and abdominal length (*l*_abdome_). Thorax length constitutes prothoracic length (*l*_pt_) and mesothoracic length (*l*_mt_); metathoracic length was not measured because it is covered by the mesonotum. Body volumes were measured on the basis of approximate geometries and their combination, which are slightly adjusted in early and late Palaeontinidae in consideration of their body variation (fig. S25). All morphometric parameters were measured using WingImageProcessor in MATLAB (https://biomech.web.unc.edu/wing-image-analysis/), ImageJ 1.53k (*79*), and tpsDig232 (*82*), on our complied dataset (table S9).

### Allometry analysis

To examine the potential relationship between size and flight abilities in Palaeontinidae, we tested how aspect ratio (*AR*) varied with wingspan (*b*). This shows clearly different trends between early and late Palaeontinidae species. To test whether early and late Palaeontinidae differed in terms of their *AR*-*b* regression slopes, we conducted ANCOVAs using the aov function in the R package stats. To reduce the skewness of the measurement variable, we log-transformed the parameters *b* and *AR* before conducting the analyses (*83*). To meet the assumptions of ANCOVA, we checked the normality of residuals and plotted them using the qqPlot function in the R package car (*W* = 0.9669, *P* value = 0.1202; fig. S29). We conducted Levene’s test of equality of variance using the bartlett.test function in the R package stats (Bartlett’s *K*-squared = 0.43441, df = 1, *P* value = 0.5098). To meet the assumptions of the Pearson test, we checked the normality of residuals using the Shapiro-Wilk test with the shapiro.test function in the R package stats. We also plotted the residuals using the ggqqplot function in the R package ggpubr. Both log*_AR_* and log*_b_* follow the normal distribution in early and late Palaeontinidae (log*_AR_* and log*_b_* in early Palaeontinidae: *W* = 0.94825, *P* value = 0.1285, *W* = 0.98737, *P* value = 0.9637; log*_AR_* and log*_b_* in late Palaeontinidae: *W* = 0.9669, *P* value = 0.568, *W* = 0.94741, *P* value = 0.219; fig. S30).

## Supplementary Materials

### The PDF file includes:

Supplementary Text Figs. S1 to S30 Tables S1 to S10 Legend for movie S1 Legends for data S1 to S9 References
