# Supplementary material for: Enhanced flight performance and adaptive evolution of Mesozoic giant cicadas
Source: Sci Adv. 2024 Oct 25;10(43):eadr2201. doi: 10.1126/sciadv.adr2201 (PMC11506159; doi:10.1126/sciadv.adr2201)
Supplement: Supplementary file 1 — Supplementary Text Figs. S1 to S30 Tables S1 to S10 Legend for movie S1 Legends for data S1 to S9 References [file sciadv.adr2201_sm.pdf]

Science Advances
AAAS

Supplementary Materials for

Enhanced flight performance and adaptive evolution of Mesozoic giant cicadas

Chunpeng Xu *et al.*

Corresponding author: Bo Wang, bowang@nigpas.ac.cn

Sci. Adv. **10**, eadr2201 (2024)
DOI: 10.1126/sciadv.adr2201

The PDF file includes:

Supplementary Text
Figs. S1 to S30
Tables S1 to S10
Legend for movie S1
Legends for data S1 to S9
References

Other Supplementary Material for this manuscript includes the following:

Movie S1
Data S1 to S9

Supplementary Text

Material and methods

Venational terminology

There is no consensus on the venation pattern and vein homology of Cicadomorpha. Various authors have used different names and interpretations (*84–86*). Nel et al. (*87*) proposed a new interpretation of the wing venation pattern for all Paraneoptera, assuming that CuA is fused with M+R stem at the wing base and connected with CuP by a specialized crossvein cua-cup. This interpretation is different from traditional viewpoints (*88*, *89*). The venational terminologies used herein are slightly modified from Nel et al. (*87*), and first longitudinal vein on clavus is treated as Pcu rather than A1 following Schubnel et al. (*90*). Additionally, in Paraneoptera, MA is completely fused with R, resulting in only MP being present. Therefore, we have treated the independent media vein as MP instead of M (*91*).

Taxa selected for phylogenetic and morphospace analyses

Seventy-six undisputed species are selected for our analyses with a Num-Species correspondence (Table S10). In order to provide more insight into the diversity and evolutionary history of Palaeontinidae, two *conformis* species and two undescribed species are included herein (Supplementary Figs. 2, 4).

Characters used for phylogenetic analyses

For phylogenetic analyses, three matrices were assembled based on the following characters.

Forewing

1. Marginal membrane: 0, absent or indistinct; 1, distinct.

2. Shape of costal margin: 0, smoothly curved; 1, strongly curved near wing base; 2, nearly straight.

3. Areal proportion of extended costal area to forewing: 0, large (>15%); 1, moderate (15%-8%); 2, reduced (<8%).

4. Breadth of costal area: 0, broad, 1, narrow.

5. Status of CP: 0, short and not distinct or absent; 1, long to apex of costal area.

6. ScP with veinlets or not: 0, no; 1, yes.

7. Basal part of ScP: 0, fused with R+MP; 1, parallel or sub-parallel to R+MP; 2, strongly curved and not parallel to R+MP.
8. ScP fused with R+MP/R/RA in prenodal area: 0, at wing base with R+MP; 1, at R; 2, at RA; 3, at bifurcation of R+MP+CuA; 4, at bifurcation of R
9. ScP in postnodal area: 0, short or absent; 1, independent and long; 2, fused with RA in distal part.
10. Area formed by ScP and R/RA: 0, absent; 1, narrow; 2, broad.
11. Shape of anterior margin: 0, slightly curved, slightly sinuous or nearly straight; 1, sinuous.
12. Wing apex: 0, rounded; 1, acute.
13. Division of R+MP: 0, very close to or at division of MP+CuA; 1, at wing base.
14. Position of bifurcation of R: 0, not at nodal line or at division of R+MP; 1, at nodal line; 2, at division of R+MP.
15. Relative position of bifurcation of R to nodal line and wing base: 0, close to nodal line; 1, at nodal line; 2, close to wing base.
16. Shape of RA in prenodal area: 0, straight, slightly curved or just curved at fusion with ScP; 1, arched.
17. Shape of RA in postnodal area: 0, strongly curved posteriorly; 1, nearly straight or smoothly curved anteriorly; 2, straight and short.
18. Number of terminal branches of RA: 0, one; 1, two.
19. RP at nodal line: 0, strongly curved; 1, nearly straight.
20. Connectivity of RP and MP1: 0, fused for a short interval; 1, by crossvein rp-mp.
21. Direction of outer margin: 0, nearly vertical; 1, oblique.
22. Shape of outer margin: 0, smoothly curved or nearly straight; 1, ripple-like.
23. Position of stem MP: 0, almost at middle of wing width; 1, migrated towards anterior margin.
24. Shape of stem MP: 0, nearly straight; 1, strongly curved anteriorly at base; 2, strongly curved posteriorly at base.
25. Length of stem MP: 0, long, at least three times as long as stem of MP1+2 and MP3+4; 1, short, commonly equal to or even shorter than MP1+2 and/or MP3+4.
26. Position of bifurcation of MP: 0, near wing tip; 1, near middle of wing; 2, near wing base.
27. Position of bifurcation of MP1+2: 0, near wing tip; 1, near middle of wing length or slightly basad of middle of wing length.

28. Position of bifurcation of MP3+4: 0, near wing tip; 1, near middle of wing length or slightly basad of middle of wing length.
29. Shape of MP1+2: 0, nearly straight; 1, strongly curved at base; 2, curved at nodal line.
30. Stem MP3+4: 0, present; 1, absent.
31. Position of bifurcation of MP3+4: 0, far away from nodal line; 1, at nodal line.
32. Direction of stem MP3+4: 0, oblique; 1, nearly vertical.
33. Shape of stem MP3+4: 0, nearly straight or slightly curved; 1, strongly curved at base.
34. Number of terminal branches of MP3: 0, single; 1, two.
35. Distal part of MP1, MP2, MP3, and MP4: 0, not parallel; 1, parallel.
36. Crossvein mp-cua in discal cell: 0, absent; 1, present.
37. Shape of crossvein mp-cua in discal cell: 0, nearly straight; 1, smoothly curved; 2, sinuous; 3, three crossveins fused at one point.
38. Connection of crossvein mp4-cua (or mp3+4-cua) with MP: 0, at about middle of MP3+4; 1, at MP3+4 near bifucation of MP; 2, at MP4; 3, at MP3+4 near bifurcation of MP3+4.
39. Connection of crossvein mp4-cua (or mp3+4-cua) with CuA: 0, very close to or at nodal line; 1, basad of nodal line; 2, connecting to CuA1 instead of stem CuA.
40. Direction of crossvein mp4-cua (or mp3+4-cua): 0, oblique; 1, almost longitudinal; 2, oblique along nodal line and almost longitudinal beyond nodal line; 3, almost vertical.
41. Shape of crossvein mp4-cua (or mp3+4-cua): 0, smoothly curved or nearly straight; 1, strongly curved at nodal line.
42. Crossvein mp4-cua (or mp3+4-cua) fused with nodal line: 0, for a short interval; 1, not fused; 2, for a very long interval; 3, almost fully fused.
43. Direction of basal cell: 0, almost longitudinal; 1, oblique.
44. Shape of basal cell: 0, quadrangular (trapezoidal or rectangular); 1, nearly triangular.
45. Size of basal cell: 0, extremely small; 1, moderate.
46. Direction of discal cell: 0, almost longitudinal; 1, oblique.
47. Size of discal cell: 0, moderate; 1, extremely large.
48. Size ratio of antenodal and postnodal region of discal cell: 0, more than four; 1, less or equal to one; 2, more than two and less than four.
49. Shape of discal cell: 0, elongate taper-shaped; 1, almost rectangular; 2, hexagonal or polygonal.

50. Shape of antenodal region of discal cell: 0, nearly triangular; 1, nearly trapezoidal; 2, nearly semi-circular; 3, almost rectangular; 4, irregular polygonal.
51. Shape of postnodal region of discal cell: 0, nearly triangular; 1, quadrangular (trapezoidal or nearly trapezoidal); 2, rectangular; 3, crescentic; 4, absent.
52. Crossvein imp of MP3 and MP4: 0, absent; 1, present.
53. Posteroapical angle: 0, nearly no angle; 1, obtuse; 2, nearly right-angled.
54. Direction of posterior margin: 0, horizontal; 1, oblique.
55. Posterior margin near nodal line: 0, not indented; 1, indented; 2, strongly indented.
56. CuA fused with nodal line or not: 0, no; 1, yes.
57. Shape of CuA at junction with crossvein cua-cup: 0, straight; 1, geniculate; 2, smoothly curved.
58. Length of stem CuA after junction with nodal line: 0, short; 1, absent; 2, extremely long.
59. Length of CuA2: 0, normal; 1, extremely short.
60. Shape of CuA2 beyond nodal line: 0, sinuous; 1, slightly curved, sinuous or nearly straight; 2, extremely sinuous; 3, strongly curved.
61. Distal part of CuA1 and CuA2: 0, parallel or sub-parallel; 1, not parallel.
62. CuP after junction with crossvein cua-cup: 0, straight or smoothly curved; 1, sinuous.
63. Crossvein cua-cup connecting to R+MP+CuA/ MP+CuA/ CuA: 0, R+MP+CuA; 1, MP+CuA; 2, CuA.
64. Shape of crossvein cua-cup: 0, curved at basal/middle part; 1, nearly straight or slightly curved.
65. Position of junction of crossvein cua-cup with R+MP+CuA/ MP+CuA/ CuA: 0, very close to bifurcation of MP+CuA; 1, far away from bifurcation of MP+CuA.
66. Shape of Pcu: 0, sinuous; 1, straight or slightly curved.
67. Distal part of Pcu fused with CuP for a distance or not: 0, no; 1, yes.
68. Shape of anal margin: 0, curved near wing base; 1, smoothly curved.
69. Size of clavus: 0, large; 1, moderate, 2, reduced.
70. Breadth of clavus: 0, broad, 1, narrow.
71. Origin of A1: 0, at anal margin far away from wing base; 1, near or at wing base.
72. Branches of A 1: 0, single; 1, two.

73. Length of the second branch of A1: 0, very short; 1, almost 1/2 as long as the first branch of A1.
74. Direction of nodal line: 0, nearly vertical; 1, obviously oblique.
75. Shape of nodal line: 0, nearly straight; 1, curved and/or sinuous.
76. Position of nodal line: 0, distad of middle of wing length; 1, near middle of wing length; 2, migrated towards wing base.
77. Shape of nodal line in discal cell: 0, straight or slightly curved; 1, strongly sinuous.

Hind wing

78. Relative size of forewing to hind wing: 0, obviously shorter than 1.8 times length of hind wing; 1, at least 1.8 times length of hind wing.
79. Marginal membrane: 0, absent; 1, present.
80. Shape of wing: 0, elongated; 1, nearly rounded; 2, fan-shaped.
81. Ratio of wing length/width: 0, less than 1.5; 1, more than 1.5.
82. Shape of costal margin: 0, strongly curved at or near middle; 1, strongly curved at base; 2, not curved.
83. Direction of distal part of costal margin: 0, obviously oblique; 1, slightly oblique or nearly horizontal.
84. Shape of anterior margin: 0, strongly curved at wing indentation and sinuous; 1, nearly straight or smoothly curved at wing indentation.
85. Anterior margin at wing indentation: 0, strongly indented; 1, smoothly indented; 2, without indentation.
86. Shape of wing apex: 0, sharp; 1, rounded.
87. Shape of anal margin: 0, smoothly curved; 1, strongly curved in apical part.
88. Anal margin at CuP: 0, not indented; 1, indented.
89. Shape of posteroapical angle: 0, smoothly curved; 1, rounded; 2, acute.
Position of posteroapical angle.
90. Status of costal area: 0, distinctly present; 1, strongly reduced or absent.
91. Breadth of costal area: 0, narrow, not up to 1/8th wing length; 1, broad, at least 1/8th wing length.
92. Length of costal area: 0, long, about 1/2 wing length; 1, short, about 1/3rd wing length.
93. Direction of costal area: 0, nearly horizontal; 1, slightly oblique.

94. Position of bifurcation of R: 0, extremely closed to or at wing indentation; 1, near wing base.
95. Number of independent terminal branches of RA: 0, at most two; 1, three.
96. Shape of basal part of last terminal branch of RA: 0, almost straight; 1, strongly curved.
97. Ending position of last terminal branch of RA: 0, near wing apex; 1, at about middle of wing.
98. Connection of RP and MP1: 0, by crossvein rp-mp; 1, fused at a point; 2, fused for a short interval.
99. Size and shape of medial area: 0, large and nearly rhomboidal; 1, large and oblong; 2, moderate; 3, reduced.
100. Position of bifurcation of MP: 0, distant away from wing base and near wing indentation; 1, near wing base and distant away from wing indentation; 2, almost at wing base; 3, at median position between wing base and wing indentation.
101. Relative position of bifurcation of MP to bifurcation of R: 0, almost at same level or slightly beyond bifurcation of R (near wing indentation); 1, basad of bifurcation of R; 2, almost at same level (at or very near wing base).
102. Length of stem MP: 0, extremely long; 1, moderate (longer than MP1+2); 2, short; 3, extremely short or absent.
103. Basal part of MP: 0, fused with RA and Sc; 1, fused with CuA; 2, independent.
104. Stem MP1+2: 0, absent; 1, present but extremely short; 2, present and long.
105. Stem MP2+3+4: 0, absent; 1, short; 2, long (longer than stem M).
106. Shape of MP3+4 at connection with crossvein mp3+4-cua or CuA: 0, geniculate or curved; 1, nearly straightly.
107. Number of terminal branches of MP3+4: 0, two; 1, one.
108. Relative branching position of MP3+4 to CuA: 0, distad of the latter; 1, almost at same level; 2, apparently basad of latter.
109. Connection of MP and CuA: 0, by crossvein mp3+4-cua; 1, fused at one point or for a short interval; 2, discontiguous; 3, by crossvein mp4-cua.
110. Position of connection of stem CuA and MP3+4: 0, far away from departure of MP3+4; 1, at or extremely closed to departure of MP3+4.
111. Presence of crossvein mp3+4-cua1: 0, absent; 1, present.
112. CuA at junction with crossvein mp3+4-cua or MP3+4: 0, nearly straight or slightly curved; 1, obviously geniculate.

113. CuP fused with CuA at base or not: 0, not; 1, fused at one point; 2, fused completely.
114. Basal part of Pcu: 0, fused with A1; 1, independent.
115. Number of terminal branches of Pcu: 0, one; 1, two.
116. Direction of Pcu: 0, oblique; 1, nearly vertical.
117. Direction of anal area: 0, oblique; 1, nearly vertical.
118. Presence of A1 or not: 0, present; 1, absent.
119. Distal part of A1: 0, independent; 1, fused with Pcu.

<u>Characters used for morphospace analyses</u>

All characters used for morphospace analyses are from forewings

1. Marginal membrane: 0, absent or indistinct; 1, distinct.
2. Shape of costal margin: 0, smoothly curved; 1, strongly curved near wing base; 2, nearly straight.
3. Areal proportion of extended costal area to forewing: 0, large (>15%); 1, moderate (15%-8%); 2, reduced (<8%).
4. Breadth of costal area: 0, broad, 1, narrow.
5. Status of CP: 0, short and not distinct or absent; 1, long to apex of costal area.
6. ScP with veinlets or not: 0, no; 1, yes.
7. Basal part of ScP: 0, fused with R+MP; 1, parallel or sub-parallel to R+MP; 2, strongly curved and not parallel to R+MP.
8. ScP fused with R+MP/R/RA in prenodal area: 0, at wing base with R+MP; 1, at R; 2, at RA; 3, at bifurcation of R+MP+CuA; 4, at bifurcation of R
9. ScP in postnodal area: 0, short or absent; 1, independent and long; 2, fused with RA in distal part.
10. Area formed by ScP and R/RA: 0, absent; 1, narrow; 2, broad.
11. Shape of anterior margin: 0, slightly curved, slightly sinuous or nearly straight; 1, sinuous.
12. Wing apex: 0, rounded; 1, acute.
13. Division of R+MP: 0, very close to or at division of MP+CuA; 1, at wing base.
14. Position of bifurcation of R: 0, not at nodal line or at division of R+MP; 1, at nodal line; 2, at division of R+MP.

15. Relative position of bifurcation of R to nodal line and wing base: 0, close to nodal line; 1, at nodal line; 2, close to wing base.
16. Shape of RA in prenodal area: 0, straight, slightly curved or just curved at fusion with ScP; 1, arched.
17. Shape of RA in postnodal area: 0, strongly curved posteriorly; 1, nearly straight or smoothly curved anteriorly; 2, straight and short.
18. Number of terminal branches of RA: 0, one; 1, two.
19. RP at nodal line: 0, strongly curved; 1, nearly straight.
20. Connectivity of RP and MP1: 0, fused for a short interval; 1, by crossvein rp-mp.
21. Direction of outer margin: 0, nearly vertical; 1, oblique.
22. Shape of outer margin: 0, smoothly curved or nearly straight; 1, ripple-like.
23. Position of stem MP: 0, almost at middle of wing width; 1, migrated towards anterior margin.
24. Shape of stem MP: 0, nearly straight; 1, strongly curved anteriorly at base; 2, strongly curved posteriorly at base.
25. Length of stem MP: 0, long, at least three times as long as stem of MP1+2 and MP3+4; 1, short, commonly equal to or even shorter than MP1+2 and/or MP3+4.
26. Position of bifurcation of MP: 0, near wing tip; 1, near middle of wing; 2, near wing base.
27. Position of bifurcation of MP1+2: 0, near wing tip; 1, near middle of wing length or slightly basad of middle of wing length.
28. Position of bifurcation of MP3+4: 0, near wing tip; 1, near middle of wing length or slightly basad of middle of wing length.
29. Shape of MP1+2: 0, nearly straight; 1, strongly curved at base; 2, curved at nodal line.
30. Stem MP3+4: 0, present; 1, absent.
31. Position of bifurcation of MP3+4: 0, far away from nodal line; 1, at nodal line.
32. Direction of stem MP3+4: 0, oblique; 1, nearly vertical.
33. Shape of stem MP3+4: 0, nearly straight or slightly curved; 1, strongly curved at base.
34. Number of terminal branches of MP3: 0, single; 1, two.
35. Distal part of MP1, MP2, MP3, and MP4: 0, not parallel; 1, parallel.
36. Crossvein mp-cua in discal cell: 0, absent; 1, present.
37. Shape of crossvein mp-cua in discal cell: 0, nearly straight; 1, smoothly curved; 2, sinuous; 3, three crossveins fused at one point.

38. Connection of crossvein mp4-cua (or mp3+4-cua) with MP: 0, at about middle of MP3+4; 1, at MP3+4 near bifucation of MP; 2, at MP4; 3, at MP3+4 near bifurcation of MP3+4.
39. Connection of crossvein mp4-cua (or mp3+4-cua) with CuA: 0, very close to or at nodal line; 1, basad of nodal line; 2, connecting to CuA1 instead of stem CuA.
40. Direction of crossvein mp4-cua (or mp3+4-cua): 0, oblique; 1, almost longitudinal; 2, oblique along nodal line and almost longitudinal beyond nodal line; 3, almost vertical.
41. Shape of crossvein mp4-cua (or mp3+4-cua): 0, smoothly curved or nearly straight; 1, strongly curved at nodal line.
42. Crossvein mp4-cua (or mp3+4-cua) fused with nodal line: 0, for a short interval; 1, not fused; 2, for a very long interval; 3, almost fully fused.
43. Direction of basal cell: 0, almost longitudinal; 1, oblique.
44. Shape of basal cell: 0, quadrangular (trapezoidal or rectangular); 1, nearly triangular.
45. Size of basal cell: 0, extremely small; 1, moderate.
46. Direction of discal cell: 0, almost longitudinal; 1, oblique.
47. Size of discal cell: 0, moderate; 1, extremely large.
48. Size ratio of antenodal and postnodal region of discal cell: 0, more than four; 1, less or equal to one; 2, more than two and less than four.
49. Shape of discal cell: 0, elongate taper-shaped; 1, almost rectangular; 2, hexagonal or polygonal.
50. Shape of antenodal region of discal cell: 0, nearly triangular; 1, nearly trapezoidal; 2, nearly semi-circular; 3, almost rectangular; 4, irregular polygonal.
51. Shape of postnodal region of discal cell: 0, nearly triangular; 1, quadrangular (trapezoidal or nearly trapezoidal); 2, rectangular; 3, crescentic; 4, absent.
52. Crossvein imp of MP3 and MP4: 0, absent; 1, present.
53. Posteroapical angle: 0, nearly no angle; 1, obtuse; 2, nearly right-angled.
54. Direction of posterior margin: 0, horizontal; 1, oblique.
55. Posterior margin near nodal line: 0, not indented; 1, indented; 2, strongly indented.
56. CuA fused with nodal line or not: 0, no; 1, yes.
57. Shape of CuA at junction with crossvein cua-cup: 0, straight; 1, geniculate; 2, smoothly curved.
58. Length of stem CuA after junction with nodal line: 0, short; 1, absent; 2, extremely long.

59. Length of CuA2: 0, normal; 1, extremely short.

60. Shape of CuA2 beyond nodal line: 0, sinuous; 1, slightly curved, sinuous or nearly straight; 2, extremely sinuous; 3, strongly curved.

61. Distal part of CuA1 and CuA2: 0, parallel or sub-parallel; 1, not parallel.

62. CuP after junction with crossvein cua-cup: 0, straight or smoothly curved; 1, sinuous.

63. Crossvein cua-cup connecting to R+MP+CuA/ MP+CuA/ CuA: 0, R+MP+CuA; 1, MP+CuA; 2, CuA.

64. Shape of crossvein cua-cup: 0, curved at basal/middle part; 1, nearly straight or slightly curved.

65. Position of junction of crossvein cua-cup with R+MP+CuA/ MP+CuA/ CuA: 0, very close to bifurcation of MP+CuA; 1, far away from bifurcation of MP+CuA.

66. Shape of Pcu: 0, sinuous; 1, straight or slightly curved.

67. Distal part of Pcu fused with CuP for a distance or not: 0, no; 1, yes.

68. Shape of anal margin: 0, curved near wing base; 1, smoothly curved.

69. Size of clavus: 0, large; 1, moderate, 2, reduced.

70. Breadth of clavus: 0, broad, 1, narrow.

71. Origin of A1: 0, at anal margin far away from wing base; 1, near or at wing base.

72. Branches of A 1: 0, single; 1, two.

73. Length of the second branch of A1: 0, very short; 1, almost 1/2 as long as first branch of A1.

74. Direction of nodal line: 0, nearly vertical; 1, obviously oblique.

75. Shape of nodal line: 0, nearly straight; 1, curved and/or sinuous.

76. Position of nodal line: 0, distad of middle of wing length; 1, near middle of wing length; 2, migrated towards wing base.

77. Shape of nodal line in discal cell: 0, straight or slightly curved; 1, strongly sinuous.

78. RP ending position on wing margin: 0, before apex; 1, at apex; 2, after apex.

79. Relative ending position of RA and RP: 0, RA slightly before RP; 1, at same level; 2, RA distinctly before RP.

80. Relative ending position of RA and MP1: 0, RA before MP1; 1, at same level; 2, after MP1.

81. Relative position of bifurcation of MP to bifurcation of R: 0, MP before R; 1, at same level; 2, after R.

82. Relative position of bifurcation of MP to crossvein mp-cua fusing position with CuA: 0, bifurcation of MP before the latter; 1, at same level; 2, after the letter.
83. Relative position of bifurcation of MP3+4 to ending position of nodal line on anterior: 0, bifurcation of MP3+4 before the latter; 1, same; 2, after.
84. Relative position of bifurcation of MP3+4 to posterior apex: 0, bifurcation of MP3+4 before the latter; 1, at same level; 2, after.
85. Relative position of posterior apex to wing length: 0, before middle; 1, at middle; 2, after middle.
86. Relative end position of nodal line on anterior and posterior margins: 0, ending on anterior before posterior margin; 1, at same level; 2, after posterior margin.

Parsimony analyses using PAUP Version 4.0a

Consensus tree logs (Parsimony analyses) on Phylogenic Matrix 1 (*Austroprosboloides vandijki* was set as outgroup)

Bootstrap method with heuristic search:

Number of bootstrap replicates = 1000

Starting seed = generated automatically

Number of characters resampled in each replicate = 119

Optimality criterion = parsimony

Character-status summary:

Of 119 total characters:

All characters are of type 'unord'

All characters have equal weight

10 characters are parsimony-uninformative

Number of parsimony-informative characters = 109

Starting tree(s) obtained via stepwise addition

Addition sequence: random

Number of replicates = 100

Starting seed = generated automatically

Number of trees held at each step = 5

Branch-swapping algorithm: tree-bisection-reconnection (TBR) with reconnection limit = 8

Steepest descent option not in effect

Initial 'Maxtrees' setting = 100 (will be auto-increased by 100)

Branches collapsed (creating polytomies) if maximum branch length is zero

'MulTrees' option not in effect; only 1 tree will be saved per replicate

No topological constraints in effect

Trees are unrooted

Bootstrap 50% majority-rule consensus tree on Phylogenic Matrix 1 (*Austroprosboloides vandijki* was set as outgroup)

```
/------------------------------------------------------------------------------------ Austroprosboloides vandijki(1)
|
| /---------------------------------------------------------------------------------- Australiastroprosbole maculata(2)
| |
| |    /--------------------------------------------------------------------------- Gallodunstania grauvogeli(3)
| |    |
| |    |     /-------------------------------------------------------------------- Fletcheriana triassica(4)
| |    |     |
| |    |     |                                              /------ Papiliontina dracomima(7)
| |    |     |                                              |
| |    |     |                                           /90+  /--- Papiliontina machaon(8)
| |    |     |                                           |  \44+
| |    |     | /--------------------------32---------------------------+     \--- Papiliontina spectans(9)
| |    |     | |                                         |
| |    |     | |                                         |    /--- Karoontina magna(10)
| |    |     | |                                         \-67--+
| |    |     | |                                              \--- Hallakkungis amisanus(11)
| |    |     | |
| |    |     | |                                            /------ Plachutella exculpta(12)
| |    |     | |                                            |
| |    |     | |                                         /1-+  /--- Plachutella derupta(20)
| |    |     | |                                         |  \15+
| |    |     | |                                      /62+     \--- Plachutella magica(35)
| |    |     | |                                      |  |
| |    |     | |                          /-----35-----+  \--------- Plachutella rotundata(19)
| |    |     | |                          |          |
| |    |     | |                          |          |       /--- Suljuktaja turkestanensis(14)
| |    |     | |                          |          \---57---+
| |    |     | |                          |                  \--- Shurabocossus gigas(18)
| |    |     | |                          |
| |    |  /98-+  |                          |       /---------------- Synapocossus sciacchitanoae(25)
| |    |  |  |  | |                          |       |
| |    |  |  |  | |                          |       |  /------------ Sinopalaeocossus trinervus(29)
| |    |  |  |  | |                       /1-+     /51+   |
| |    |  |  |  | |                       | |    |  |  |       /--- Sinopalaeocossus longicaulis(30)
| |    |  |  |  | |                       | |    | \50-+     /55+
| |    |  |  |  | |                       | |    |     |    | \--- Sinopalaeocossus fangi(32)
| |    |  |  |  | |                       | |    |     | /24+
| |    |  |  |  | |                       | | /26+     | | \------ Sinopalaeocossus eumorphus(33)
| |    |  |  |  | |                       | | | |     \46+
| |    |  |  |  | |                       | | | |        |    /--- Sinopalaeocossus laevis(31)
| |    |  |  |  | |                       | | | |        \-36--+
| |    |  |  |  | |                       | \9-+ |              \--- Sinopalaeocossus amoenus(34)
| |    |  |  |  | |                       |    | |
| |    |  |  |  | |                       |    | \------------------- Neimenggucossus normalis(42)
| |    |  |  |  | |                       |    |
| |    |  |  |  | |                    /2-+    \---------------------- Kallicossus ningchengensis(47)
| |    |  |  |  | |                    | |
| |    |  |  |  | |                    | |                    /--- Palaeocossus jurassicus(17)
| |    |  |  |  | |                    | |                 /44+
```

```
| |    | |  | |                              | |                   |  \--- Martynovocossus bellus(46)
| |    | |  | |                              | |                /0-+
| |    | |  | |                              | |                |  |  /--- Martynovocossus punctulosus(43)
| |    | |  | |                              | |                |  \1-+
| |    | |  \98+                               | |              /8-+     \--- Martynovocossus cheni(45)
| |  /90+  |      |                              | |              | |
| | | | |     |                            /2-+  |          /10-+  \--------- Martynovocossus decorus(44)
| | | | |     |                            | | |          |  |
| | | | |     |                            | | |          |  \------------ Martynovocossus turgaiensis(22)
| | | | |     |                            | |  \-----1-----+
\--+  | | |     |                              | |             |           /--- Suljuktocossus yinae(26)
  | | | |     |                            | |             |        /6-+
  | | | |     |                            | |             |        |  \--- Suljuktocossus chifengensis(28)
  | | | |     |  /------------12-------------+  |             \----1----+
  | | | |     | |                          | |                      \------ Suljuktocossus coloratus(27)
  | | | |     | |                          | |
  | | | |     | |                          |  \------------------------------- Palaeontina oolitica(24)
  | | | |     | |                          |
  | | | |     | |                          |             /------------------- Suljuktocossus prosboloides(13)
  | | | |     | |                          |             |
  | | | |     | |                          |             |  /---------------- Phragmatoecites damesi(15)
  | | | |     | |                          \------4-------+  |
  | | | |     | |                                        | |           /--- Ningchengia minuta(40)
  | | | |     | |                                        \22+   /---64---+
  | | | |     | |                                           |   |       \--- Ningchengia aspera(41)
  | | | |     | |                                           |   |
  | | | |     | |                                           \30-+     /------ Eoiocossus validus(50)
  | | | |     | |                                               |     |
  | | | |     | |                                               |  /89+  /--- Eoiocossus giganteus(51)
  | | | |     | |                                               |  |  \49+
  | | | |     | |                                               \59+     \--- Eoiocossus conchatus(52)
  | | | |     | |                                                  |
  | | | |     | |                                                  \--------- Abrocossus longus(58)
  | | | |     | |
  | | | |     | |                                                       /--- Palaeontinodes angarensis(16)
  | | | |     | |                                                    /32+
  | | | |     | |                                                    |  \--- Ijacossus suchanovae(21)
  | | | |     | |  /-------------------------10-------------------------+
  | | | |     | | |                                                     \------ Cladocossus undulatus(57)
  | | | |     | | |
  | | | |     | | |                                                     /------ Gansucossus pectinatus(23)
  | | | |     | | |                                                     |
  | | | |     | | |             /-----------------24------------------+  /--- Gansucossus typicus(48)
  | | | |     | | |             |                                       \25+
  | | | |     | | |             |                                          \--- Gansucossus luanpingensis(49)
  | | | |     | | |             |
  | | | |     \52+  |             |                                         /------ Cicadomorpha guancaishanensis(59)
  | | | |        | |            |                                        |
  | | | |        | |            |                                     /15+  /--- Cicadomorpha parula(63)
  | | | |        | |            |                                     |  \47+
  | | | |        | |            |  /--------------29--------------+     \--- Cicadomorpha milva(64)
  | | | |        | |            |  |                                  |
  | | | |        | |         /23+   |                                  |    /--- Cicadomorpha sp1(66)
  | | | |        | |         | |  |                                 \-49--+
  | | | |        | |         | |  |                                       \--- Cicadomorpha sp2(69)
  | | \52+        | |         | |  |
  | |    |        | |         | |  | /------------------------------------ Talbragarocossus jurassicus(60)
  | |    |        | |         | |  | |
  | |    |        | |         | |  | |                                 /--- Prolystra lithographica(61)
  | |    |        | |         | |  | |                              /37+
  | |    |        | |         | |  | |                              |  \--- Eocicada microcephala(62)
  | |    |        | |         | |  | |    /-----------45-----------+
  | |    |        | |         |  \17-+ |    |                        \------ Archipsyche eichstattensis(65)
  | |    |        | |         |     | |    |
  | |    |        | |         |     | |    |                     /------ Ilerdocossus prowsei(68)
  | |    |        | |         |     | |    |                     |
  | |    |        | |         |     | |    |                  /37+  /--- Ilerdocossus pulcherrima(76)
  | |    |        | |         |     | |    |                  |  \46+
  \95+    |        | |         |     | |    |                 /3-+     \--- Ilerdocossus fengningensis(79)
     |    |        | |         |     | |    |                 | |
```

```
|    |        | |          |      | |     |                 | |    /--- Pachypsyche vidali(73)
|    |        | |          |      | |     |              /-1-+  \-13--+
|    |        | |          |      | |     |              |  |       \--- Ilerdocossus villaltai(75)
|    |        | |          |      | |  /35+               |  |
|    |        | |          |      \74+  | |              |  \------------ Ilerdocossus hui(78)
|    |        | |          |        | | |                |
|    |        | |          |        | | |                |          /--- Yanocossus guoi(71)
|    |        | |          |        | | | /-----3-----+         /79+
|    |        \56+          |        | | | |          |        | \--- Miracossus ingentius(74)
|    |          |          |        | | | |          |     100+
|    |          |          |        | | | |          |     | \------ Yanocossus cf guoi(72)
|    |          |          |        | | | |          |  /19+
|    |          |          |        | | | |          |  | |    /--- Ilerdocossus exiguus(80)
|    |          |       /12+         | | | |          \-4-+  \-43--+
|    |          |       | |         | | | |              |       \--- Ilerdocossus beipiaoensis(82)
|    |          |       | |         | | \12+              |
|    |          |       | |         | |    |             \------------ Ilerdocossus dissidens(81)
|    |          |       | |         | |    |
|    |          |       | |         | |    | /------------------------- Ilerdocossus ningchengensis(77)
|    |          |       | |         | |    | |
|    |          |       | |         | |    | |                   /--- Parawonnacottella penneyi(83)
|    |          |       | |         | |    | |                /28+
|    |          |       | |        \80+    | |                 | \--- Baeocossus fortunatus(92)
|    |          |       | |           |    | | /-------8-------+
|    |          |       | |           |    \14+ |              \------ Parawonnacottella araripensis(84)
|    |          |       | |           |       | |
|    |          |       | |           |       | |             /------ Cratocossus magnus(85)
|    |          |       | |           |       | |             |
|    |          |       | |           |       | |          /44+  /--- Colossocossus loveridgei(87)
|    |          |       | |           |       \20+           | \23+
|    |          |       | |           |         |       /8-+    \--- Colossocossus giganticus(88)
|    |          |       | |           |         |       | |
|    |          |    /16+ |           |         |    /-7-+  \--------- Colossocossus bechlyi(89)
|    |          |    | | |           |         |    |  |
|    |          |    | | |           |         |  /87+   \------------ Colossocossus rugosa(86)
|    |          |    | | |           |         |  | |
|    |          |    | | |           |         \11+  \---------------- Colossocossus acutus(90)
|    |          |    | | |           |            |
|    |          |    | | |           |            \------------------- Baeocossus muratai(91)
|    |          |    | | |           |
|    |          |    | | |           |                            /--- Valdicossus chesteri(67)
|    |          |    | | |           \--------------46--------------+
|    |          |    | | |                                        \--- Valdicossus mikewebsteri(70)
|    |          |  /12+  | |
|    |          |  | | | |                                        /--- Daohugoucossus parallelivenius(55)
|    |          |  | | | \--------------------61---------------------+
|    |          |  | | |                                          \--- Daohugoucossus lii(56)
|    |          |  | | |
|    |          |  | | |                                          /--- Palaeontinodes reshuitangensis(37)
|    |          |  | | |                                       /57+
|    |          \6-+  | |                                        | \--- Palaeontinodes cf shabarovi(39)
|    |            | | \--------------------41---------------------+
|    |            | |                                          \------ Daohugoucossus solutus(54)
|    |            | |
|    |            | \--------------------------------------------------- Daohugoucossus shii(53)
|    |            |
|    |            |                                               /--- Palaeontinodes separatus(36)
|    |            \-------------------------34-------------------------+
|    |                                                            \--- Palaeontinodes daohugouensis(38)
|    |
|    \----------------------------------------------------------------------- Dunstania petrophila(6)
|
\---------------------------------------------------------------------------- Prosbolomorpha clara(5)
```

Consensus tree logs (Parsimony analyses) on Phylogenic Matrix 1 (*Austroprosboloides vandijki* and *Australiastroprosbole maculata* were set as outgroup)

Bootstrap method with heuristic search:

  Number of bootstrap replicates = 1000

  Starting seed = generated automatically

  Number of characters resampled in each replicate = 119

  Optimality criterion = parsimony

    Character-status summary:

      Of 119 total characters:

        All characters are of type 'unord'

        All characters have equal weight

        10 characters are parsimony-uninformative

        Number of parsimony-informative characters = 109

  Starting tree(s) obtained via stepwise addition

    Addition sequence: random

    Number of replicates = 100

    Starting seed = generated automatically

    Number of trees held at each step = 5

  Branch-swapping algorithm: tree-bisection-reconnection (TBR) with reconnection limit = 8

    Steepest descent option not in effect

  Initial 'Maxtrees' setting = 100 (will be auto-increased by 100)

  Branches collapsed (creating polytomies) if maximum branch length is zero

  'MulTrees' option not in effect; only 1 tree will be saved per replicate

  No topological constraints in effect

  Trees are unrooted

Bootstrap 50% majority-rule consensus tree on Phylogenic Matrix 1 (*Austroprosboloides vandijki* and *Australiastroprosbole maculata* were set as outgroup)

```
/-------------------------------------------------------------------------------------------------------------- Austroprosboloides vandijki(1)
|
+-------------------------------------------------------------------------------------------------------------- Australiastroprosbole maculata(2)
|
|      /------------------------------------------------------------------------------------------------------- Gallodunstania grauvogeli(3)
|      |
|      |      /------------------------------------------------------------------------------------------------ Fletcheriana triassica(4)
|      |      |
|      |      |                                                            /--------- Papiliontina dracomima(7)
|      |      |                                                            |
|      |      |                                                         /91-+    /---- Papiliontina machaon(8)
|      |      |                                                         |   \-41-+
```

```
|     |     |  /---------------------------------------31--------------------------------------+       \---- Papiliontina spectans(9)
|     |     |  |                                                                  |
|     |     |  |                                                                  |       /---- Karoontina magna(10)
|     |     |  |                                                                  \---71---+
|     |     |  |                                                                           \---- Hallakkungis amisanus(11)
|     |     |  |
|     |     |  |                                                                           /---- Plachutella exculpta(12)
|     |     |  |                                                                      /-1--+
|     |     |  |                                                                      |    \---- Plachutella rotundata(19)
|     |     |  |                                                                  /66-+
|     |     |  |                                                                  |   |    /---- Plachutella derupta(20)
|     |     |  |                                                                  |   \-14-+
|     |     |  |                                                 /---------39----------+       \---- Plachutella magica(35)
|     |     |  |                                                 |                |
|     |     |  |                                                 |                |       /---- Suljuktaja turkestanensis(14)
|     |     |  |                                                 |                \---54---+
|     |     |  |                                                 |                         \---- Shurabocossus gigas(18)
|     |     |  |                                                 |
|     |     |  |                                                 |          /---------------------- Synapocossus sciacchitanoae(25)
|     |  /-98-+   |                                                |          |
|     |  |  |  |                                                 |          |  /------------------ Sinopalaeocossus trinervus(29)
|     |  |  |  |                                              /-2-+        /50-+   |
|     |  |  |  |                                              |  |        |  |  |           /---- Sinopalaeocossus longicaulis(30)
|     |  |  |  |                                              |  |        |  \50-+       /-49-+
|     |  |  |  |                                              |  |        |      |       |   \---- Sinopalaeocossus fangi(32)
|     |  |  |  |                                              |  |        |      |   /24-+
|     |  |  |  |                                              |  |  /-23-+      |   |  \--------- Sinopalaeocossus eumorphus(33)
|     |  |  |  |                                              |  |  |   |      \-43-+
|     |  |  |  |                                              |  |  |   |          |       /---- Sinopalaeocossus laevis(31)
|     |  |  |  |                                              |  |  |   |          \---34---+
|     |  |  |  |                                              |  \11-+   |                   \---- Sinopalaeocossus amoenus(34)
|     |  |  |  |                                              |      |   |
|     |  |  |  |                                              |      |   \-------------------------- Neimenggucossus normalis(42)
|     |  |  |  |                                              |      |
|     |  |  |  |                                          /-2--+      \------------------------------- Kallicossus ningchengensis(47)
|     |  |  |  |                                          |   |
|     |  |  |  |                                          |   |                            /---- Palaeocossus jurassicus(17)
|     |  |  |  |                                          |   |                        /-42-+
|     |  |  |  |                                          |   |                        |   \---- Martynovocossus bellus(46)
|     |  |  |  |                                          |   |                    /-1-+
|     |  |  |  |                                          |   |                    |   |   /---- Martynovocossus punctulosus(43)
|     |  |  |  |                                          |   |                    |   \-1--+
|     |  |  |  |                                          |   |                /-6--+        \---- Martynovocossus cheni(45)
|  /-87-+  |   |  |                                          |   |                |   |
|  |   |  |   \98-+                                        /-3-+   |               /13-+   \------------- Martynovocossus decorus(44)
|  |   |  |      |                                       |  |   |               |  |
|  |   |  |      |                                       |  |   |               |  \------------------ Martynovocossus turgaiensis(22)
|  |   |  |      |                                       |  |   \-------1--------+
|  |   |  |      |                                       |  |                   |             /---- Suljuktocossus yinae(26)
|  |   |  |      |                                       |  |                   |         /-6--+
|  |   |  |      |                                       |  |                   |         |   \---- Suljuktocossus chifengensis(28)
|  |   |  |      |                                       |  |                   \-----0------+
|  |   |  |      |                                   /-4--+  |                               \--------- Suljuktocossus coloratus(27)
|  |   |  |      |                                   |   |  |
|  |   |  |      |                                   |   |  \------------------------------------------- Palaeontina oolitica(24)
|  |   |  |      |                                   |   |
|  |   |  |      |                                   |   |                 /---------------------- Phragmatoecites damesi(15)
|  |   |  |      |                                   |   |                 |
|  |   |  |      |                                   |   |                 |             /---- Ningchengia minuta(40)
|  |   |  |      |                                   |   \-----------21------------+   /-----66------+
|  |   |  |      |                                   |                   |  |            \---- Ningchengia aspera(41)
|  |   |  |      |  /----------------12----------------+                       |  |
|  |   |  |      |  |                                |                   \31-+       /--------- Eoiocossus validus(50)
|  |   |  |      |  |                                |                      |       |
|  |   |  |      |  |                                |                      |   /90-+   /---- Eoiocossus giganteus(51)
|  |   |  |      |  |                                |                      |   |  \-50-+
|  |   |  |      |  |                                |                      \-58-+       \---- Eoiocossus conchatus(52)
|  |   |  |      |  |                                |                          |
|  |   |  |      |  |                                |                          \------------- Abrocossus longus(58)
|  |   |  |      |  |                                |
```

```
 |  |   |  |        |  |                            \---------------------------------------------------- Suljuktocossus prosboloides(13)
 |  |   |  |        |  |
 |  |   |  |        |  |                                                                      /---- Palaeontinodes angarensis(16)
 |  |   |  |        |  |                                                                 /-30-+
 |  |   |  |        |  |                                                                 |    \---- Ijacossus suchanovae(21)
 |  |   |  |        |  |   /------------------------------------8------------------------------------+
 |  |   |  |        |  |   |                                                             \--------- Cladocossus undulatus(57)
 |  |   |  |        |  |   |
 |  |   |  |        |  |   |                                                             /--------- Gansucossus pectinatus(23)
 |  |   |  |        |  |   |                                                             |
 |  |   |  |        |  |   |               /------------------------28-------------------------+    /---- Gansucossus typicus(48)
 |  |   |  |        |  |   |               |                                             \-29-+
 |  |   |  |        |  |   |               |                                                  \---- Gansucossus luanpingensis(49)
 |  |   |  |        |  |   |               |
 |  |   |  |        |  |   |               |                                             /--------- Cicadomorpha guancaishanensis(59)
 |  |   |  |        |  |   |               |                                             |
 |  |   |  |        |  |   |               |                                          /15-+    /---- Cicadomorpha parula(63)
 |  |   |  |        |  |   |               |                                          |  \-49-+
 |  |   |  |        |  |   |               |  /--------------------30---------------------+        \---- Cicadomorpha milva(64)
 |  |   |  |       \49-+   |                  |  |                                       |
 |  |   |  |           |   |             /-25-+  |                                       |       /---- Cicadomorpha sp1(66)
 |  |   |  |           |   |             |  |  |                                          \---49---+
 |  |   |  |           |   |             |  |  |                                                  \---- Cicadomorpha sp2(69)
 |  |   |  |           |   |             |  |  |
 |  |  \56-+           |   |             |  |  |  /---------------------------------------------------- Talbragarocossus jurassicus(60)
 |  |      |           |   |             |  |  |  |
 |  |      |           |   |             |  |  |  |                                           /---- Prolystra lithographica(61)
 |  |      |           |   |             |  |  |  |                                      /-37-+
 |  |      |           |   |             |  |  |  |                                      |   \---- Eocicada microcephala(62)
 |  |      |           |   |             |  |  |  |      /----------------48----------------+
 |  |      |           |   |             |  \15-+  |      |                             \--------- Archipsyche eichstattensis(65)
 |  |      |           |   |             |      |  |      |
 |  |      |           |   |             |      |  |      |                              /--------- Ilerdocossus prowsei(68)
 |  |      |           |   |             |      |  |      |                              |
 |  |      |           |   |             |      |  |      |                            /39-+    /---- Ilerdocossus pulcherrima(76)
 |  |      |           |   |             |      |  |      |                            |  \-46-+
\95-+      |           |   |             |      |  |      |                         /-2--+       \---- Ilerdocossus fengningensis(79)
    |      |           |   |             |      |  |      |                         |  |
    |      |           |   |             |      |  |      |                         |  |      /---- Pachypsyche vidali(73)
    |      |           |   |             |      |  |      |                     /-2-+  \---12---+
    |      |           |   |             |      |  |      |                     |  |          \---- Ilerdocossus villaltai(75)
    |      |           |   |             |      |  |   /35-+                    |  |
    |      |           |   |             |      \72-+   |  |                    |  \------------------ Ilerdocossus hui(78)
    |      |           |   |             |          |   |  |                    |
    |      |           |   |             |          |   |  |                    |             /---- Yanocossus guoi(71)
    |      |           |   |             |          |   |  |  /-------3--------+           /-79-+
    |      |          \-55-+             |          |   |  |  |                 |          |   \---- Miracossus ingentius(74)
    |      |               |             |          |   |  |  |                 |      /100+
    |      |               |             |          |   |  |  |                 |      |  \--------- Yanocossus cf guoi(72)
    |      |               |             |          |   |  |  |                 |  /-19-+
    |      |               |             |          |   |  |  |                 |  |   |      /---- Ilerdocossus exiguus(80)
    |      |               |          /15-+         |   |  |  |                 \-3-+   \---42---+
    |      |               |          |  |          |   |  |  |                     |          \---- Ilerdocossus beipiaoensis(82)
    |      |               |          |  |          |   |  \-14-+                    |
    |      |               |          |  |          |   |     |                     \------------------ Ilerdocossus dissidens(81)
    |      |               |          |  |          |   |     |
    |      |               |          |  |          |   |     |  /---------------------------------- Ilerdocossus ningchengensis(77)
    |      |               |          |  |          |   |     |  |
    |      |               |          |  |          |   |     |  |                             /---- Parawonnacottella penneyi(83)
    |      |               |          |  |          |   |     |  |                        /-27-+
    |      |               |          |  |          \-78-+     |  |                        |   \---- Baeocossus fortunatus(92)
    |      |               |          |  |               |     |  |  /----------7----------+
    |      |               |          |  |               |     \15-+  |                  \--------- Parawonnacottella araripensis(84)
    |      |               |          |  |               |        |  |
    |      |               |          |  |               |        |  |                   /--------- Cratocossus magnus(85)
    |      |               |          |  |               |        |  |                   |
    |      |               |          |  |               |        |  |                 /43-+    /---- Colossocossus loveridgei(87)
    |      |               |          |  |               |        \20-+                 |  \-23-+
    |      |               |          |  |               |            |         /-10-+       \---- Colossocossus giganticus(88)
    |      |               |          |  |               |            |         |   |
```

```
|     |          |    /-15-+  |          |          |     /-7-+   \------------- Colossocossus bechlyi(89)
|     |          |    |  |  |          |          |     |  |
|     |          |    |  |  |          |          |  /89-+  \------------------ Colossocossus rugosa(86)
|     |          |    |  |  |          |          |  |  |
|     |          |    |  |  |          |          \-12-+  \---------------------- Colossocossus acutus(90)
|     |          |    |  |  |          |             |
|     |          |    |  |  |          |             \-------------------------- Baeocossus muratai(91)
|     |          |    |  |  |          |
|     |          |    |  |  |          |                         /---- Valdicossus chesteri(67)
|     |          |    |  |  |          \--------------------49---------------------+
|     |          |    |  |  |                                    \---- Valdicossus mikewebsteri(70)
|     |          |  /10-+   |  |
|     |          |  |  |  |  |                                    /---- Daohugoucossus parallelivenius(55)
|     |          |  |  |  |  \-----------------------------61------------------------------+
|     |          |  |  |  |                                       \---- Daohugoucossus lii(56)
|     |          |  |  |  |
|     |          |  |  |  |                                       /---- Palaeontinodes reshuitangensis(37)
|     |          |  |  |  |                                    /-56-+
|     |          \-6-+  |  |                                    |   \---- Palaeontinodes cf shabarovi(39)
|     |             |  |  \-----------------------------38-----------------------------+
|     |             |  |                                        \--------- Daohugoucossus solutus(54)
|     |             |  |
|     |             |  \------------------------------------------------------------------------ Daohugoucossus shii(53)
|     |             |
|     |             |                                             /---- Palaeontinodes separatus(36)
|     |             \-------------------------------------33-------------------------------------+
|     |                                                           \---- Palaeontinodes daohugouensis(38)
|     |
|     \---------------------------------------------------------------------------------------------------- Dunstania petrophila(6)
|
\------------------------------------------------------------------------------------------------------------ Prosbolomorpha clara(5)
```

Consensus tree logs (Parsimony analyses) on Phylogenic Matrix 2 (*Austroprosboloides vandijki* was set as outgroup)

Bootstrap method with heuristic search:

Number of bootstrap replicates = 1000

Starting seed = generated automatically

Number of characters resampled in each replicate = 119

Optimality criterion = parsimony

Character-status summary:

Of 119 total characters:

All characters are of type 'unord'

All characters have equal weight

4 characters are constant (proportion = 0.0336134)

11 variable characters are parsimony-uninformative

Number of parsimony-informative characters = 104

Starting tree(s) obtained via stepwise addition

Addition sequence: random

Number of replicates = 100

Starting seed = generated automatically

Number of trees held at each step = 5

Branch-swapping algorithm: tree-bisection-reconnection (TBR) with reconnection limit = 8

Steepest descent option not in effect

Initial 'Maxtrees' setting = 400 (will be auto-increased by 100)

Branches collapsed (creating polytomies) if maximum branch length is zero

'MulTrees' option not in effect; only 1 tree will be saved per replicate

No topological constraints in effect

Trees are unrooted

Bootstrap 50% majority-rule consensus tree on Phylogenic Matrix 2 (*Austroprosboloides vandijki* was set as outgroup)

```
/------------------------------------------------------------------------------------------------------------------------ Austroprosboloides vandijki(1)
|
|  /-------------------------------------------------------------------------------------------------------------------- Australiastroprosbole maculata(2)
|  |
|  |     /---------------------------------------------------------------------------------------------------------- Gallodunstania grauvogeli(3)
|  |     |
|  |     |     /------------------------------------------------------------------------------------------------ Fletcheriana triassica(4)
|  |     |     |
|  |     |     |                                                             /--------- Papiliontina dracomima(7)
|  |     |     |                                                             |
|  |     |     |                                                         /-97-+   /----- Papiliontina machaon(8)
|  |     |     |                                                         |    \45-+
|  |     |     |  /-------------------------------------37--------------------------------------+       \----- Papiliontina spectans(9)
|  |     |     |  |                                                      |
|  |     |     |  |                                                      |       /----- Karoontina magna(10)
|  |     |     |  |                                                      \---71---+
|  |     |     |  |                                                               \----- Hallakkungis amisanus(11)
|  |     |     |  |
|  |     |     |  |                               /-------------------------------------------- Suljuktocossus prosboloides(12)
|  |     |     |  |                               |
|  |     |     |  |                               |                                  /----- Palaeocossus jurassicus(15)
|  |     |     |  |                               |                              /45-+
|  |     |     |  |                               |                              |  \----- Martynovocossus bellus(36)
|  |     |  /-99-+  |                               |                          /-1--+
|  |     |  |  |  |                               |                       |  |  /----- Martynovocossus punctulosus(33)
|  |     |  |  |  |                               |                       |  \-1-+
|  |     |  |  |  |                               |            /-----11-----+       \----- Martynovocossus cheni(35)
|  |     |  |  |  |                               |            |          |
|  |     |  |  |  |                               |            |          \------------- Martynovocossus decorus(34)
|  |     |  |  |  |                               |        /-2--+
|  |     |  |  |  |                               |        |   |  /---------------------- Martynovocossus turgaiensis(17)
|  |     |  |  |  |                               |        |   |  |
|  |     |  |  |  |                            /-8-+         |   \25-+   /----------------- Synapocossus sciacchitanoae(19)
|  |     |  |  |  |                            |  |          |      |   |
|  |     |  |  |  |                            |  |          |      \-61-+   /------------- Sinopalaeocossus trinervus(23)
|  |     |  |  |  |                            |  |          |          |  |
|  |     |  |  |  |                            |  |          |          |  |      /----- Sinopalaeocossus longicaulis(24)
|  |     |  |  |  |                            |  |       /-4-+           \51-+   /67-+
|  |     |  |  |  |                            |  |       |  |              |  |  \----- Sinopalaeocossus fangi(26)
```

```
|  |     |  |   |  |                           |  |      |  |          \-45-+
|  |     |  |   \99-+                          |  |      |  |              |  /----- Sinopalaeococcus laevis(25)
|  |     |  |      |                           |  |      |  |            \49-+
|  |     |  |      |                           |  |   /-10-+  |                \----- Sinopalaeococcus amoenus(27)
|  |     |  |      |  /-------------------24-------------------+  |   |   |  |
|  |     |  |      |  |                        |  |   |   |  |              /----- Suljuktococcus yinae(20)
|  |  /-87-+  |       |   |                    |  |   |  |  \------------7-------------+
|  |  |  |  |      |  |                        |  \-12-+   |                \----- Suljuktococcus chifengensis(22)
|  |  |  |  |      |  |                        |      |   |
|  |  |  |  |      |  |                        |      |   \------------------------------------ Suljuktococcus coloratus(21)
|  |  |  |  |      |  |                        |      |
\----+  |   |  |       |   |                   |      \---------------------------------------- Palaeontina oolitica(18)
   |  |  |  |      |  |                        |
   |  |  |  |      |  |                        |                 /----------------------- Phragmatoecites damesi(13)
   |  |  |  |      |  |                        |                 |
   |  |  |  |      |  |                        \------------36------------+    /------------------ Ningchengia aspera(32)
   |  |  |  |      |  |                                          |   |
   |  |  |  |      |  |                                          |   |       /--------- Eoiocossus validus(37)
   |  |  |  |      |  |                                          \-52-+       |
   |  |  |  |      |  |                                              |  /-94-+   /----- Eoiocossus giganteus(38)
   |  |  |  |      |  |                                              |  |   \52-+
   |  |  |  |      |  |                                              \65-+       \----- Eoiocossus conchatus(39)
   |  |  |  |      |  |                                                 |
   |  |  |  |      |  |                                                 \-------------- Abrocossus longus(44)
   |  |  |  |      |  |
   |  |  |  |      |  |                                                         /----- Palaeontinodes angarensis(14)
   |  |  |  |      |  |  /---------------------------------------30---------------------------------------+
   |  |  |  |      |  |  |                                                      \----- Ijacossus suchanovae(16)
   |  |  |  |      \-62-+  |
   |  |  |  |          |  |                                                     /----- Palaeontinodes separatus(28)
   |  |  |  |          |  |     /----------------------------------36-----------------------------------+
   |  |  |  |          |  |     |                                               \----- Palaeontinodes daohugouensis(30)
   |  |  |  |          |  |     |
   |  |  |  |          |  |     |                                               /----- Palaeontinodes reshuitangensis(29)
   |  |  |  |          |  |     |     /------------------------------84-----------------------------+
   |  |  |  |          |  |     |     |                                         \----- Palaeontinodes cf shabarovi(31)
   |  |  |  |          |  |     |     |
   |  |  |  |          |  |     |     |                                         /----- Daohugoucossus parallelivenius(41)
   |  |  |  |          |  |     |     |  /----------------------------60----------------------------+
   |  |  |  |          |  |     |     |  |                                      \----- Daohugoucossus lii(42)
   |  |  |  |          |  |     |     |  |
   |  |  |  |          |  |     |  /-24-+   |                                   /--------- Cicadomorpha guancaishanensis(45)
   |  |  |  |          |  |     |  |   |  |                                     |
   |  |  |  |          |  |     |  |   |  |                                 /-19-+   /----- Cicadomorpha parula(49)
   |  |  |  |          |  |     |  |   |  |                                 |   \59-+
   |  |  |  |          |  |     |  |   |  |   /---------------------46---------------------+       \----- Cicadomorpha milva(50)
   |  |  \53-+          |  |       |  |   |  |                               |
   |  |     |          |  |     |  |   \40-+   |                             |      /----- Cicadomorpha sp1(52)
   |  |     |          |  |     |  |      |  |                               \---53---+
   |  |     |          |  |     |  |      |  |                                      \----- Cicadomorpha sp2(54)
   |  |     |          |  |     |  |      |  |
   |  |     |          |  |  /-9--+  |      |  |  /------------------------------------------------------ Talbragarocossus jurassicus(46)
   |  |     |          |  |  |  |  |      |  |  |
   |  |     |          \68-+   |  |  |      |  |  |                                 /----- Prolystra lithographica(47)
   |  |     |             |  |  |  |      |  |  |                               /37-+
   |  |     |             |  |  |  |      |  |  |                               |  \----- Eocicada microcephala(48)
   |  |     |             |  |  |  |      \-70-+  |      /-----------------49-----------------+
   |  |     |             |  |  |  |          |  |      |                       \--------- Archipsyche eichstattensis(51)
   \96-+      |             |  |  |  |          |  |      |
      |     |             |  |  |  |          |  |      |                       /--------- Ilerdocossus prowsei(53)
      |     |             |  |  |  |          |  |      |                       |
      |     |             |  |  |  |          |  |      |                   /-40-+   /----- Ilerdocossus pulcherrima(60)
      |     |             |  |  |  |          |  |      |                   |   \44-+
      |     |             |  |  |  |          |  |      |                /-3-+       \----- Ilerdocossus fengningensis(63)
      |     |             |  |  |  |          |  |      |                |  |
      |     |             |  |  |  |          |  |      |                |  |      /----- Pachypsyche vidali(57)
      |     |             |  |  |  |          |  |   /16-+              /-2--+   \---12---+
      |     |             |  |  |  |          \87-+    |  |             |   |          \----- Ilerdocossus villaltai(59)
      |     |             |  |  |  |             |   |  |              |   |
      |     |             |  |  |  |             |   |  |          /-3-+   \------------------ Ilerdocossus hui(62)
```

```
 |     |          |  |  |  |          |  |  |           |  |
 |     |          |  |  |  |          |  |  |           |  |           /----- Ilerdocossus exiguus(64)
 |     |          |  |  |  |          |  |  |  /------4------+  \-------45--------+
 |     |          |  |  \17-+          |  |  |  |         |              \----- Ilerdocossus beipiaoensis(66)
 |     |          |  |     |          |  |  |  |         |
 |     |          |  |     |          |  |  |  |         \--------------------------- Ilerdocossus dissidens(65)
 |     |          |  |     |          |  |  |  |
 |     |          |  |     |          |  |  \-24-+   /------------------------------------ Ilerdocossus ningchengensis(61)
 |     |          |  |     |          |  |     |  |
 |     |          \-10-+     |          |  |     |  |                   /----- Parawonnacottella penneyi(67)
 |     |             |     |          |  |     |  |              /29-+
 |     |             |     |          |  |     |  |              |  \----- Baeocossus fortunatus(76)
 |     |             |     |          |  |     |  |  /----------11----------+
 |     |             |     |          |  |     \-23-+  |                \--------- Parawonnacottella araripensis(68)
 |     |             |     |          \-91-+        |  |
 |     |             |     |             |        |  |              /--------- Cratocossus magnus(69)
 |     |             |     |             |        |  |              |
 |     |             |     |             |        |  |          /-44-+   /----- Colossocossus loveridgei(71)
 |     |             |     |             |        \30-+           |   \24-+
 |     |             |     |             |           |        /-9-+      \----- Colossocossus giganticus(72)
 |     |             |     |             |           |        |  |
 |     |             |     |             |           |     /-7--+  \-------------- Colossocossus bechlyi(73)
 |     |             |     |             |           |     |  |
 |     |             |     |             |           |  /89-+   \------------------ Colossocossus rugosa(70)
 |     |             |     |             |           |  |  |
 |     |             |     |             |           \-13-+  \----------------------- Colossocossus acutus(74)
 |     |             |     |             |              |
 |     |             |     |             |              \--------------------------- Baeocossus muratai(75)
 |     |             |     |             |
 |     |             |     |             |                            /----- Yanocossus guoi(55)
 |     |             |     |             |                        /79-+
 |     |             |     |             |                        |  \----- Miracossus ingentius(58)
 |     |             |     |             \------------------100-------------------+
 |     |             |     |                                      \--------- Yanocossus cf guoi(56)
 |     |             |     |
 |     |             |     \---------------------------------------------------------------------- Daohugoucossus shii(40)
 |     |             |
 |     |             \------------------------------------------------------------------------------ Cladocossus undulatus(43)
 |     |
 |     \---------------------------------------------------------------------------------------------------- Dunstania petrophila(6)
 |
 \---------------------------------------------------------------------------------------------------------------- Prosbolomorpha clara(5)
```

Consensus tree logs (Parsimony analyses) on Phylogenic Matrix 3 (*Austroprosboloides vandijki* was set as outgroup)

Bootstrap method with heuristic search:

Number of bootstrap replicates = 1000

Starting seed = generated automatically

Number of characters resampled in each replicate = 77

Optimality criterion = parsimony

Character-status summary:

Of 77 total characters:

All characters are of type 'unord'

All characters have equal weight

6 characters are parsimony-uninformative

Number of parsimony-informative characters = 71

Starting tree(s) obtained via stepwise addition

Addition sequence: random

Number of replicates = 100

Starting seed = generated automatically

Number of trees held at each step = 5

Branch-swapping algorithm: tree-bisection-reconnection (TBR) with reconnection limit = 8

Steepest descent option not in effect

Initial 'Maxtrees' setting = 9500 (will be auto-increased by 100)

Branches collapsed (creating polytomies) if maximum branch length is zero

'MulTrees' option not in effect; only 1 tree will be saved per replicate

No topological constraints in effect

Trees are unrooted

Bootstrap 50% majority-rule consensus tree on Phylogenic Matrix 3 (*Austroprosboloides vandijki* was set as outgroup)

```
/---------------------------------------------------------------------------------------- Austroprosboloides vandijki(1)
|
|  /------------------------------------------------------------------------------------- Australiastroprosbole maculata(2)
|  |
|  |     /--------------------------------------------------------------------------------- Gallodunstania grauvogeli(3)
|  |     |
|  |     |     /--------------------------------------------------------------------------- Fletcheriana triassica(4)
|  |     |     |
|  |     |     |                                                  /-------- Papiliontina dracomima(7)
|  |     |     |                                                  |
|  |     |     |  /----------------------------98----------------------------+   /---- Papiliontina machaon(8)
|  |     |     |  |                                               \48-+
|  |     |     |  |                                                   \---- Papiliontina spectans(9)
|  |     |     |  |
|  |     |  /-99-+  |                                                     /---- Karoontina magna(10)
|  |     |  |  |  |  /----------------------------72----------------------------+
|  |     |  |  |  |  |                                                  \---- Hallakkungis amisanus(11)
|  |     |  |  |  |  |
|  |     |  |  |  |  |                          /------------------------------- Suljuktocossus prosboloides(12)
|  |     |  |  |  |  |                          |
|  |     |  |  |  |  |                          |                 /---- Palaeocossus jurassicus(15)
|  |     |  |  |  |  |                          |              /47-+
|  |     |  |  |  |  |                          |              |  \---- Martynovocossus bellus(36)
|  |     |  |  \99-+  |                          |              |
|  |     |  |     |  |                          |           /-0-+-------- Martynovocossus punctulosus(33)
|  |     |  |     |  |                          |           |  |
|  |     |  |     |  |                          |        /-5-+  \-------- Martynovocossus cheni(35)
|  |     |  |     |  |                          |        |  |
|  |     |  |     |  |                          |        |  \------------ Martynovocossus decorus(34)
|  |     |  |     |  |                          |    /---3----+
|  |     |  |     |  |                        /-3-+    |        |    /-------- Suljuktocossus yinae(20)
|  |     |  |     |  |                        |  |    |        |    |
```

```
| |    | |     | |                                          | |     |      \---0---+  /---- Suljuktocossus coloratus(21)
| |    | |     | |                                          | |     |           \-0-+
| |    | |     | |                                          | |  /-7-+                \---- Suljuktocossus chifengensis(22)
| |    | |     | |                                          | |  |  |
| |    | |     \42-+                                        | |  |  |  |  /--------------------- Martynovocossus turgaiensis(17)
| |    | |        |                                         | |  |  |  |  |
| |    | |        |                                         | |  |  |  |  |  /---------------- Synapocossus sciacchitanoae(19)
| |    | |        |                                         | |  |  \12-+  |
| |    | |        |                                         | |  |     |  |    /-------- Sinopalaeocossus trinervus(23)
| |    | |        |                                         | |  |     \-28-+    |
| |    | |        |  /-------------------------20-------------------------+  \-7-+        |  /-8-+  /---- Sinopalaeocossus laevis(25)
| |  /88-+  |      | |                                      |     |         | |  \56-+
| | |  | |        | |                                       |     |        \67-+    \---- Sinopalaeocossus amoenus(27)
| | |  | |        | |                                       |     |          |
| | |  | |        | |                                       |     |          |    /---- Sinopalaeocossus longicaulis(24)
| | |  | |        | |                                       |     |          \--65---+
| | |  | |        | |                                       |     |               \---- Sinopalaeocossus fangi(26)
\---+  |  |  |        | |                                   |     |
   | | | |        | |                                       |     \----------------------------- Palaeontina oolitica(18)
   | | | |        | |                                       |
   | | | |        | |                                       |          /--------------------- Phragmatoecites damesi(13)
   | | | |        | |                                       |          |
   | | | |        | |                                       \------46-------+   /---------------- Ningchengia aspera(32)
   | | | |        | |                                                  |   |
   | | | |        | |                                                  |   |    /-------- Eoiocossus validus(37)
   | | | |        | |                                                  \-65-+    |
   | | | |        \63-+                                                   |  /94-+  /---- Eoiocossus giganteus(38)
   | | | |           |                                                 |  |  \47-+
   | | | |           |                                                 \69-+     \---- Eoiocossus conchatus(39)
   | | | |           |                                                    |
   | | | |           |                                                    \------------ Abrocossus longus(44)
   | | | |           |
   | | | |           |                                                          /---- Palaeontinodes angarensis(14)
   | | | |           |  /--------------------------------------27---------------------------------------+
   | | | |           |  |                                                       \---- Ijacossus suchanovae(16)
   | | | |           |  |
   | | | |           |  |                                                       /---- Palaeontinodes separatus(28)
   | | | |           |  |  /-------------------------------------28-------------------------------------+
   | | | |           |  |  |                                                    \---- Palaeontinodes daohugouensis(30)
   | | | |           |  |  |
   | | | |           |  |  |                                                    /---- Palaeontinodes reshuitangensis(29)
   | | | |          \38-+  |  /-----------------------------------77-----------------------------------+
   | | | |              |  |  |                                                 \---- Palaeontinodes cf shabarovi(31)
   | | | |              |  |  |
   | | | |              |  |  |    /--------------------------------------------------------------------- Daohugoucossus shii(40)
   | | | |              |  |  |    |
   | | | |              |  |  |    |                                            /---- Daohugoucossus parallelivenius(41)
   | | | |              |  |  |    |  /-----------------------------61------------------------------+
   | | | |              |  |  |    |  |                                         \---- Daohugoucossus lii(42)
   | |  \55-+              \-6-+  |    |  |
   | |    |                  |  |  /15-+  |                                      /-------- Cicadomorpha guancaishanensis(45)
   | |    |                  |  |  |  |  |                                     |
   | |    |                  |  |  |  |  |                                   /16-+  /---- Cicadomorpha parula(49)
   | |    |                  |  |  |  |  |                                   |  \25-+
   | |    |                  |  |  |  |  |  /----------------------55------------------------+     \---- Cicadomorpha milva(50)
   | |    |                  |  |  |  |  |  |                                |
   | |    |                  |  |  |  \27-+  |                                |     /---- Cicadomorpha sp1(52)
   | |    |                  |  |  |     |  |                                \--54---+
   | |    |                  |  |  |     |  |                                       \---- Cicadomorpha sp2(54)
   | |    |                  |  |  |     |  |
   | |    |                  |  |  |     |  |  /--------------------------------------------------------- Talbragarocossus jurassicus(46)
   | |    |                  |  |  |     |  |  |
   | |    |                  \-1-+  |     |  |  |                                   /---- Prolystra lithographica(47)
   | |    |                     |  |     |  |  |                                 /40-+
   \96-+     |                     |  |     |  |  |                                 |  \---- Eocicada microcephala(48)
     |    |                     |  |     |  |  |   /------------------50------------------+
     |    |                     |  |     \50-+  |     |                          \-------- Archipsyche eichstattensis(51)
     |    |                     |  |        |  |     |
     |    |                     |  |        |  |     |                          /-------- Ilerdocossus prowsei(53)
     |    |                     |  |        |  |     |                          |
```

```
|    |                  | |       |  |    |                       /36-+  /---- Ilerdocossus pulcherrima(60)
|    |                  | |       |  |    |                       |  \47-+
|    |                  | |       |  |    |       /----------0-----------+     \---- Ilerdocossus fengningensis(63)
|    |                  | |       |  |    |       |               |
|    |                  | |       |  |    |       |               |     /---- Ilerdocossus exiguus(64)
|    |                  | |       |  |    |       |               \--47---+
|    |                  | |       |  |    |       |                     \---- Ilerdocossus beipiaoensis(66)
|    |                  | |       \-55-+     |        |
|    |                  | |          |    |       |        /------------------------ Pachypsyche vidali(57)
|    |                  | |          |  /13-+        |        |
|    |                  | |          |  |  |        |        |           /-------- Cratocossus magnus(69)
|    |                  | |          |  |  |     /-2-+         |           |
|    |                  \15-+           |  |  |     |  |        |          /47-+  /---- Colossocossus loveridgei(71)
|    |                    |          |  |  |     |  |     /23-+          |  \23-+
|    |                    |          |  |  |     |  |     |  |     /10-+     \---- Colossocossus giganticus(72)
|    |                    |          |  |  |     |  |     |  |     |  |
|    |                    |          |  |  |     |  |     |  |  /-7--+  \----------- Colossocossus bechlyi(73)
|    |                    |          |  |  |     |  |  /-3-+  |  |   |
|    |                    |          |  |  |     |  |  |  |  \90-+   \--------------- Colossocossus rugosa(70)
|    |                    |          |  |  |  /-7-+  |  |  |     |
|    |                    |          |  |  |  |  |  |  |  |     \-------------------- Colossocossus acutus(74)
|    |                    |          |  |  |  |  |  \-1-+  |
|    |                    |          |  |  |  |  |     |  \--------------------------- Ilerdocossus hui(62)
|    |                    |          |  |  |  |  |     |
|    |                    |          |  |  \28-+  |     \------------------------------ Ilerdocossus dissidens(65)
|    |                    |          \85-+     |  |
|    |                    |             |    |  \------------------------------------- Baeocossus muratai(75)
|    |                    |             |    |
|    |                    |             |    |                  /--------------- Ilerdocossus villaltai(59)
|    |                    |             |    |                  |
|    |                    |             |    \------------5-------------+  /----------- Ilerdocossus ningchengensis(61)
|    |                    |             |                       |  |
|    |                    |             |                       \-2-+     /---- Parawonnacottella penneyi(67)
|    |                    |             |                          |  /29-+
|    |                    |             |                          |  |  \---- Baeocossus fortunatus(76)
|    |                    |             |                          \-6-+
|    |                    |             |                             \-------- Parawonnacottella araripensis(68)
|    |                    |             |
|    |                    |             |                                /---- Yanocossus guoi(55)
|    |                    |             |                             /75-+
|    |                    |             |                             |  \---- Miracossus ingentius(58)
|    |                    |             \------------------100-------------------+
|    |                    |                                           \-------- Yanocossus cf guoi(56)
|    |                    |
|    |                    \------------------------------------------------------------------- Cladocossus undulatus(43)
|    |
|    \---------------------------------------------------------------------------------------------- Dunstania petrophila(6)
|
\------------------------------------------------------------------------------------------------------- Prosbolomorpha clara(5)
```

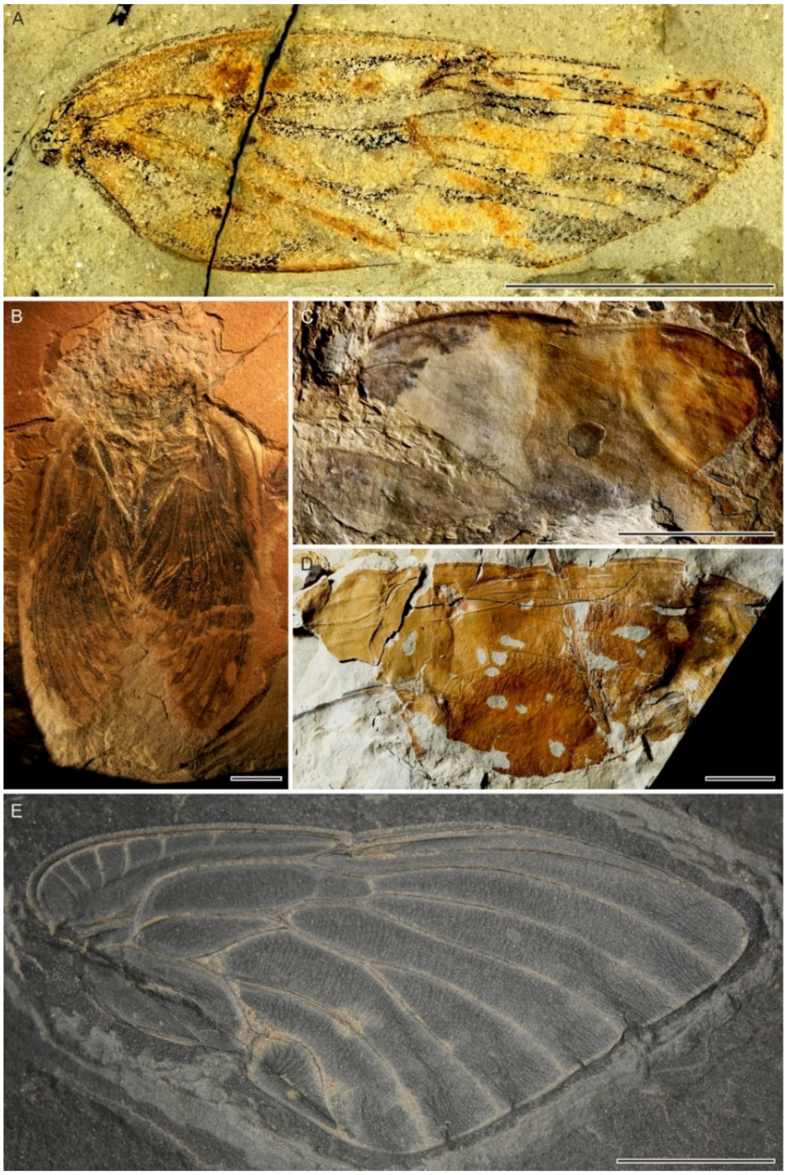

Fig. S1. Triassic representatives of Dunstaniidae and Triassic early Palaeontinidae. (**A**) *Gallodunstania grauvogeli*, from the Middle Triassic Grès à Voltzia Formation of France (No. 9152a). (**B**) *Fletcheriana triassica*, from the Upper Triassic of Australia (AM F.25234). (**C**) *Dunstania petrophila*, from the Upper Triassic Molteno Formation of South Africa (C-Dt II 781b). (**D)** *Karoontina magna*, from the Upper Triassic Molteno Formation of South Africa (C-Dt II 784b). (**E)** *Hallakkungis amisanus*, from the Upper Triassic Amisan Formation of South Korea (No. KNU-2009018a). Scale bars, 10 mm. All to scale. Photo credit: **A**, Arnold Staniczek, Staatlisches Museum für Naturkunde Stuttgart; **E**, Ying Wang, National Natural History Museum of China.

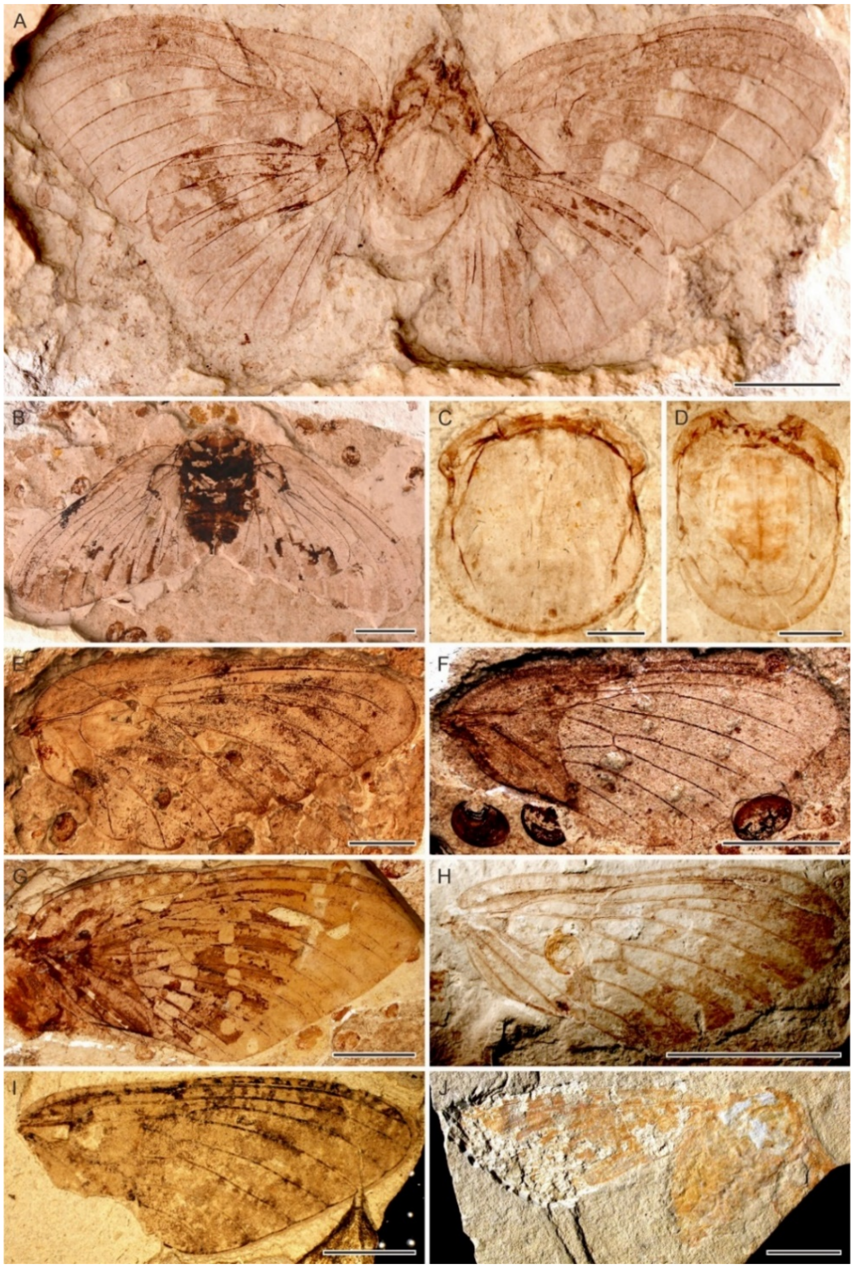

Fig. S2. Early Palaeontinidae representatives from the Middle Jurassic Daohugou Konservat-Lagerstätte, China (C–H), the Lower Cretaceous of Russia (I), and the Lower Cretaceous Yixian Formation, China (J). (**A**) *Sinopalaeocossus trinervus* (NIGP142082a). (**B**) *Sinopalaeocossus amoenus* (STMN48-1616a). (**C**–**D**) isolated mesonota. (**E**) *Eoiocossus validus* (NIGP142084). (**F**) *Suljuktocossus chifengensis* (NIGP143704). (**G**) *Martynovocossus punctulosus* (NIGP147872). (**H**) *Palaeontinodes daohugouensis* (NIGP143701a). (**I**) *Cicadomorpha* sp.1 (*92*). (**J**) *Cicadomorpha* sp. 2 (NIGP149229). Scale bars, 10 mm (**A**, **B** and **E**–**J**), and 5 mm (**C** and **D**).

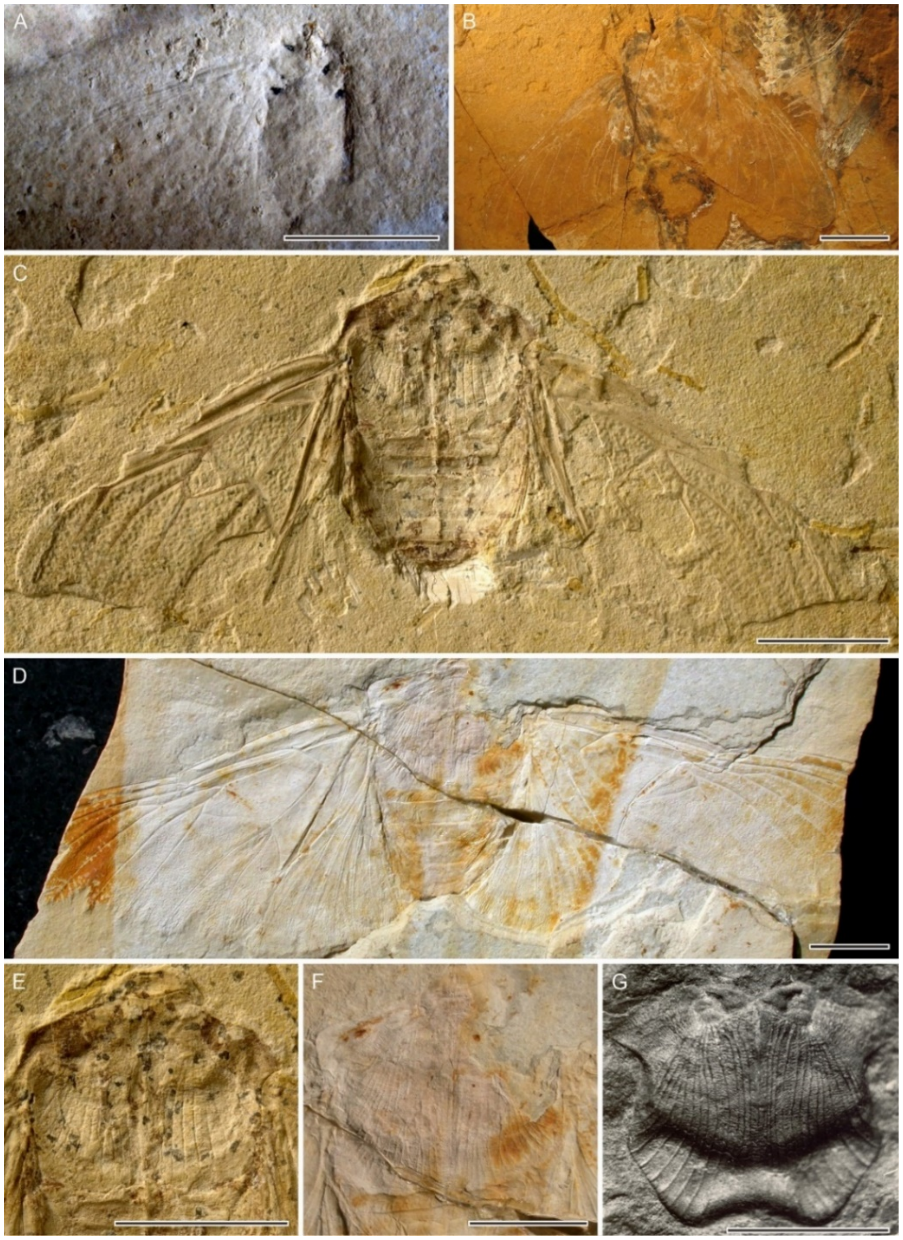

Fig. S3. Late Palaeontinidae representatives from Germany, Australia, Brazil, China, and UK. (**A**) *Prolystra lithographica*, from the Upper Jurassic Solnhofen Konservat-Lagerstätte of Germany (JME SOS1821). (**B**) *Talbragarocossus jurassicus*, from the Upper Jurassic Purlawaugh Formation of Australia (AM F.136849). (**C**) *Baeocossus finchae*, from the Lower Cretaceous Crato Formation of Brazil (SMNS 65501). (**D**) *Ierdocossus dissidens*, from the Lower Cretaceous Yixian Formation of China (NIGP154989). (**E**) Mesonotum of (**C**). (**F**) Mesonotum of (**D**). (**G**) Isolated mesonotum from the Lower Cretaceous Weald Clay Formation of England (NIGP204230). Scale bars, 10 mm. All to scale.

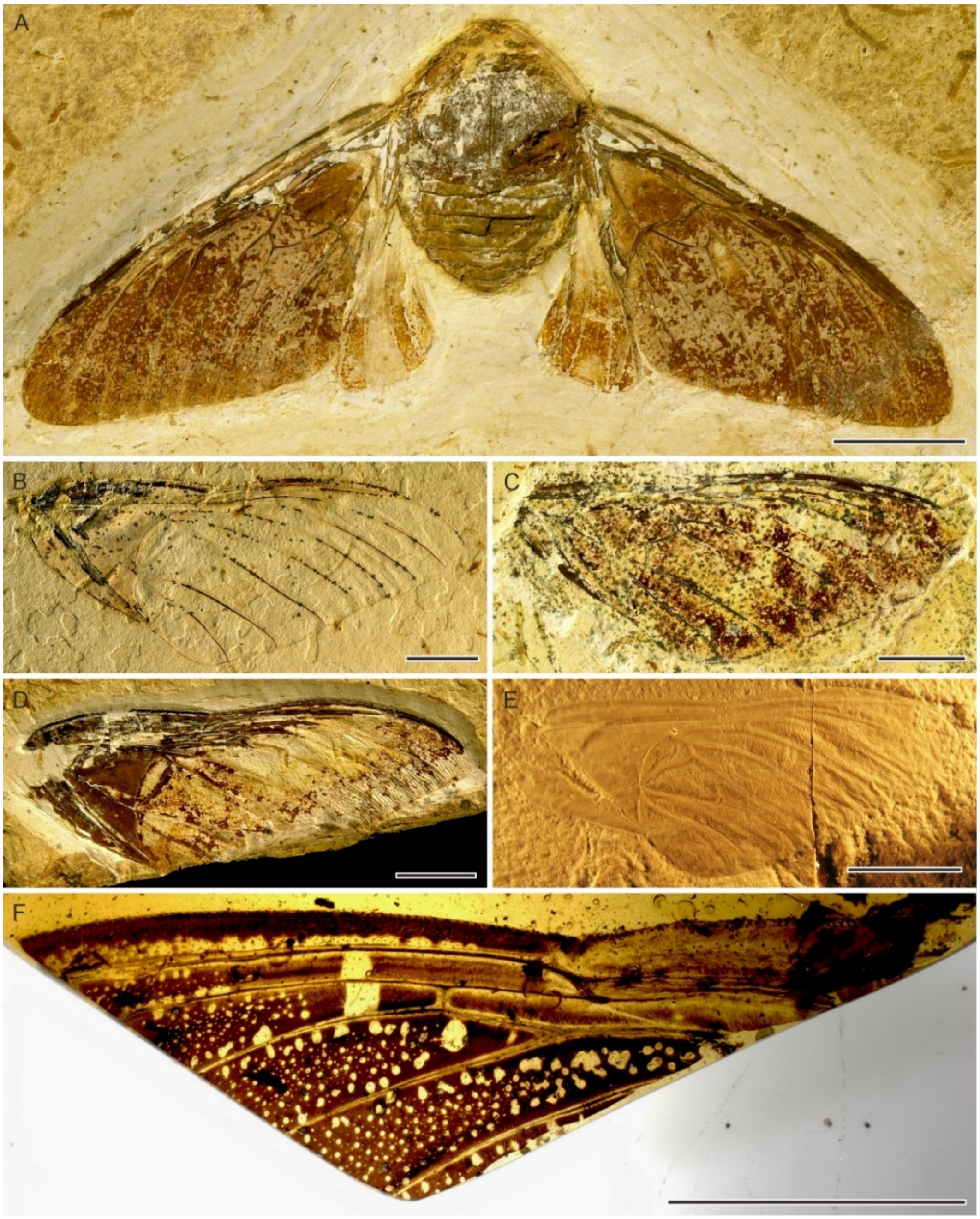

Fig. S4. Late Palaeontinidae representatives from the Lower Cretaceous Crato Formation of Brazil (A–D), the Lower Cretaceous La Pedrera de Rúbies Formation of Spain (E), and mid-Cretaceous Kachin amber of Myanmar (F). (**A**) *Baeocossus fortunatus* (SMNS 65547). (**B**) *Colossocossus bechlyi* (SMNS 65548). (**C**) *Parawonnacotella penneyi* (SMNS 65504). (**D**) *Colossocossus rugosa* (SMNS 65503). (**E**) *Ilerdocossus pulcherrima* (In 59486). (**F**) Palaeontinidae gen. et sp. indet (NIGP204231). Scale bars, 10 mm. All to scale.

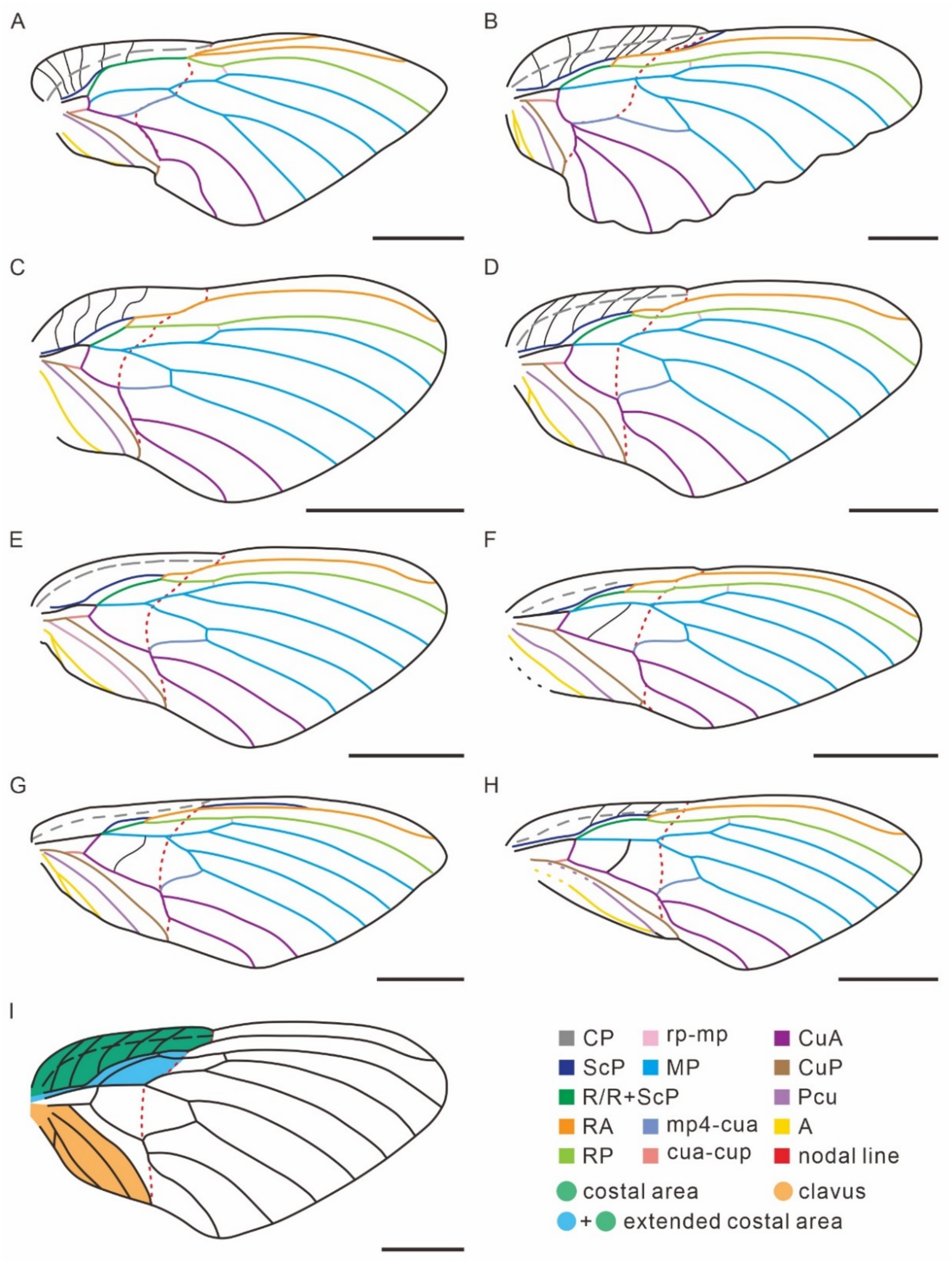

Fig. S5. Wing venation of representatives of forewings of early Palaeontinidae. (**A**) *Hallakkungis amisanus*. (**B**) *Eoiocossus validus*. (**C**) *Sinopalaeocossus trinervus*. (**D**) *Martynovocossus punctulosus*. (**E**) *Suljuktocossus chifengensis*. (**F**) *Palaeontinodes angarensis*. (**G**) *Daohugoucossus lii*. (**H**) *Cicadomorpha milva*. (**I**) Morphometric of costal area and clavus. Scale bars, 10 mm. All to scale.

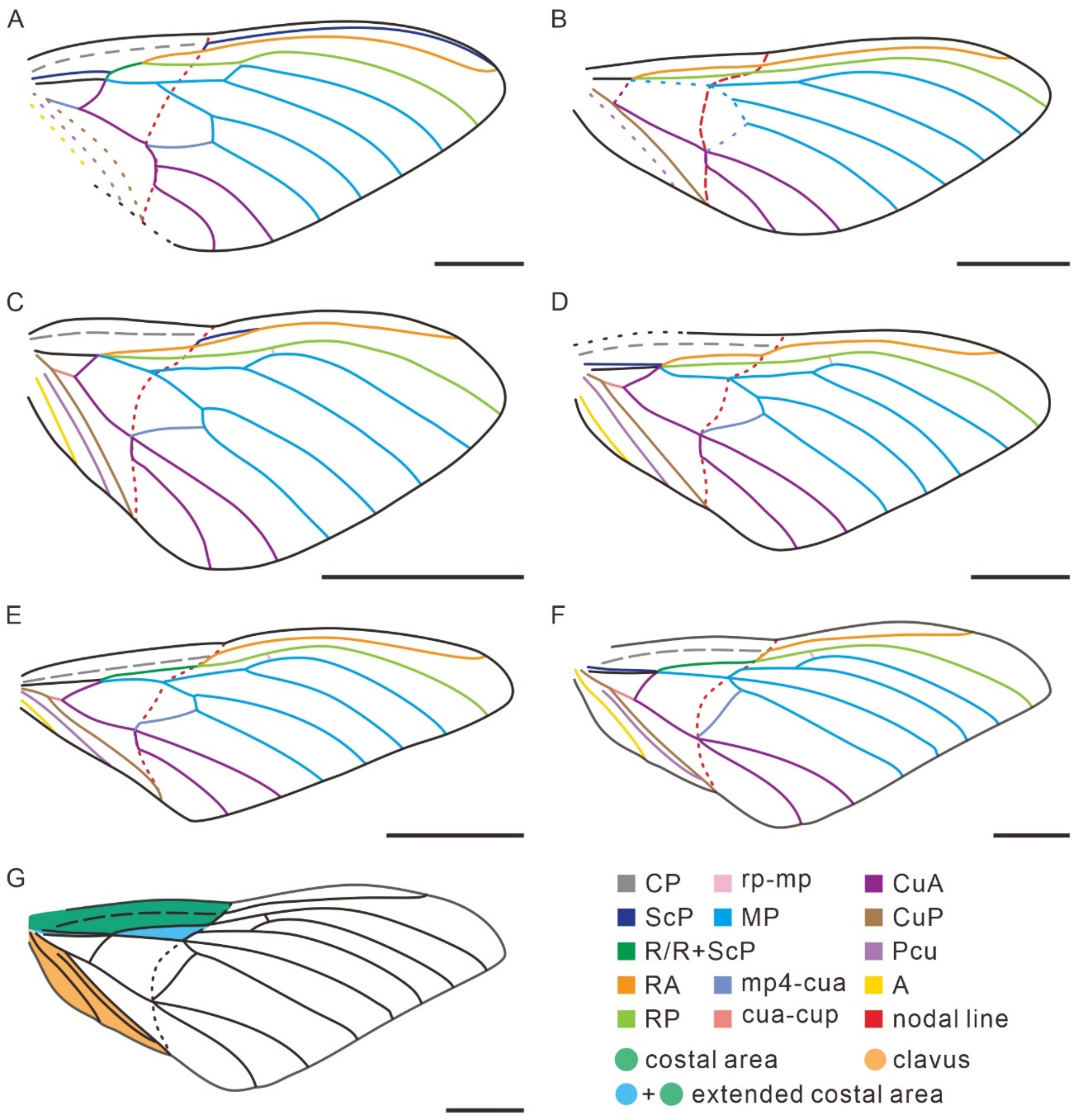

Fig. S6. Wing venation of representatives of forewings of late Palaeontinidae. (**A**) *Talbragarocossus jurassicus*. (**B**) *Prolystra lithographica*. (**C**) *Ilerdocossus beipiaoensis*. (**D**) *Parawonnacottella araripensis*. (**E**) *Baeocossus fortunatus*. (**F**) *Colossocossus bechlyi*. (**G**) Morphometric of costal area and clavus. Scale bars, 10 mm. All to scale.

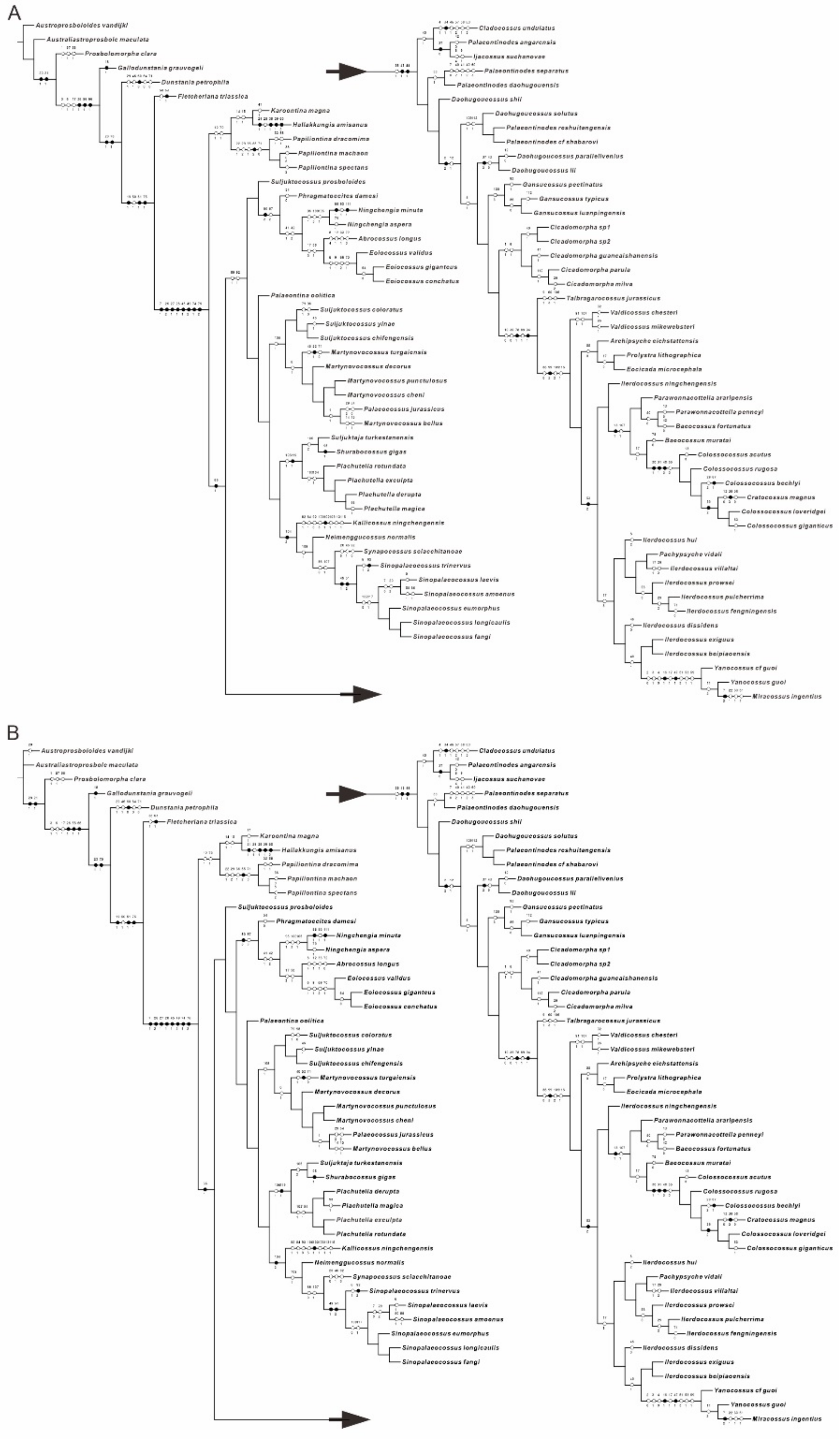

Fig. S7. 50% majority-rule consensus tree inferred from maximum parsimony analysis, based on Phylogenic Matrix 1, respectively with on one outgroup *Austroprosboloides vandijki* (A) or two outgroups *Austroprosboloides vandijki* and *Austroprosbole maculata* (B).

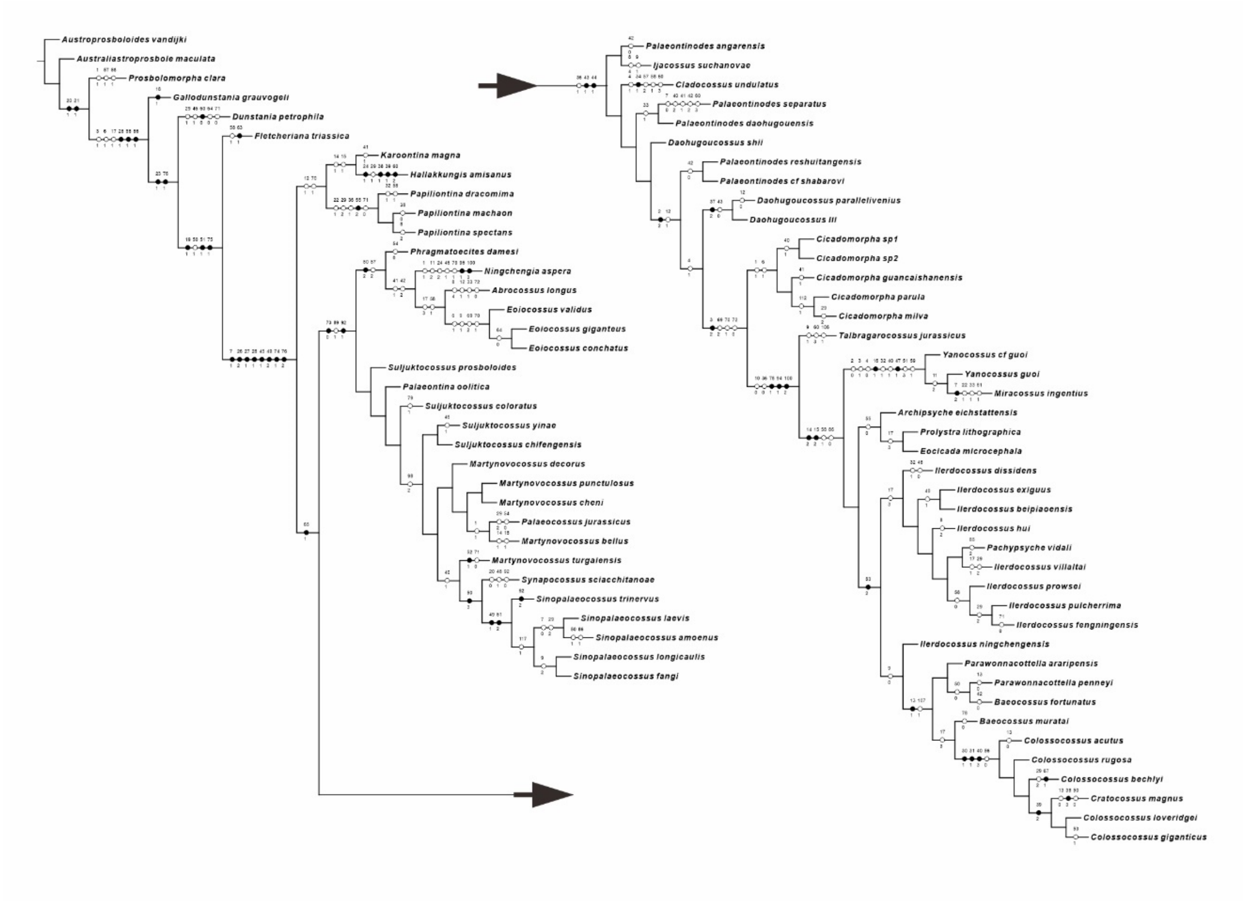

Fig. S8. 50% majority-rule consensus tree inferred from maximum parsimony analysis, based on Phylogenic Matrix 2.

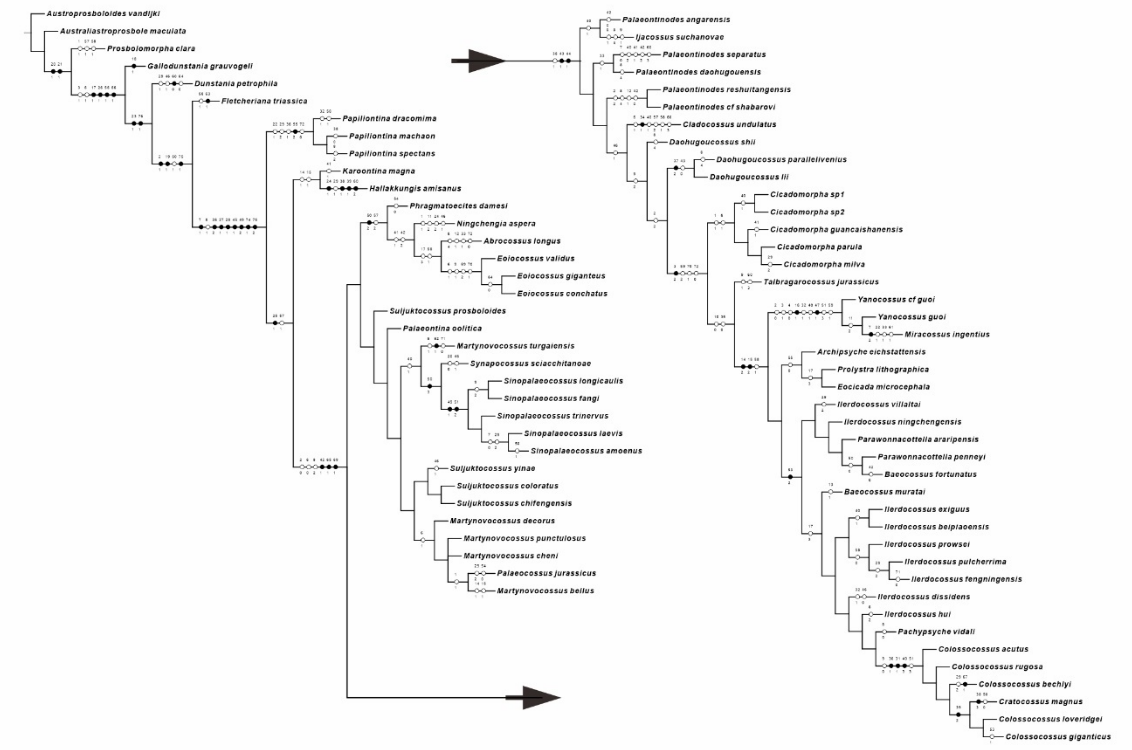

Fig. S9. 50% majority-rule consensus tree inferred from maximum parsimony analysis, based Phylogenic Matrix 3.

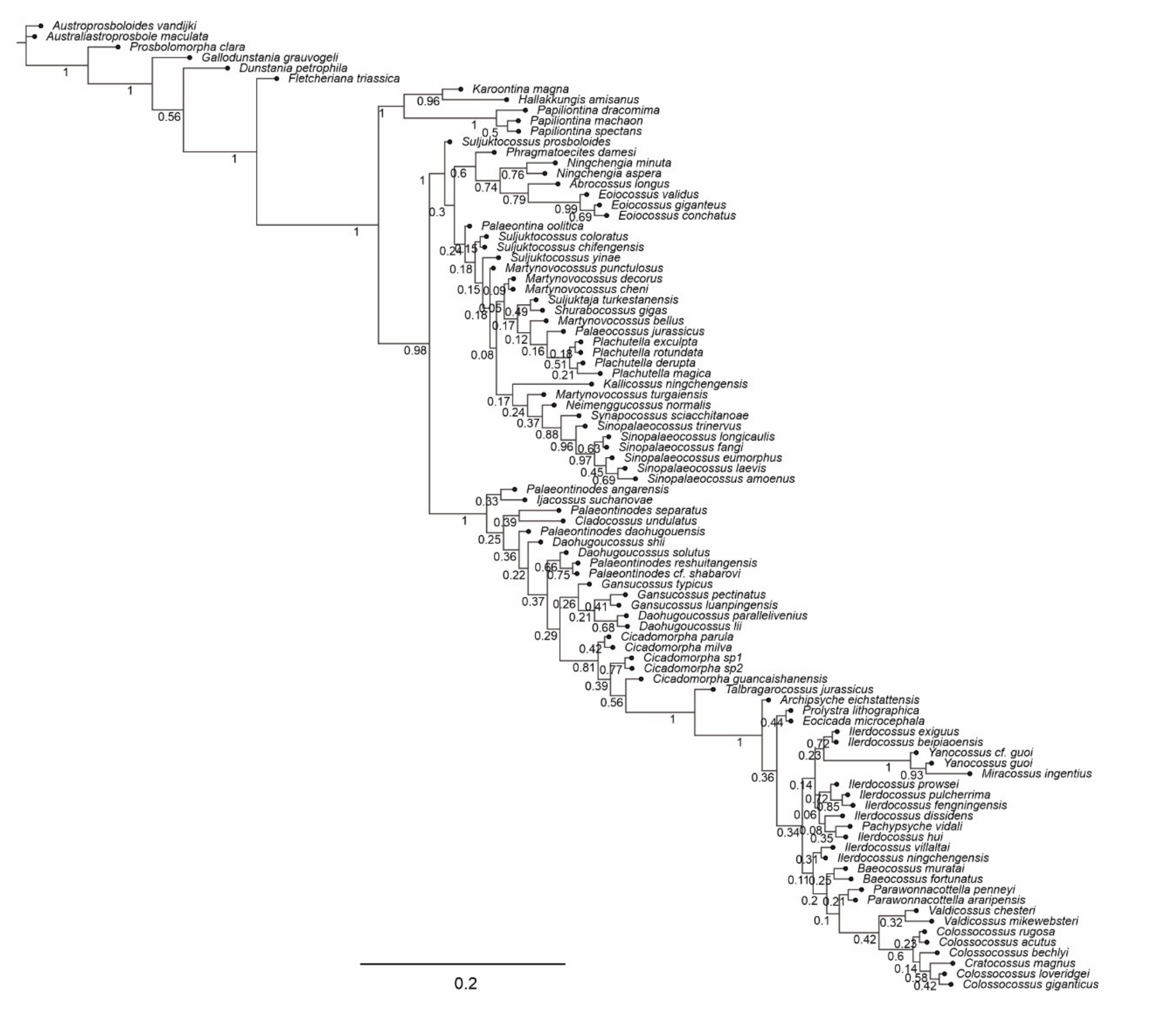

Fig. S10. Result of nonclock Bayesian analysis using Lewis Mkv model with gamma rate variation across characters (Mkv+Γ), based on Phylogenic Matrix 1.

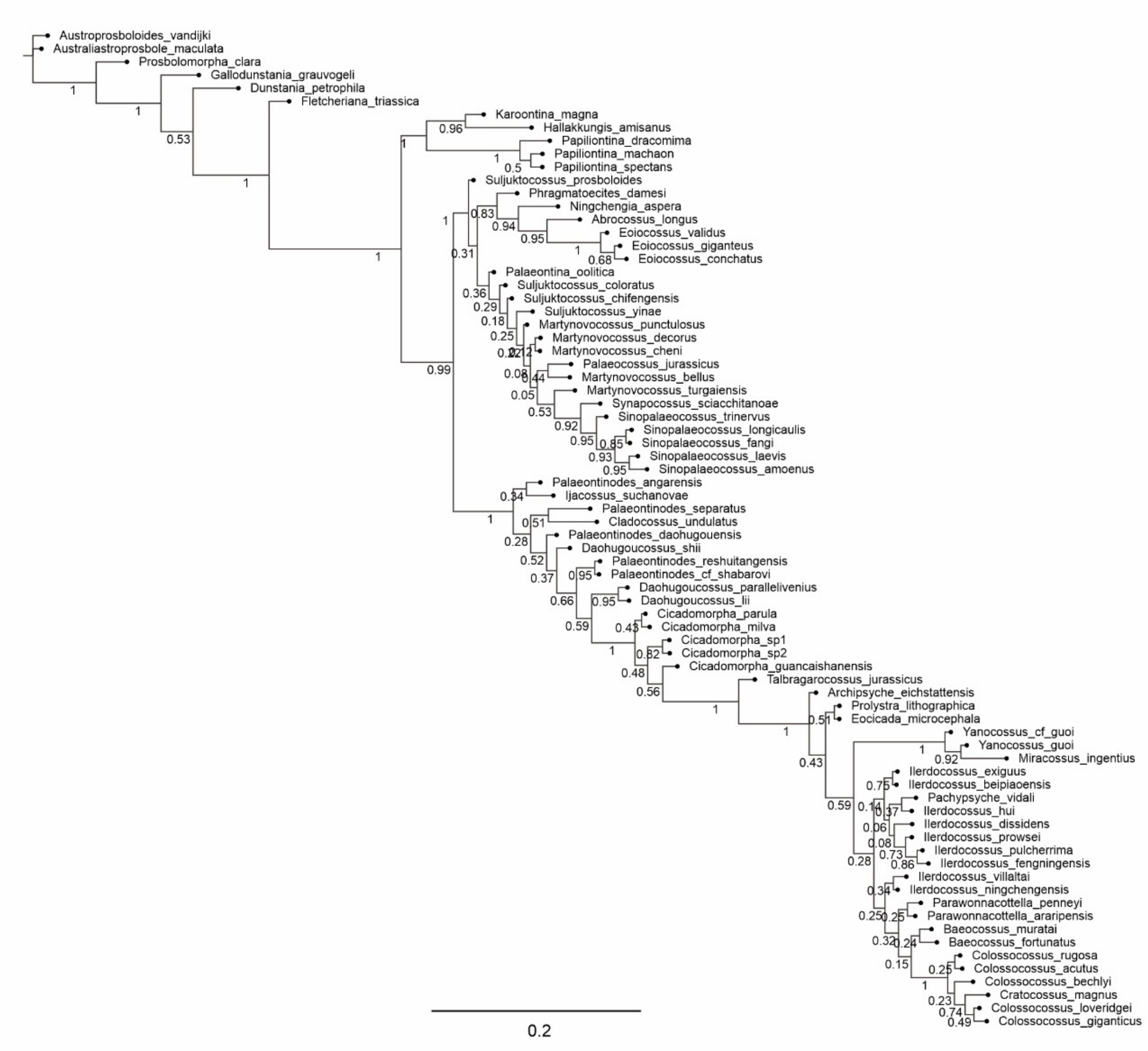

Fig. S11. Result of nonclock Bayesian analysis using Lewis Mkv model with gamma rate variation across characters (Mkv+Γ), based on Phylogenic Matrix 2.

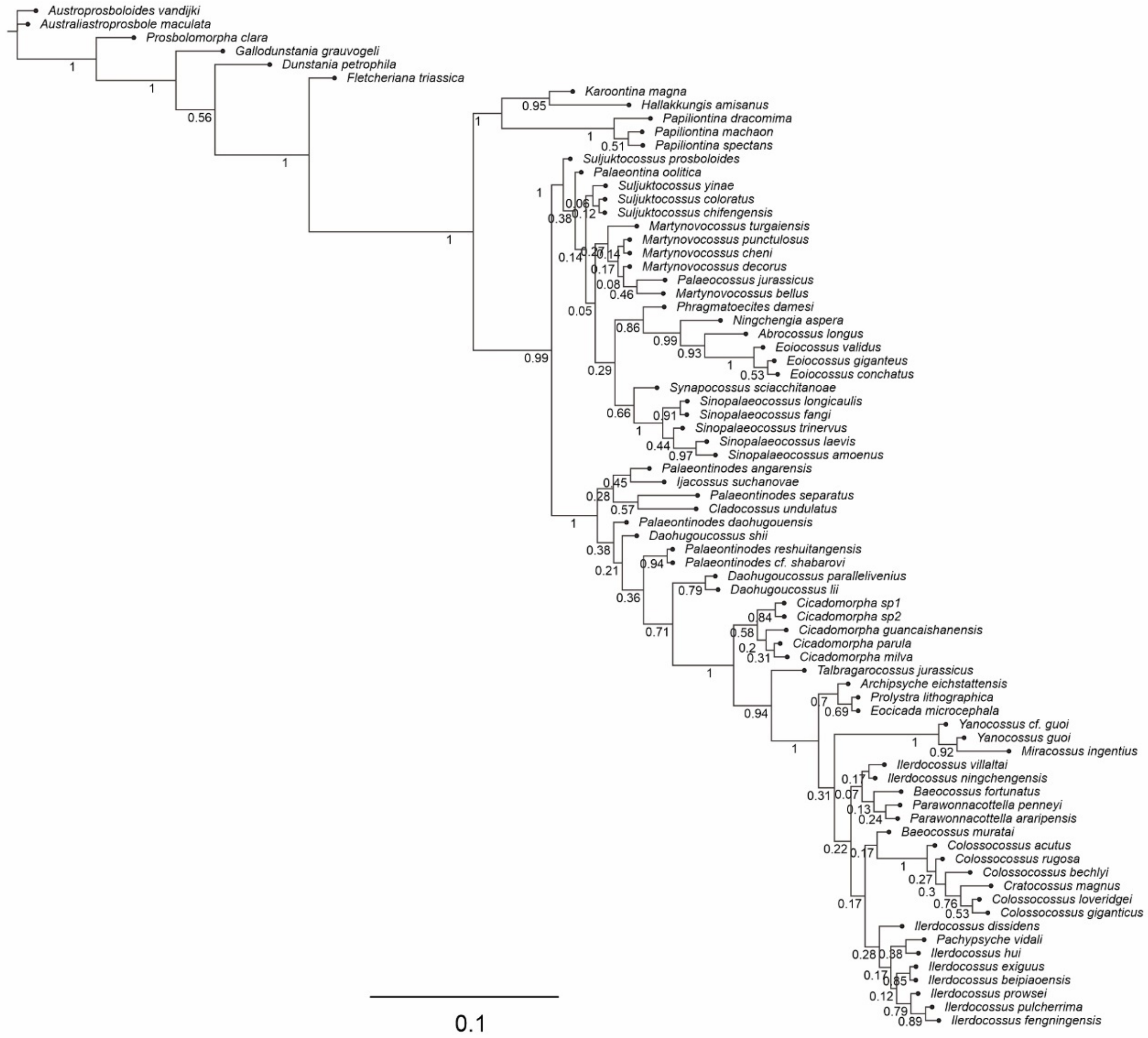

Fig. S12. Result of nonclock Bayesian analysis using Lewis Mkv model with gamma rate variation across characters (Mkv+Γ), based on Phylogenic Matrix 3.

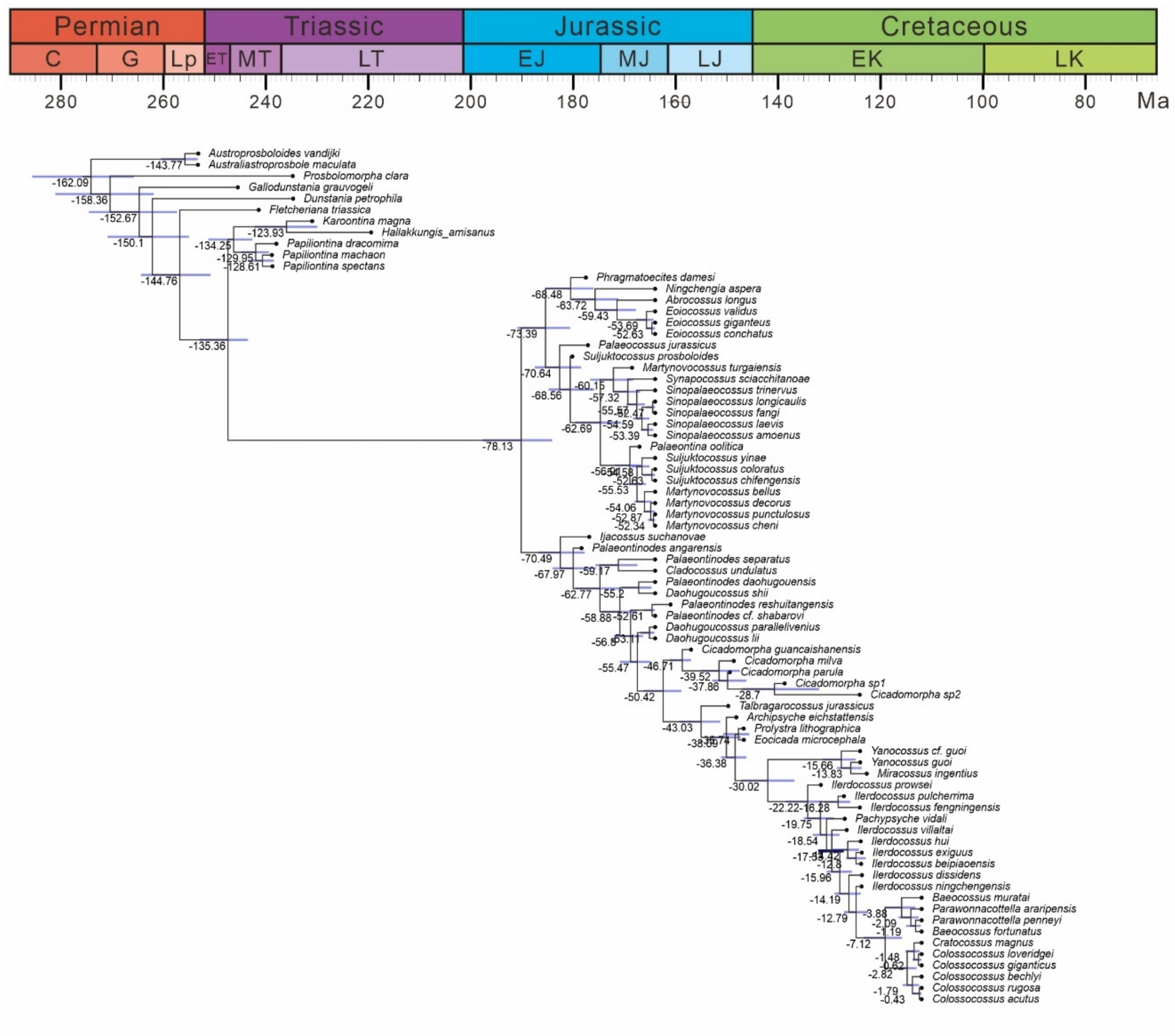

Fig. S13. The consensus tree using Bayesian phylogenetic tip-dating analysis, based on Phylogenic Matrix 3. In the consensus tree (50% majority rule), the node ages (divergence times) are the median estimates (numbers shown in small font) and node bars (blue) represent the 95% highest posterior density intervals.

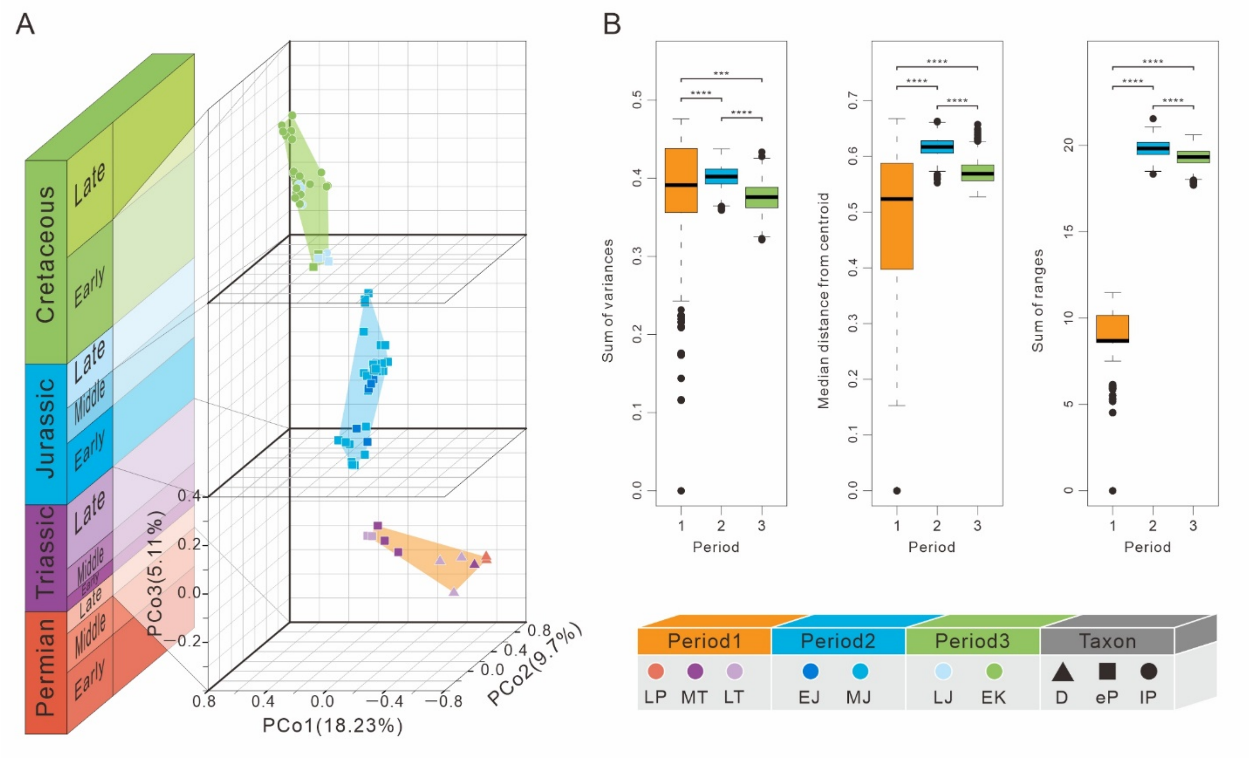

Fig. S14. Morphospace of Dunstaniidae and Palaeontinidae through time, ordinated by principal coordinates analysis (PCoA), maximum observable rescale distance (MORD) matrices, based on the Morphospace Matrix. (**A**) 3D morphospace illustrating the three major axes of variation (corresponding to PC1, PC2, and PC3). (**B**) Comparison of morphological disparity among three periods. The boxes represent the median and the first and third quartiles of morphological disparity (n = 76 species). Morphological disparity was compared using a Welch's t-test for statistical significance (two-sided ****$p < 0.001$, ***$p < 0.01$).

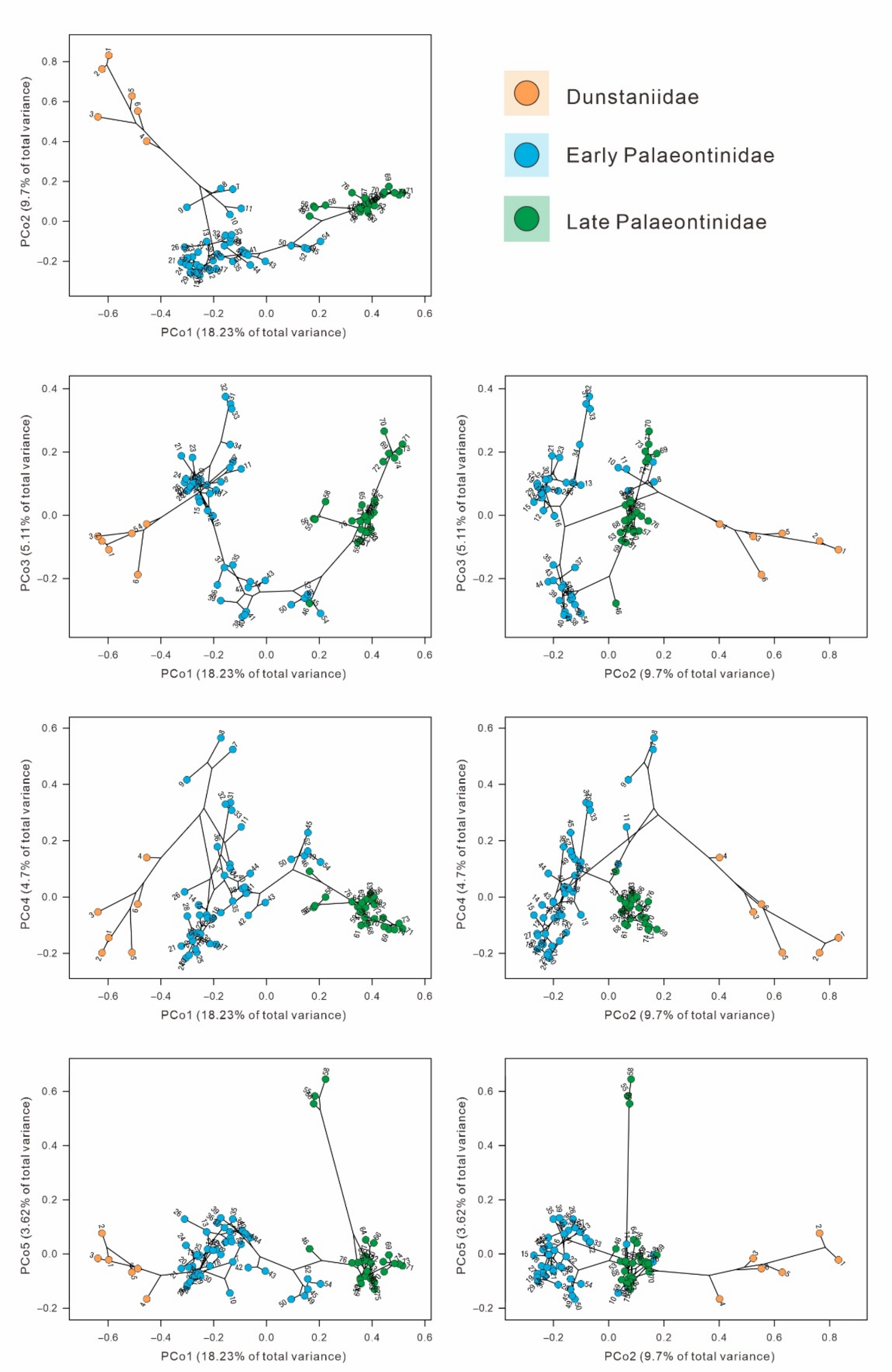

Fig. S15. Morphospace ordinated by principal coordinates analysis (PCoA), maximum observable rescale distance (MORD) matrices, based on Morphospace Matrix.

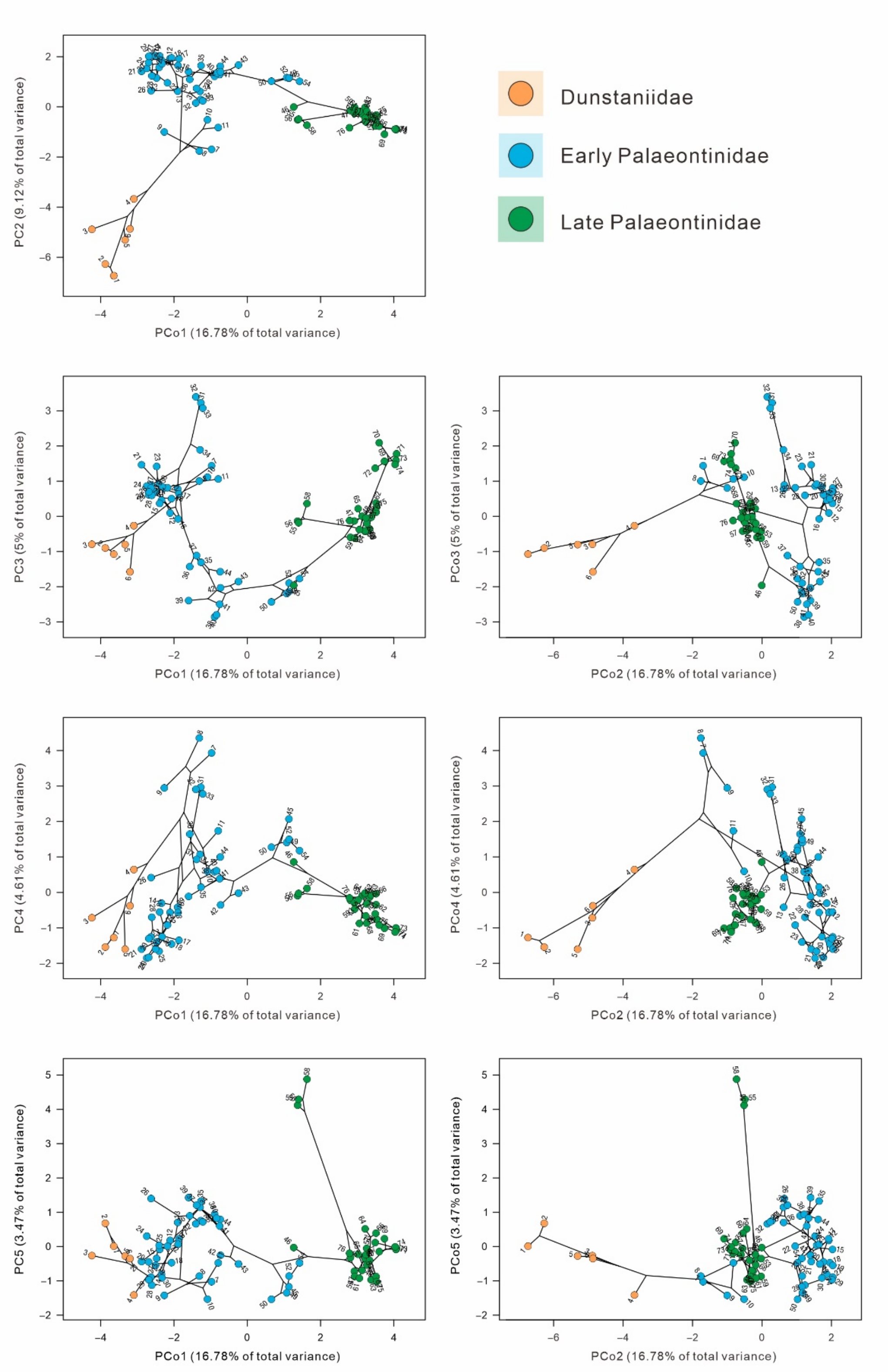

Fig. S16. Morphospace ordinated by principal coordinates analysis (PCoA), generalized Euclidean distance (GED) matrices, based on Morphospace Matrix.

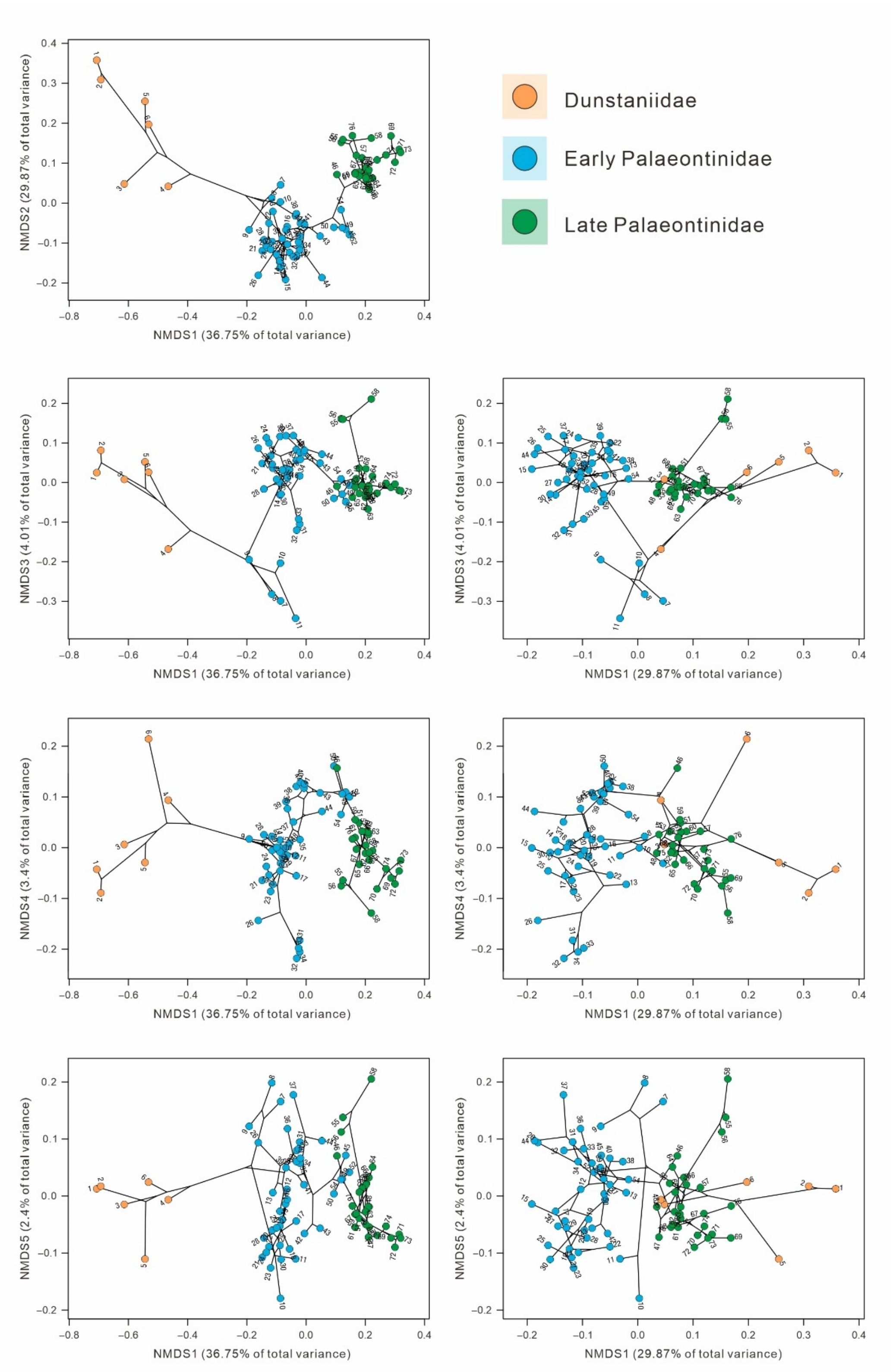

Fig. S17. Morphospace ordinated by non-metric multidimensional scaling (NMDS), maximum observable rescale distance (MORD) matrices, based on Morphospace Matrix.

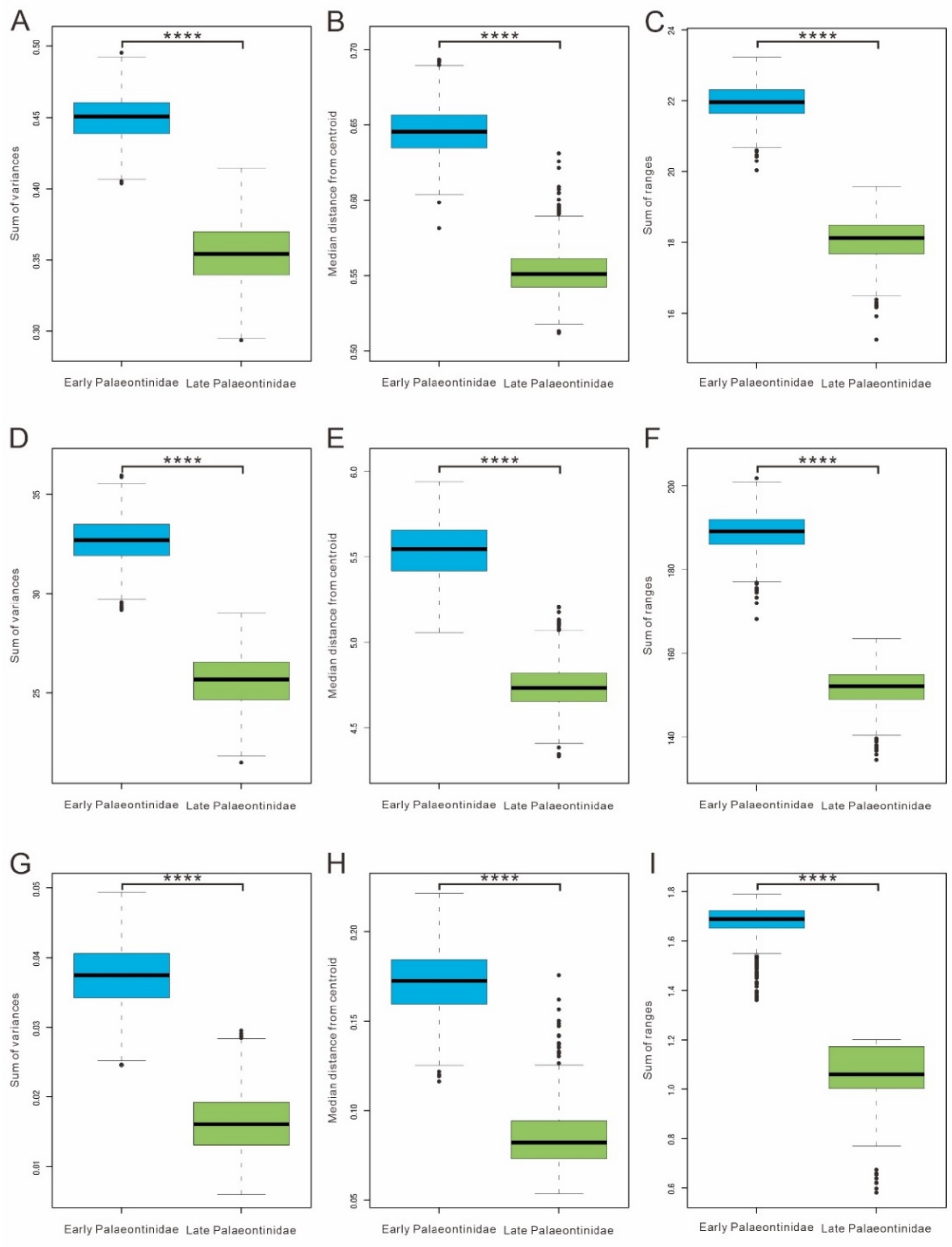

Fig. S18. Comparison of morphological disparity of forewings among Mesozoic Palaeontinidae. The result is derived from the PCoA-MORD-Morphospace Matrix (**A–C**), PCoA-GED-Morphospace Matrix (**D–F**), and NMDS-MORD--Morphospace Matrix (**G–I**). Morphological disparity is quantified using three metrics: sum of variances, median distance from centroid, and sum of ranges. The boxes represent the median, the first and the third quartile of the disparity; n = 76 species. Morphological disparity was compared using Welch's t-test for statistical significance (****two-sided *p*-value threshold <0.001).

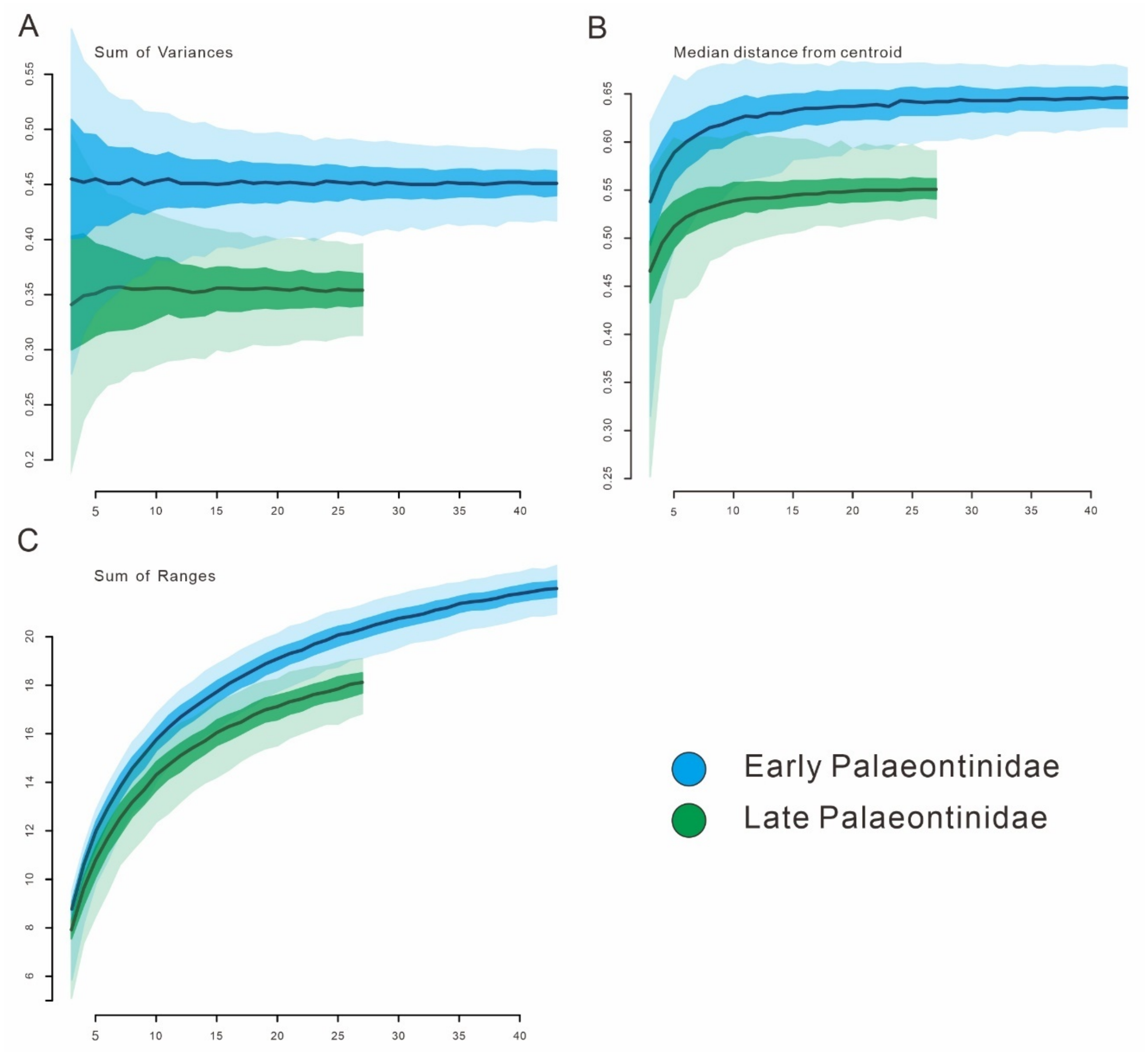

Fig. S19. Rarefaction of disparity curves of Mesozoic Palaeontinidae showing that the results are not strongly affected by sampling bias. Morphological disparity is quantified using three metrices: **A** sum of variances; **B** median distance from centroid; **C** sum of ranges. The dark and light surfaces indicate the 50% and 95% confidence intervals, respectively.

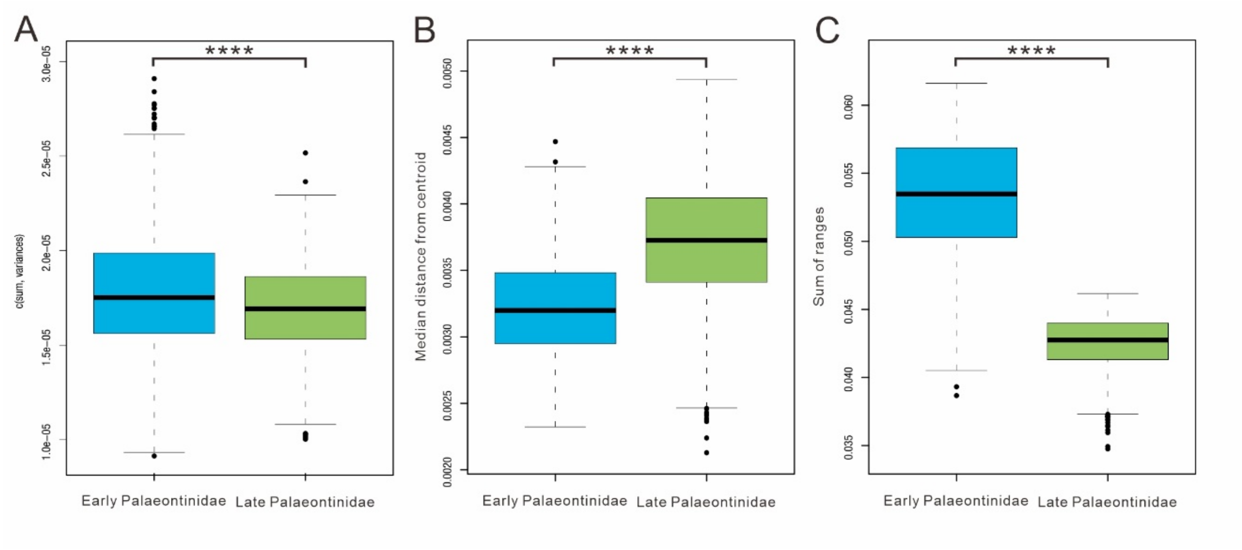

Fig. S20. Comparison of morphological disparity of forewings among Mesozoic Palaeontinidae. The result is derived from the PCA-forewing shape dataset. Morphological disparity is quantified using three metrics: sum of variances, median distance from centroid, and sum of ranges. The boxes represent the median, the first and the third quartile of the disparity; n = 67 species. Morphological disparity was compared using Welch's t-test for statistical significance (****two-sided *p*-value threshold <0.001).

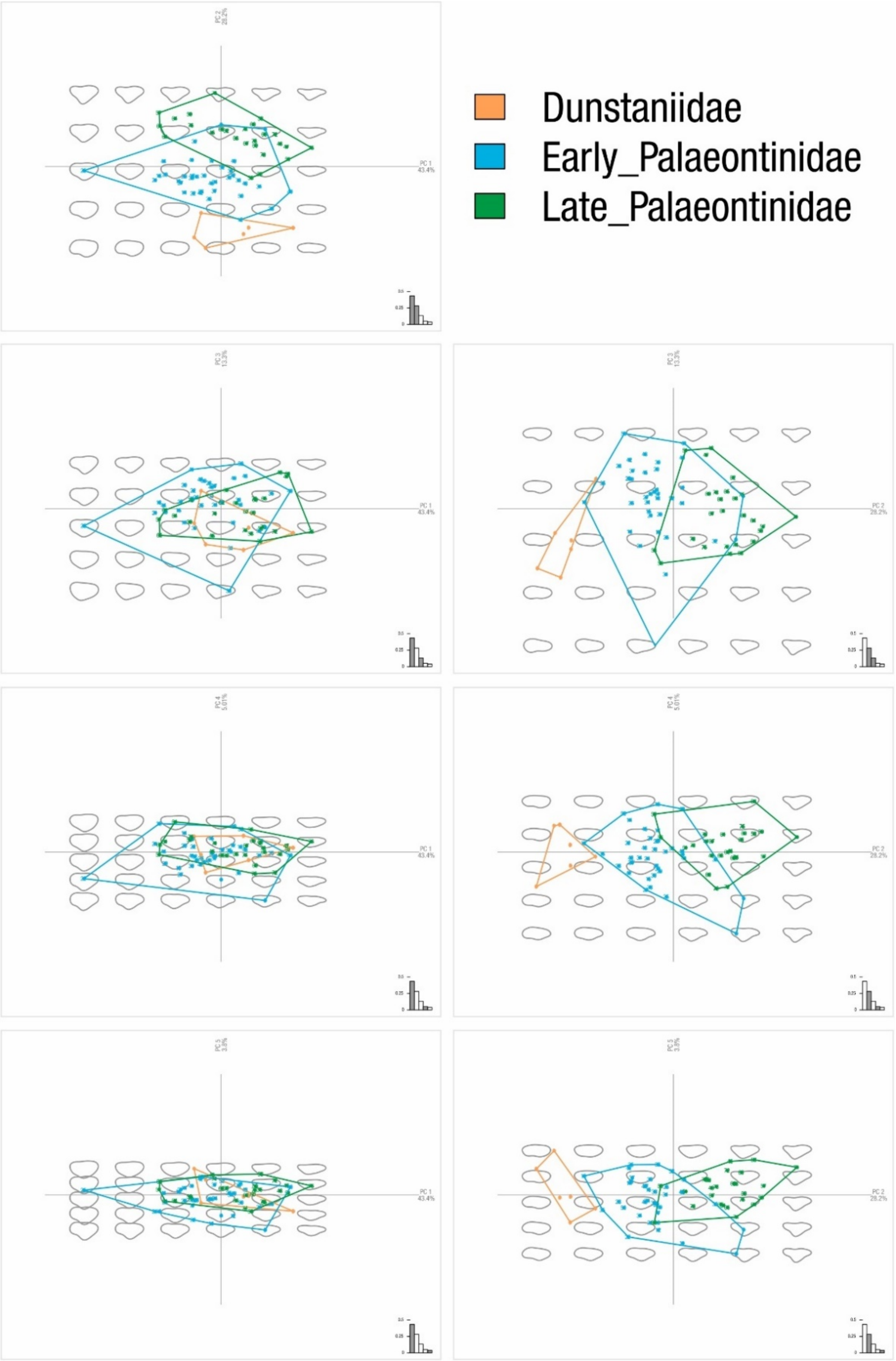

Fig. S21. Morphospace ordinated by principal component analysis (PCA), based on forewing shape dataset.

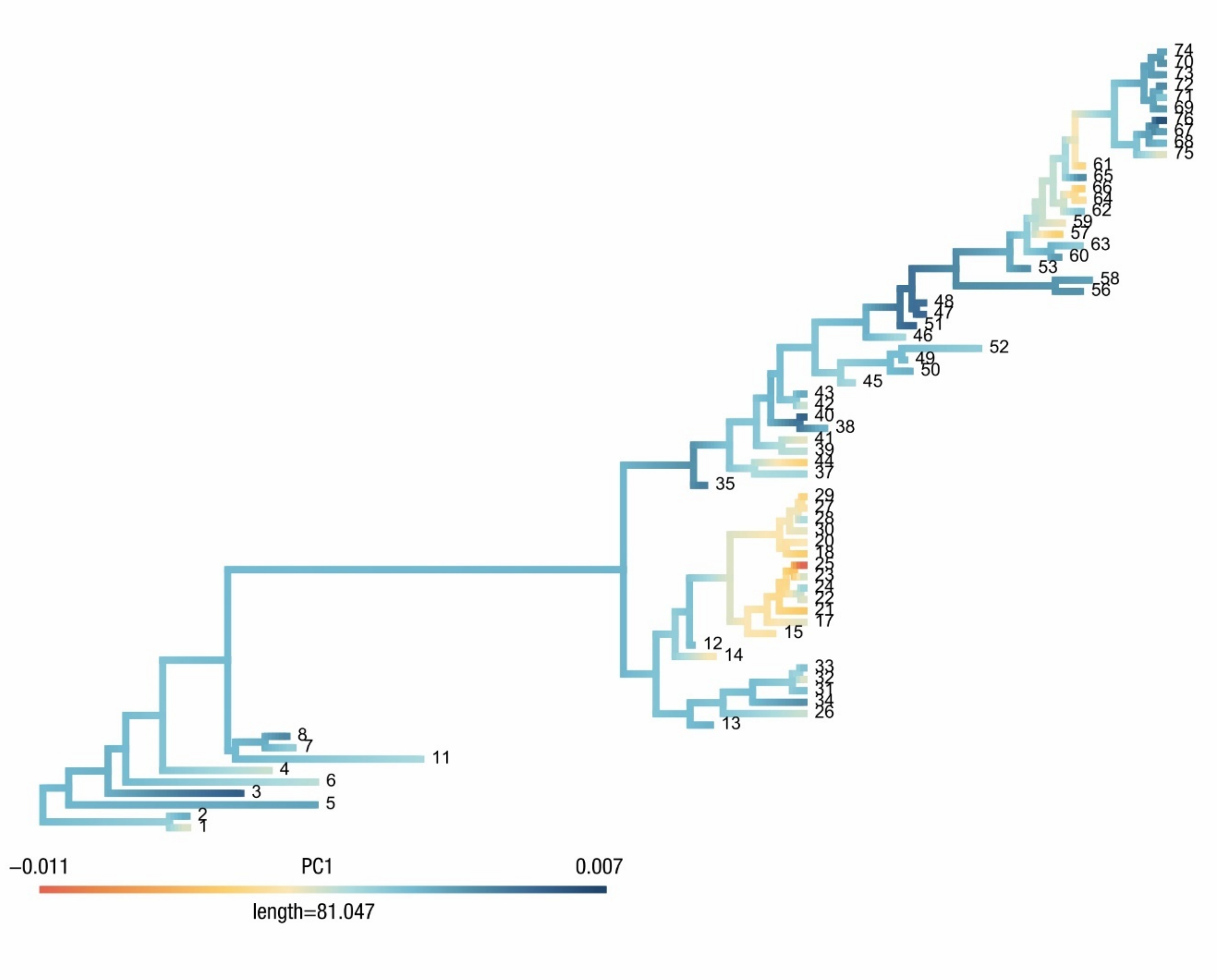

Fig. S22. Evolutionary changes of all forewing characters across time-calibrated phylogeny. The first principal component (PC1) derived from PCA-forewing shape dataset, PC1 (= 44.5% variances).

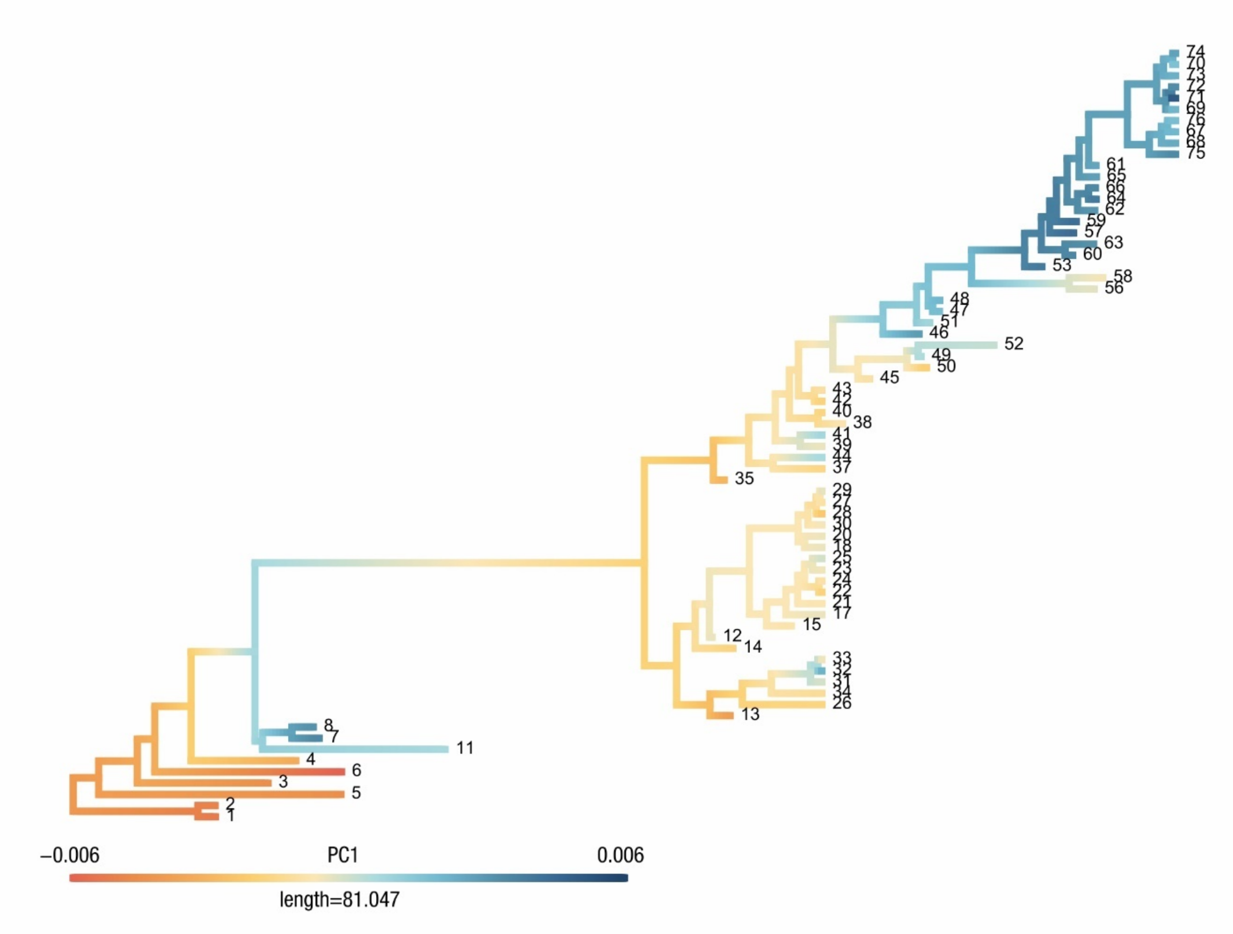

Fig. S23. Evolutionary changes of all forewing characters across time-calibrated phylogeny. The first principal component (PC2) derived from PCA-forewing shape dataset, PC2 (= 28.4% variances).

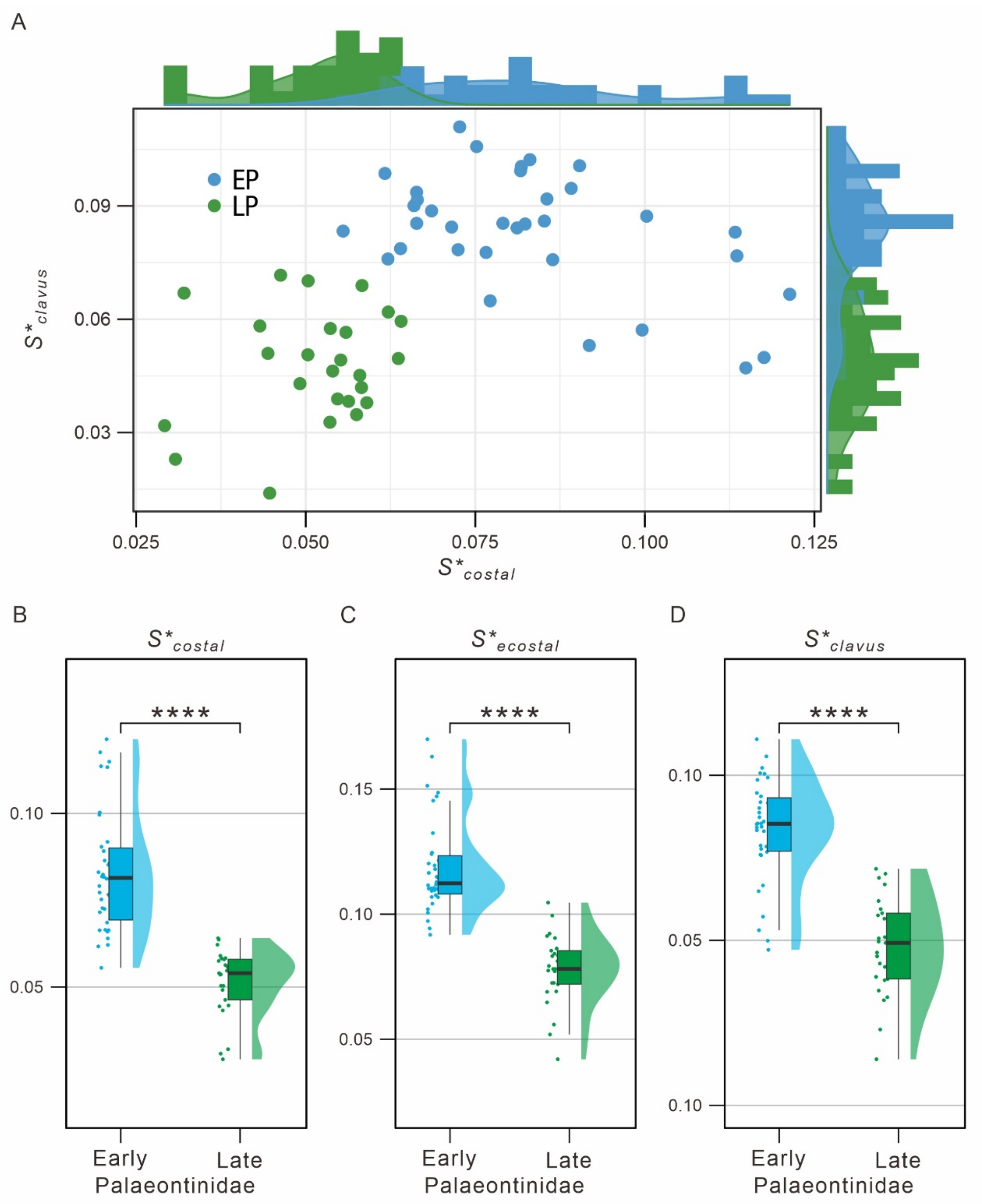

Fig. S24. Morphometric analyses of forewings (costal area, extended costal area, and clavus) between early and late Palaeontinidae. A Costal area versus clavus of early and late Palaeontinidae, with phistogram. Comparison of S^*_{costal} (**B**), S^*_{ecostal} (**C**), and S^*_{clavus} (**D**) between early and late Palaeontinidae. The boxes represent the median, the first and the third quartile of the disparity; n = 65 species. Morphological disparity was compared using Welch's t-test for statistical significance (****two-sided *p*-value threshold <0.001).

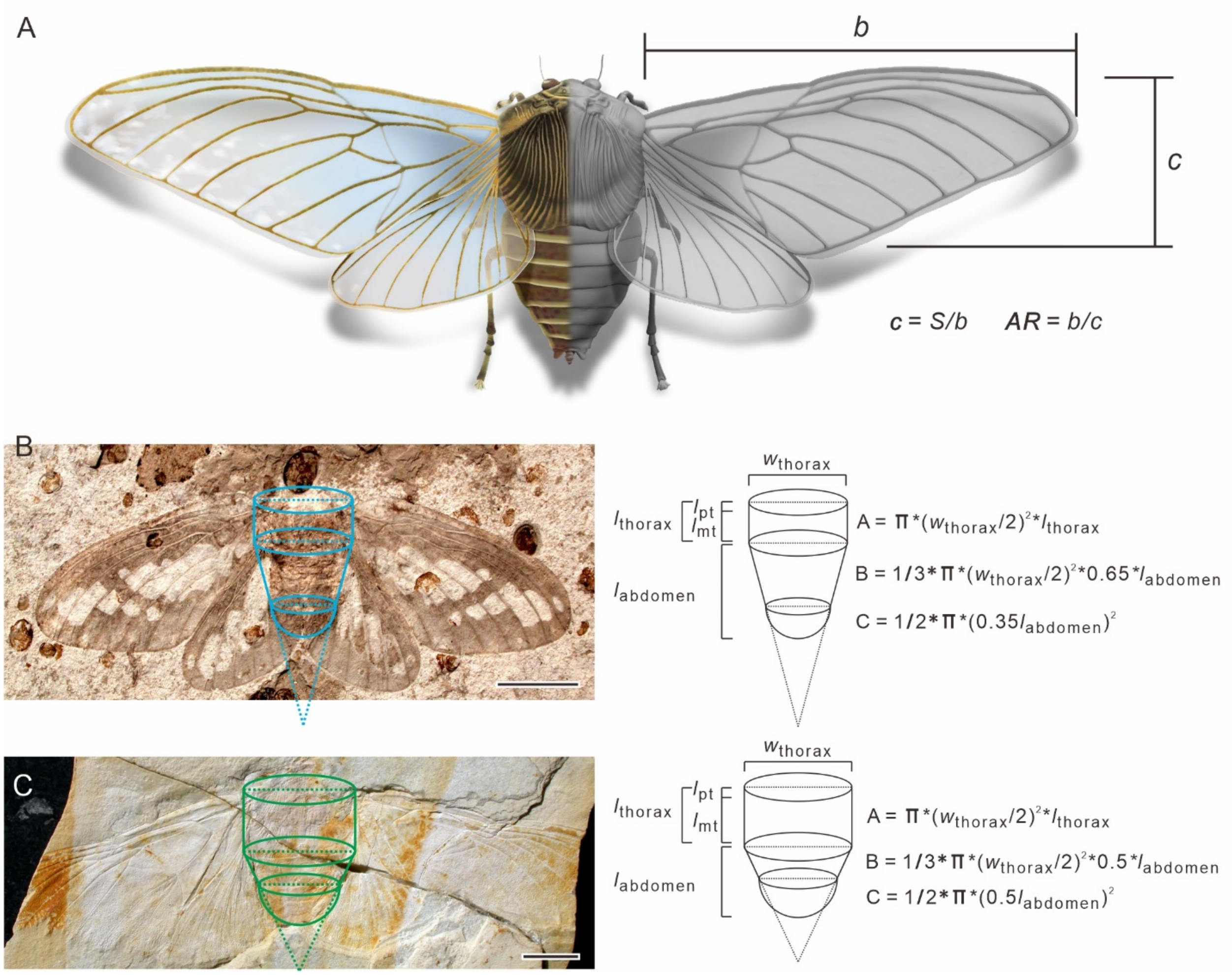

Fig. S25. Simplified geometric body models for estimating the wing morphology parameters (**A**), body morphology parameters of early Palaeontinidae (**B**) and late Palaeontinidae (**C**). Scale bars, 10 mm. All to scale.

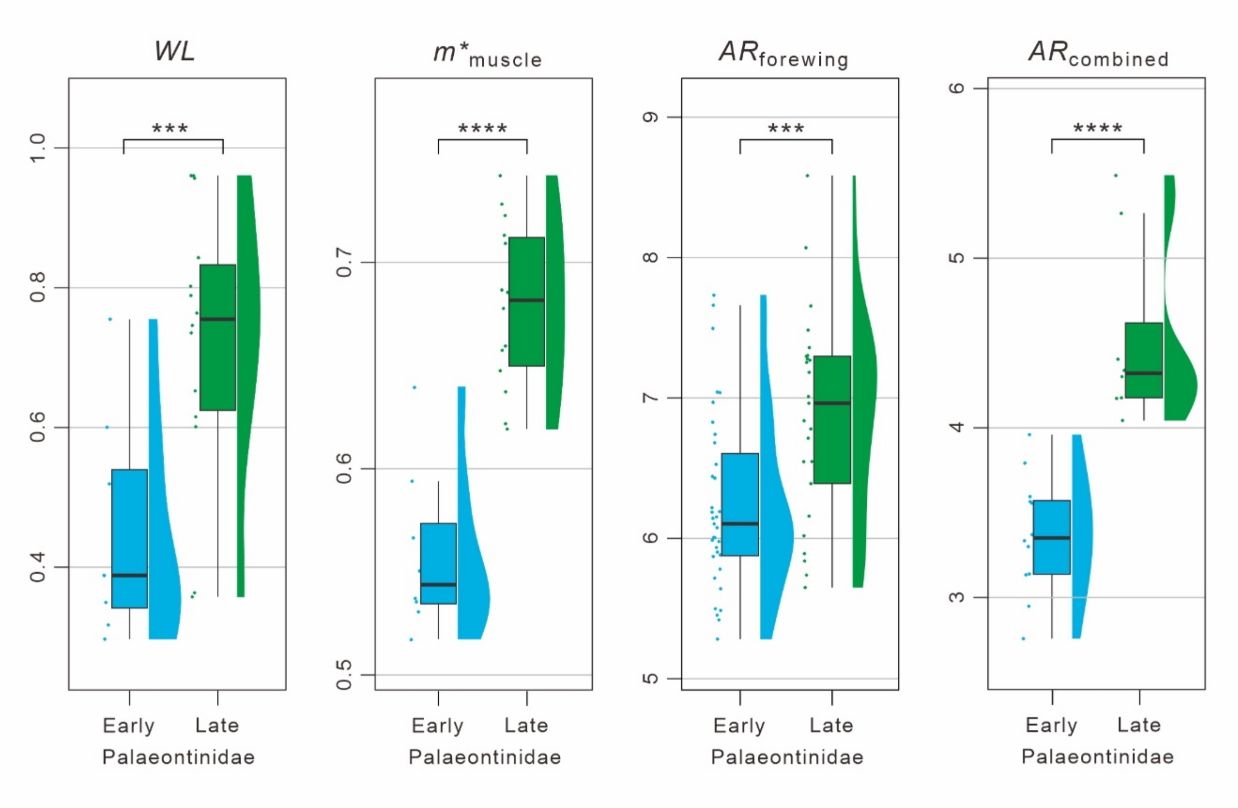

Fig. S26. Comparison of morphological parameters among early and late Palaeontinidae. Two-sided Welch's t-test for statistical significance (****p < 0.001, ***p < 0.01, **p < 0.05, NS = not significant).

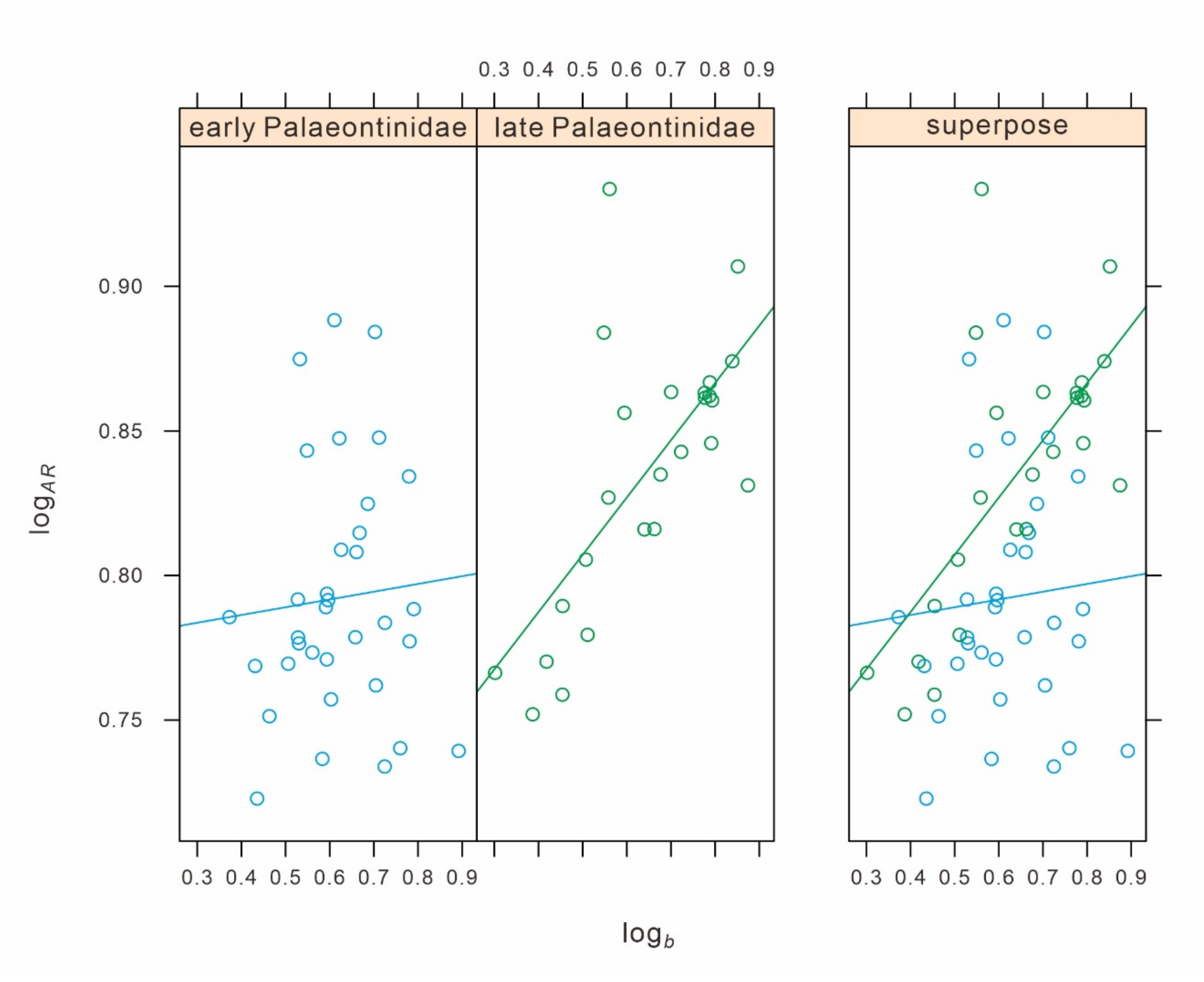

Fig. S27. Allometric relationships between wingspan (*b*) and aspect ratio (*AR*) of forewings of early and late Palaeontinidae species. Blue and green circles represent early and late Palaeontinidae species, respectively. Early and late regression slopes are significantly different (ANCOVA, $p < 0.05$).

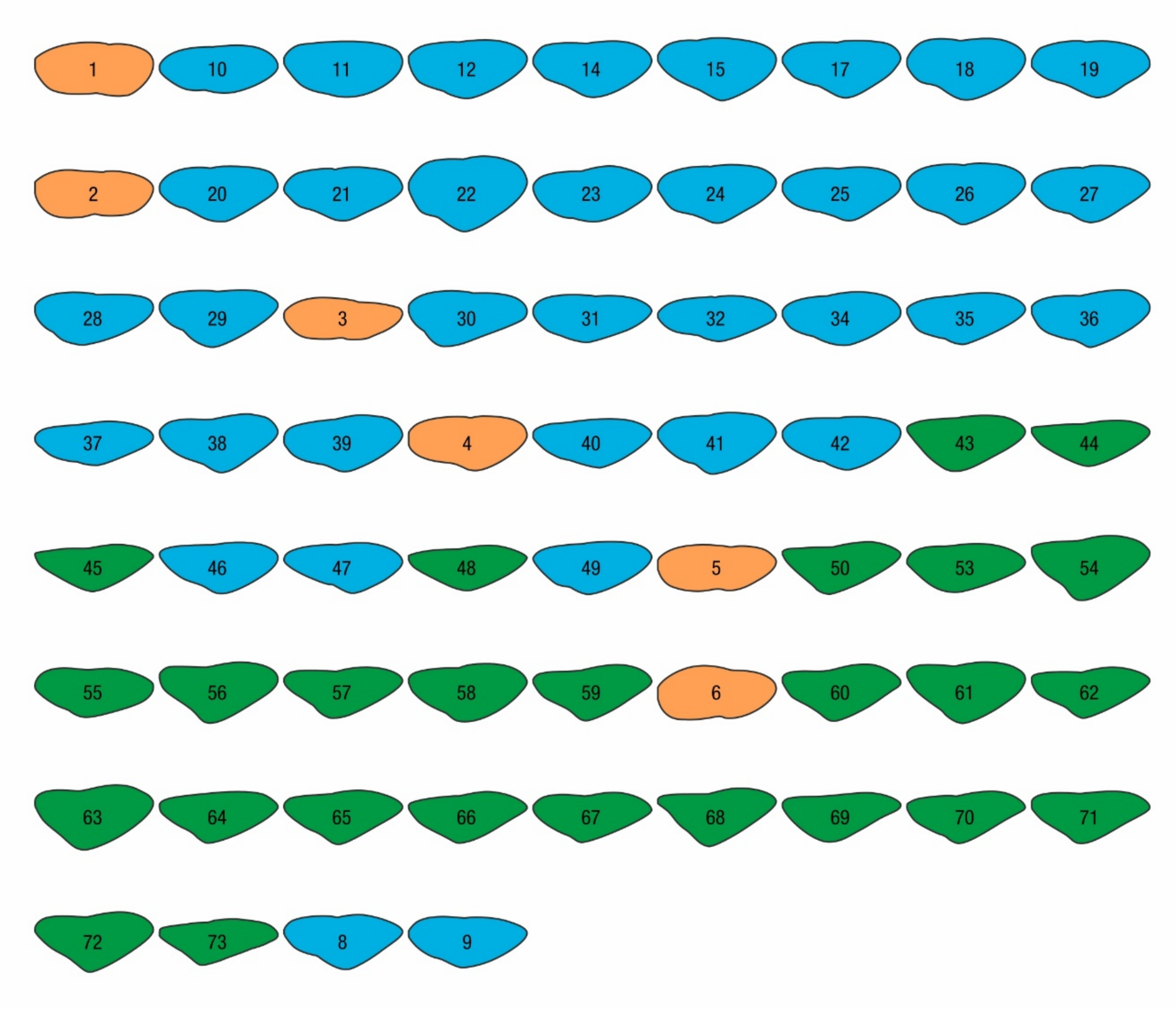

Fig. S28. Forewing shapes used for EFD analyses. Orange, Dunstaniidae; blue, early Palaeontinidae; green, late Palaeontinidae.

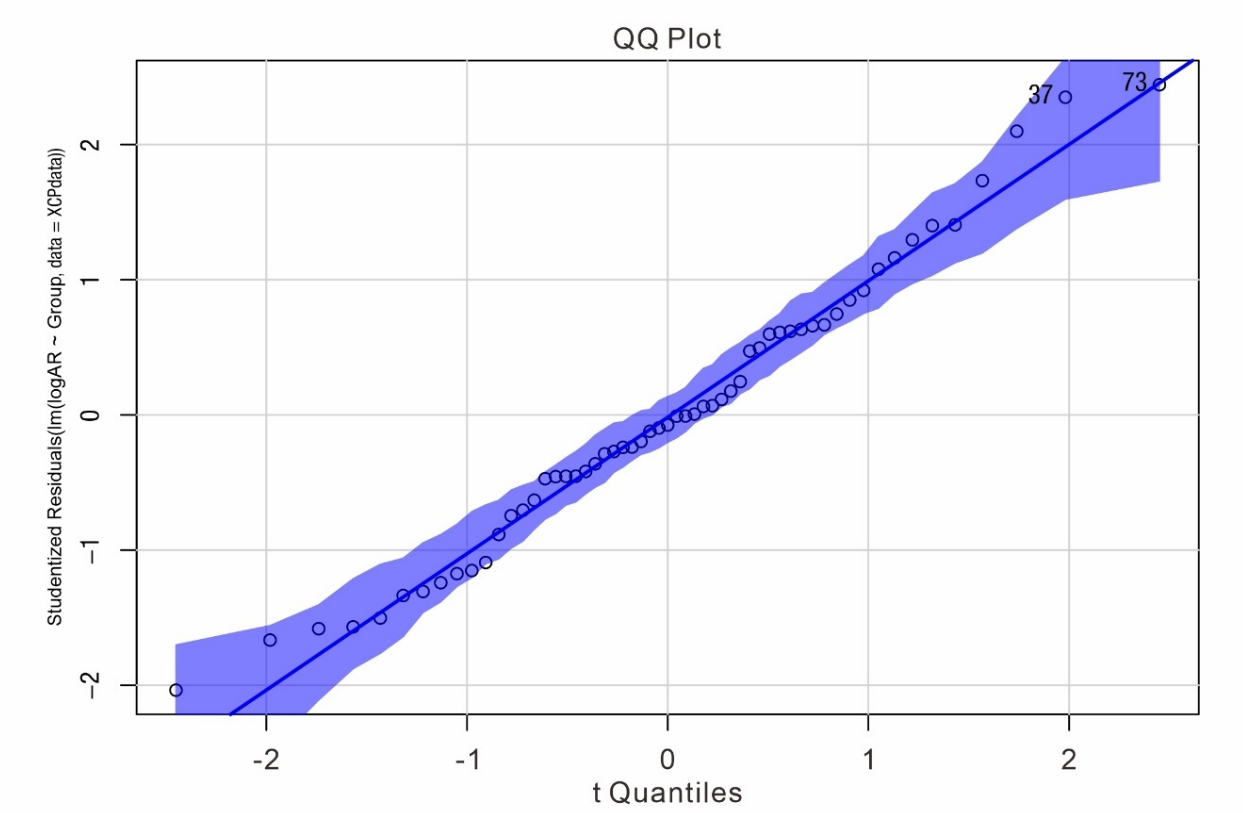

Fig. S29. Normal distribution of Log*L* and Log*AR* of Palaeontinidae.

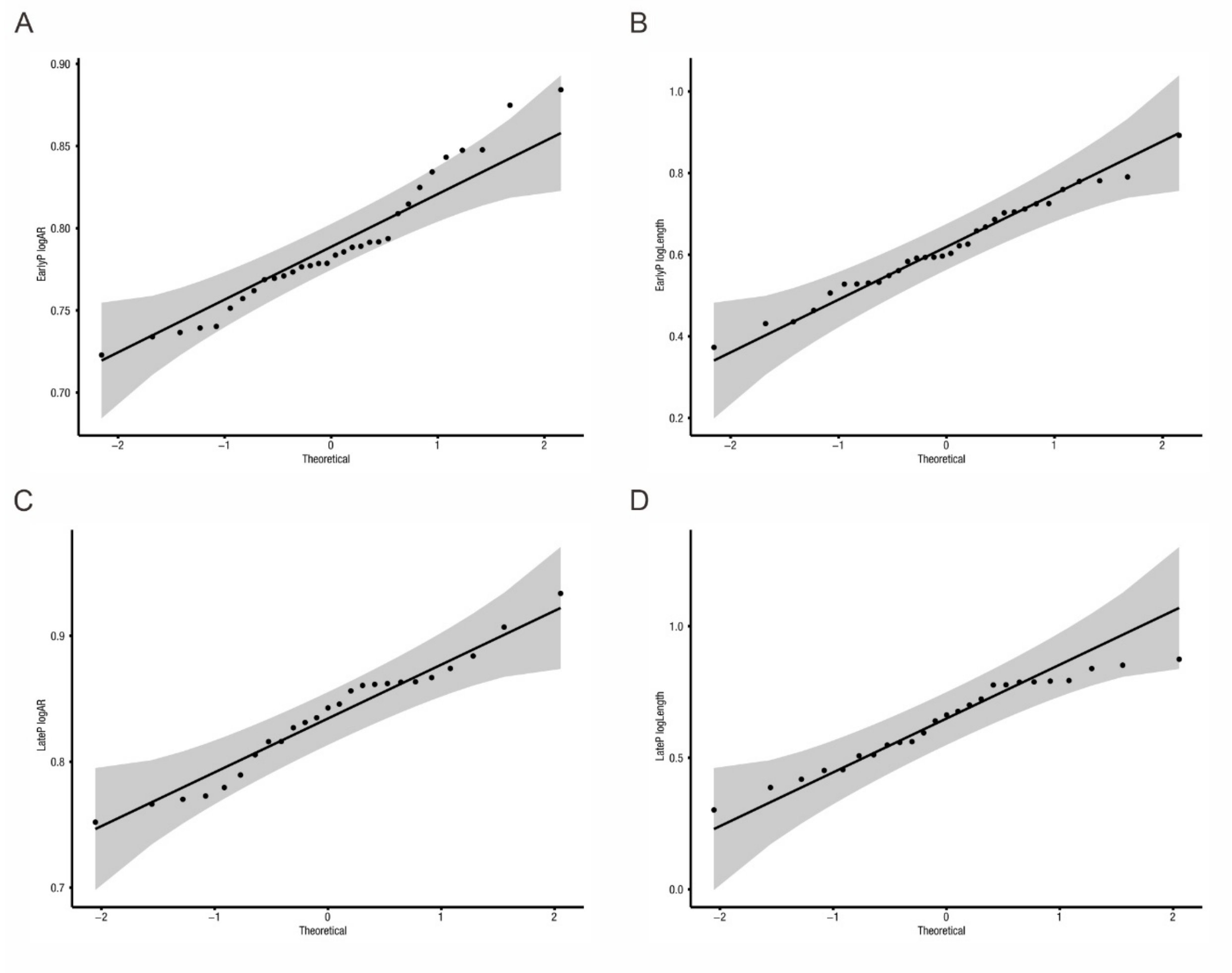

Fig. S30. Normal distribution of Log*L* and Log*AR* of early and late Palaeontinidae.

Table S1. Results of the permanova statistic test of Mesozoic Palaeontinoidea in morphospace of forewing (based on the Morphospace Matrix). (*two-sided *p*-value threshold <0.05)

PCoA based on maximum observable rescale distance (MORD) matrices			
pairwise comparison	*F* Model	R^2	*p* (adjusted)
Dunstaniidae vs early Palaeontinidae	36.37263	0.436265	0.003*
Dunstaniidae vs late Palaeontinidae	28.88893	0.482375	0.003*
early Palaeontinidae vs late Palaeontinidae	49.01318	0.418868	0.003*
**PCoA based on generalized Euclidean distance (GED) matrices**			
pairwise comparison	*F* Model	R^2	*p* (adjusted)
Dunstaniidae vs early Palaeontinidae	34.85662	0.425825	0.003*
Dunstaniidae vs late Palaeontinidae	28.99746	0.483311	0.003*
early Palaeontinidae vs late Palaeontinidae	48.34867	0.415550	0.003*
**NMDS based on maximum observable rescale distance (MORD) matrices**			
pairwise comparison	*F* Model	R^2	*p* (adjusted)
Dunstaniidae vs early Palaeontinidae	36.11424	0.434513	0.003*
Dunstaniidae vs late Palaeontinidae	28.77239	0.481366	0.003*
early Palaeontinidae vs late Palaeontinidae	50.57014	0.426500	0.003*

Table S2. Results of principal components analysis using forewing elements of Mesozoic Palaeontinoidea (principal coordinates analysis PCoA based on Morphospace Matrix).

PCoA based on maximum observable rescale distance (MORD) matrices					
	PC1	PC2	PC3	PC4	PC5
Standard deviation	0.3184	0.2323	0.1686	0.1617	0.1420
Coefficient of variation	-1.03e+16	-5.63e+15	-4.72e+15	-1.19e+16	3.79e+15
Proportion of variance	0.1823	0.0970	0.0511	0.0470	0.0362
Cumulative proportion	0.1823	0.2793	0.3304	0.3775	0.4137

PCoA based on generalized Euclidean distance (GED) matrices					
	PC1	PC2	PC3	PC4	PC5
Standard deviation	2.5600	1.8871	1.3977	1.3416	1.1634
Coefficient of variation	1.13e+17	-1.18e+16	-3.96e+16	1.15e+16	2.68e+15
Proportion of variance	0.1678	0.0912	0.0500	0.0461	0.0347
Cumulative proportion	0.1678	0.2590	0.3091	0.3552	0.3898

Table S3. Results of Welch's t-test of Mesozoic Palaeontinidae (early Palaeontinidae: late Palaeontinidae) in disparity matrices of forewing elements (principal coordinates analysis PCoA based on Morphospace Matrix). (*two-sided p-value threshold <0.05)

PCoA based on maximum observable rescale distance (MORD) matrices			
	t	df	*p*
Sum of variances	112.99	1902	< 2.2e-16*
Median distance from centroids	124.68	1993.2	< 2.2e-16*
Sum of ranges	156.84	1928.9	< 2.2e-16*
**PCoA based on generalized Euclidean distance (GED) matrices**			
	t	df	*p*
Sum of variances	126.44	1965.8	< 2.2e-16*
Median distance from centroids	117.25	1967.3	< 2.2e-16*
Sum of ranges	180.08	1997.4	< 2.2e-16*
**NMDS based on maximum observable rescale distance (MORD) matrices**			
	t	df	*p*
Sum of variances	105.83	1980.2	< 2.2e-16*
Median distance from centroids	111.19	1992	< 2.2e-16*
Sum of ranges	139.78	1503.2	< 2.2e-16*

Table S4. Results of Welch's t-test for Mesozoic Palaeontinidae (early Palaeontinidae: late Palaeontinidae) in disparity matrices of forewing elements (principal coordinates analysis PCoA based on forewing shapes). (*two-sided p-value threshold <0.05)

	t	df	*p*
Sum of variances	5.1932	1844.2	2.295e-7*
Median distance from centroids	-23.06	1849.2	< 2.2e-16*
Sum of ranges	71.702	1387.2	< 2.2e-16*

Table S5. Results of principal components analysis using forewing elements of Mesozoic Palaeontinoidea (principal coordinates analysis PCoA based on forewing shapes).

	PC1	PC2	PC3	PC4	PC5
Standard deviation	0.003219	0.002597	0.001781	0.001094	0.0009532
Proportion of variance	0.433940	0.282480	0.132790	0.050130	0.0380400
Cumulative proportion	0.43394	0.71642	0.84921	0.89934	0.93738

Table S6. Results of permanova statistic test of Mesozoic Palaeontinoidea in morphospace of forewing (principal coordinates analysis PCoA based on forewing shapes). (*two-sided p-value threshold <0.05)

pairwise comparison	*F* Model	R^2	*p* (adjusted)
Dunstaniidae vs early Palaeontinidae	14.68590678	0.263727532	0.003*
Dunstaniidae vs late Palaeontinidae	22.84193049	0.432269039	0.003*
early Palaeontinidae vs late Palaeontinidae	28.67736202	0.319783738	0.003*

Table S7. Comparison of the morphometric parameters relative costal wing area (S^*_{costal}), extended costal area ($S^*_{ecostal}$) and relative clavus area (S^*_{clavus}) between early and late Palaeontinidae.

		early Palaeontinidae		late Palaeontinidae
S^*_{costal}	Number	34		25
	Interval	0.056–0.121		0.029–0.064
	Median	0.081		0.054
	Average	0.083		0.051
	Welch's t-test	t	df	p
		8.8404	53.319	4.955e-12
$S^*_{ecostal}$	Number	34		25
	Interval	0.092–0.170		0.042–0.105
	Median	0.112		0.078
	Average	0.119		0.078
	Welch's t-test	t	df	p
		9.5109	56.98	2.295e-13
S^*_{clavus}	Number	34		25
	Interval	0.047–0.111		0.014–0.071
	Median	0.085		0.049
	Average	0.083		0.048
	Welch's t-test	t	df	p
		8.8553	53.381	4.647e-12

Table S8. Comparison of the flight performance parameters based on wing and body morphology between early and late Palaeontinidae. These parameters include wing loading (*WL*), relative muscle mass (m^*_{muscle}) and aspect ratio of the forewings and of the forewings and hindwings combined ($AR_{forewing}$ and $AR_{combined}$, respectively).

		early Palaeontinidae		late Palaeontinidae	
WL	Number	8		14	
	Interval	0.30–0.75		0.36–0.96	
	Median	0.39		0.75	
	Average	0.45		0.72	
	Welch's t-test	t	df		p
		-3.5576	17.178		0.002389
m^*_{muscle}	Number	8		14	
	Interval	0.52–0.64		0.62–0.74	
	Median	0.54		0.68	
	Average	0.56		0.72	
	Welch's t-test	t	df		p
		-6.7298	14.612		7.731e-06
$AR_{forewing}$	Number	35		25	
	Interval	5.28–7.73		5.65–8.58	
	Median	6.10		6.96	
	Average	6.24		6.87	
	Welch's t-test	t	df		p
		-3.4587	46.622		0.00117
$AR_{combined}$	Number	12		8	
	Interval	2.76–3.96		4.04–5.50	
	Median	3.35		4.32	
	Average	3.37		4.52	
	Welch's t-test	t	df		p
		-5.343	10.821		0.0002499

Table S9. Information about measurement parameters of wings and bodies.

Symbol	Parameter	unit
AR	wing aspect ratio	-
AR_{forewing}	aspect ratio of the forewing	-
AR_{combined}	aspect ratio of the forewing and hindwing combined	-
b	wingspan	cm
c	mean chord	cm
C_L	lift coefficient	-
D	Aerodynamic drag force	N
g	Gravitational acceleration	m/s^2
L	Aerodynamic lift force	N
l_{thorax}	thoracic length	cm
l_{pt}	prothoracic length	cm
l_{mt}	mesothoracic length	cm
l_{abdomen}	abdominal length	cm
m_{body}	body mass	g
m_{muscle}	muscle mass	g
$m*_{\text{muscle}}$	muscle mass relative to body mass	-
P_{avail}	Power available from flight power muscles	W
P_{req}	Power required for flight	W
r_{air}	air density	kg/m^3
$\mathrm{r}_{\text{water}}$	water density	kg/m^3
S	wing area	cm^2
S_{clavus}	clavus area	cm^2
S_{costal}	costal area	cm^2
S_{ecostal}	extend costal area	cm^2
$S*_{\text{clavus}}$	Ratio between clavus area to wing area	-
$S*_{\text{costal}}$	Ratio between costal area and wing area	-
$S*_{\text{ecostal}}$	Ratio between extend costal area and wing area	-
U	Flight speed	m/s
V_{body}	body volume	cm^3
V_{thorax}	thoracic volume	cm^3
WL	wing loading	g/cm^2
w_{thorax}	thoracic width	cm

Table S10. Seventy-six undisputed species selected for phylogenetic and morphospace analyses.

Num	Species	Num	Species
1	*Austroprosboloides vandijki*	39	*Palaeontinodes daohugouensis*
2	*Australiastroprosbole maculata*	40	*Palaeontinodes cf. shabarovi*
3	*Gallodunstania grauvogeli*	41	*Daohugoucossus shii*
4	*Fletcheriana triassica*	42	*Daohugoucossus parallelivenius*
5	*Prosbolomorpha clara*	43	*Daohugoucossus lii*
6	*Dunstania petrophila*	44	*Cladocossus undulatus*
7	*Papiliontina dracomima*	45	*Cicadomorpha guancaishanensis*
8	*Papiliontina machaon*	46	*Talbragarocossus jurassicus*
9	*Papiliontina spectans*	47	*Prolystra lithographica*
10	*Karoontina magna*	48	*Eocicada microcephala*
11	*Hallakkungis amisanus*	49	*Cicadomorpha parula*
12	*Suljuktocossus prosboloides*	50	*Cicadomorpha milva*
13	*Phragmatoecites damesi*	51	*Archipsyche eichstattensis*
14	*Palaeocossus jurassicus*	52	*Cicadomorpha* sp. 1
15	*Martynovocossus turgaiensis*	53	*Ilerdocossus prowsei*
16	*Palaeontina oolitica*	54	*Cicadomorpha* sp. 2
17	*Synapocossus sciacchitanoae*	55	*Yanocossus guoi*
18	*Suljuktocossus yinae*	56	*Yanocossus cf guoi*
19	*Suljuktocossus coloratus*	57	*Pachypsyche vidali*
20	*Suljuktocossus chifengensis*	58	*Miracossus ingentius*
21	*Sinopalaeocossus trinervus*	59	*Ilerdocossus villaltai*
22	*Sinopalaeocossus longicaulis*	60	*Ilerdocossus pulcherrima*
23	*Sinopalaeocossus laevis*	61	*Ilerdocossus ningchengensis*
24	*Sinopalaeocossus fangi*	62	*Ilerdocossus hui*
25	*Sinopalaeocossus amoenus*	63	*Ilerdocossus fengningensis*
26	*Ningchengia aspera*	64	*Ilerdocossus exiguus*
27	*Martynovocossus punctulosus*	65	*Ilerdocossus dissidens*
28	*Martynovocossus decorus*	66	*Ilerdocossus beipiaoensis*
29	*Martynovocossus cheni*	67	*Parawonnacottella penneyi*
30	*Martynovocossus bellus*	68	*Parawonnacottella araripensis*
31	*Eoiocossus validus*	69	*Cratocossus magnus*
32	*Eoiocossus giganteus*	70	*Colossocossus rugosa*
33	*Eoiocossus conchatus*	71	*Colossocossus loveridgei*
34	*Abrocossus longus*	72	*Colossocossus giganticus*
35	*Palaeontinodes angarensis*	73	*Colossocossus bechlyi*
36	*Ijacossus suchanovae*	74	*Colossocossus acutus*
37	*Palaeontinodes separatus*	75	*Baeocossus muratai*
38	*Palaeontinodes reshuitangensis*	76	*Baeocossus fortunatus*

Movie S1 (separate file). 3D Chronophylomorphospace showing the expansion of morphologies on the two major axes of variation PC1 and PC2 through time. Morphospace ordinated by principal coordinates analysis (PCoA), maximum observable rescale distance (MORD) matrices, and based on Morphospace Matrix.

Dataset S1 (separate file). Diversity database of Mesozoic Dunstaniidae and Palaeontinidae.

Dataset S2 (separate file). Phylogenic Matrix 1 on 119 characters of 92 species of Dunstaniidae and Palaeontinidae.

Dataset S3 (separate file). Phylogenic Matrix 2 on 119 characters of 76 species of Dunstaniidae and Palaeontinidae.

Dataset S4 (separate file). Phylogenic Matrix 3 on 77 characters of 76 species of Dunstaniidae and Palaeontinidae.

Dataset S5 (separate file). Morphospace Matrix 1 on 86 characters of 76 species of Dunstaniidae and Palaeontinidae.

Dataset S6 (separate file). Basic morphometric results of forewing of 65 species of Dunstaniidae and Palaeontinidae.

Dataset S7 (separate file). Geometric results of forewing outlines of 65 species of Dunstaniidae and Palaeontinidae.

Dataset S8 (separate file). Geometric results of combined wing outlines of 21 species of Dunstaniidae and Palaeontinidae.

Dataset S9 (separate file). Geometric results of bodies of 22 species of Dunstaniidae and Palaeontinidae.

REFERENCES AND NOTES

1. R. J. Wootton, J. Kukalová-Peck, D. J. S. Newman, J. Muzón, Smart engineering in the mid-Carboniferous: How well could Palaeozoic dragonflies fly? *Science* **282**, 749–751 (1998).

2. D. E. Alexander, *On the Wing: Insects*, *Pterosaurs*, *Birds*, *Bats and the Evolution of Animal Flight* (Oxford Univ. Press, 2015).

3. A. Biewener, S. N. Patek, *Animal Locomotion* (Oxford Univ. Press, 2018).

4. C. Le Roy, D. Amadori, S. Charberet, J. Windt, F. T. Muijres, V. Llaurens, V. Debat, Adaptive evolution of flight in *Morpho* butterflies. *Science* **374**, 1158–1162 (2021).

5. M. H. Menz, M. Scacco, H. M. Bürki-Spycher, H. J. Williams, D. R. Reynolds, J. W. Chapman, M. Wikelski, Individual tracking reveals long-distance flight-path control in a nocturnally migrating moth. *Science* **377**, 764–768 (2022).

6. R. J. Wootton, Functional morphology of insect wings. *Annu. Rev. Entomol.* **37**, 113–140 (1992).

7. R. Dudley, *The Biomechanics of Insect Flight: Form*, *Function*, *Evolution* (Princeton Univ. Press, 2002).

8. R. J. Wootton, J. Kukalová-Peck, Flight adaptations in Palaeozoic Palaeoptera (Insecta). *Biol. Rev.* **75**, 129–167 (2000).

9. R. Wootton, The geometry and mechanics of insect wing deformations in flight: A modelling approach. *Insects* **11**, 446 (2020).

10. R. G. Beutel, M. I. Yavorskaya, Y. Mashimo, M. Fukui, K. Meusemann, The phylogeny of Hexapoda (Arthropoda) and the evolution of megadiversity. *Proc. Arthropod. Embryol. Soc. Jpn.* **51**, 1–15 (2017).

11. J. J. Rubin, C. A. Hamilton, C. J. McClure, B. A. Chadwell, A. Y. Kawahara, J. R. Barber, The evolution of anti-bat sensory illusions in moths. *Sci. Adv.* **4**, eaar7428 (2018).

12. C. Le Roy, V. Debat, V. Llaurens, Adaptive evolution of butterfly wing shape: From morphology to behaviour. *Biol. Rev. Camb. Philos. Soc.* **94**, 1261–1281 (2019).

13. S. Mena, K. M. Kozak, R. E. Cardenas, M. F. Checa, Forest stratification shapes allometry and flight morphology of tropical butterflies. *Proc. Biol. Sci.* **287**, 20201071 (2020).

14. S. Sponberg, J. P. Dyhr, R. W. Hall, T. L. Daniel, Luminance-dependent visual processing enables moth flight in low light. *Science* **348**, 1245–1248 (2015).

15. R. P. Ray, T. Nakata, P. Henningsson, R. J. Bomphrey, Enhanced flight performance by genetic manipulation of wing shape in *Drosophila*. *Nat. Commun.* **7**, 10851 (2016).

16. B. R. Aiello, M. Tan, U. Bin Sikandar, A. J. Alvey, B. Bhinderwala, K. C. Kimball, J. R. Barber, C. A. Hamilton, A. Y. Kawahara, S. Sponberg, Adaptive shifts underlie the divergence in wing morphology in bombycoid moths. *Proc. R. Soc. B* **288**, 20210677 (2021).

17. Z. Wei, Z. Wei, S. Wang, S. Farris, N. Chennuri, N. Wang, S. Shinsato, K. Demir, M. Horii, G. X. Gu, Towards silent and efficient flight by combining bioinspired owl feather serrations with cicada wing geometry. *Nat. Commun.* **15**, 4337 (2024).

18. B. Jantzen, T. Eisner, Hindwings are unnecessary for flight but essential for execution of normal evasive flight in Lepidoptera. *Proc. Natl. Acad. Sci. U.S.A.* **105**, 16636–16640 (2008).

19. W. E. Conner, A. J. Corcoran, Sound strategies: The 65-million-year-old battle between bats and insects. *Annu. Rev. Entomol.* **57**, 21–39 (2012).

20. E. Page, L. M. Queste, N. Rosser, P. A. Salazar, N. J. Nadeau, J. Mallet, R. B. Srygley, W. O. McMillan, K. K. Dasmahapatra, Pervasive mimicry in flight behavior among aposematic butterflies. *Proc. Natl. Acad. Sci. U.S.A.* **121**, e2300886121 (2024).

21. M. E. Clapham, J. A. Karr, Environmental and biotic controls on the evolutionary history of insect body size. *Proc. Natl. Acad. Sci. U.S.A.* **109**, 10927–10930 (2012).

22. J. H. Marden, P. Chai, Aerial predation and butterfly design: How palatability, mimicry, and the need for evasive flight constrain mass allocation. *Am. Nat.* **138**, 15–36 (1991).

23. E. I. Svensson, M. Friberg, Selective predation on wing morphology in sympatric damselflies. *Am. Nat.* **170**, 101–112 (2007).

24. A. Chotard, J. Ledamoisel, T. Decamps, A. Herrel, A. S. Chaine, V. Llaurens, V. Debat, Evidence of attack deflection suggests adaptive evolution of wing tails in butterflies. *Proc. R. Soc. B* **289**, 20220562 (2022).

25. Y. Yu, C. Zhang, X. Xu, Deep time diversity and the early radiations of birds. *Proc. Natl. Acad. Sci. U.S.A.* **118**, e2019865118 (2021).

26. M. Wang, Z. Zhou, Low morphological disparity and decelerated rate of limb size evolution close to the origin of birds. *Nat. Ecol. Evol.* **7**, 1257–1266 (2023).

27. Y. Kiat, J. K. O'Connor, Functional constraints on the number and shape of flight feathers. *Proc. Natl. Acad. Sci. U.S.A.* **121**, e2306639121 (2024).

28. X. Xu, H. You, K. Du, F. Han, An *Archaeopteryx*-like theropod from China and the origin of Avialae. *Nature* **475**, 465–470 (2011).

29. D. F. A. E. Voeten, J. Cubo, E. De Margerie, M. Röper, V. Beyrand, S. Bureš, P. Tafforeau, S. Sanchez, Wing bone geometry reveals active flight in *Archaeopteryx*. *Nat. Commun.* **9**, 923 (2018).

30. J. Chen, R. Beattie, B. Wang, H. Jiang, Y. Zheng, H. Zhang, The first palaeontinid from the Late Jurassic of Australia (Hemiptera, Cicadomorpha, Palaeontinidae). *Alcheringa* **43**, 449–454 (2019).

31. R. J. Wootton, Reconstructing insect flight performance from fossil evidence. *Acta Zool. Cracov.* **46**, 89–99 (2003).

32. F. Menon, S. W. Heads, New species of Palaeontinidae (Insecta, Cicadomorpha) from the Lower Cretaceous Crato Formation of Brazil. *Stuttgarter Beitr. Naturk. Ser. B.* **357**, 1–11 (2005).

33. B. Wang, H. C. Zhang, J. Szwedo, Jurassic Palaeontinidae from China and the higher systematics of Palaeontinoidea (Insecta: Hemiptera: Cicadomorpha). *Palaeontology* **52**, 53–64 (2009).

34. Y. Wang, C. Shih, J. Szwedo, D. Ren, New fossil palaeontinids (Hemiptera, Cicadomorpha, Palaeontinidae) from the Middle Jurassic of Daohugou, China. *Alcheringa* **37**, 19–30 (2013).

35. Y. Fu, C. Cai, D. Y. Huang, A new palaeontinid (Insecta, Hemiptera, Cicadomorpha) from the Upper Jurassic Tiaojishan Formation of northeastern China and its biogeographic significance. *J. Paleo.* **94**, 513–520 (2020).

36. B. Wang, H. C. Zhang, T. Wappler, J. Rust, Palaeontinidae (Insecta: Hemiptera) from the Upper Jurassic Solnhofen Limestone of Germany and their phylogenetic significance. *Geol. Mag.* **147**, 570–580 (2010).

37. C. P. Ellington, Power and efficiency of insect flight muscle. *J. Exp. Biol.* **115**, 293–304 (1985).

38. B. Wang, "Mesozoic Hemiptera and Coleoptera (Insecta) from northeastern China: Taxonomy, evolution, and taphonomy," thesis, Nanjing Institute of Geology and Palaeontology, Chinese Academy of Sciences, Nanjing, China (2009).

39. R. J. Wootton, Support and deformability in insect wings. *J. Zool.* **193**, 447–468 (1981).

40. H. Wan, H. Dong, K. Gai, Computational investigation of cicada aerodynamics in forward flight. *J. R. Soc. Interface* **12**, 20141116 (2015).

41. S. Krishna, M. Cho, H. N. Wehmann, T. Engels, F. O. Lehmann, Wing design in flies: Properties and aerodynamic function. *Insects* **11**, 466 (2020).

42. P. Shi, Y. Jiao, K. J. Niklas, Y. Li, X. Guo, K. Yu, L. Chen, L. E. Hurd, Sexual dimorphism in body size and wing loading for three cicada species. *Ann. Entomol. Soc. Am.* **115**, 344–351 (2022).

43. C. P. Ellington, The aerodynamics of hovering insect flight II. Morphological parameters. *Philos. Trans. R. Soc. B.* **305**, 17–40 (1984).

44. S. Ho, H. Nassef, N. Pornsinsirirak, Y. C. Tai, C. M. Ho, Unsteady aerodynamics and flow control for flapping wing flyers. *Prog. Aeronaut. Sci.* **39**, 635–681 (2003).

45. C. R. Betts, R. J. Wootton, Wing shape and flight behaviour in butterflies (Lepidoptera: Papilionoidea and Hesperioidea): A preliminary analysis. *J. Exp. Biol.* **138**, 271–288 (1988).

46. R. Dudley, Biomechanics of flight in Neotropical butterflies: Morphometries and kinematics. *J. Exp. Biol.* **150**, 37–53 (1990).

47. R. Dudley, R. B. Srygley, Flight physiology of Neotropical butterflies: Allometry of airspeeds during natural free flight. *J. Exp. Biol.* **191**, 125–139 (1994).

48. K. Berwaerts, H. Van Dyck, P. Aerts, Does flight morphology relate to flight performance? An experimental test with the butterfly *Pararge aegeria*. *Funct. Ecol.* **16**, 484–491 (2002).

49. A. Ortega Ancel, R. Eastwood, D. Vogt, C. Ithier, M. Smith, R. Wood, M. Kovač, Aerodynamic evaluation of wing shape and wing orientation in four butterfly species using numerical simulations and a low-speed wind tunnel, and its implications for the design of flying micro-robots. *Interface Focus* **7**, 0160087 (2017).

50. H. Li, M. R. A. Nabawy, Wing planform effect on the aerodynamics of insect wings. *Insects* **13**, 459 (2022).

51. U. T. A. Oberdörster, P. R. Grant, Predator foolhardiness and morphological evolution in 17-year cicadas (*Magicicada* spp.). *Biol. J. Linn. Soc.* **90**, 1–13 (2007).

52. K. Schmidt-Nielsen, Locomotion: Energy cost of swimming, flying, and running. *Science* **177**, 222–228 (1972).

53. R. D. Stevenson, K. Corbo, L. B. Baca, Q. D. Le, Cage size and flight speed of the tobacco hawkmoth *Manduca sexta*. *J. Exp. Biol.* **198**, 1665–1672 (1995).

54. F. Song, K. L. Lee, A. K. Soh, F. Zhu, Y. L. Bai, Experimental studies of the material properties of the forewing of cicada (Homoptera, Cicadidae). *J. Exp. Biol.* **207**, 3035–3042 (2004).

55. C. P. Ellington, C. Van Den Berg, A. P. Willmott, A. L. Thomas, Leading-edge vortices in insect flight. *Nature* **384**, 626–630 (1996).

56. M. H. Dickinson, F. O. Lehmann, S. P. Sane, Wing rotation and the aerodynamic basis of insect flight. *Science* **284**, 1954–1960 (1999).

57. B. Wang, H. C. Zhang, Y. Fang, Y. T. Zhang, A revision of Palaeontinidae (Insecta: Hemiptera: Cicadomorpha) from the Jurassic of China with descriptions of new taxa and new combinations. *Geol. J.* **43**, 1–18 (2008).

58. S. Chatterjee, *The Rise of Birds: 225 Million Years of Evolution* (Johns Hopkins Univ. Press, 2015).

59. R. B. Benson, R. A. Frigot, A. Goswami, B. Andres, R. J. Butler, Competition and constraint drove Cope's rule in the evolution of giant flying reptiles. *Nat. Commun.* **5**, 3567 (2014).

60. J. Bestwick, D. M. Unwin, R. J. Butler, M. A. Purnell, Dietary diversity and evolution of the earliest flying vertebrates revealed by dental microwear texture analysis. *Nat. Commun.* **11**, 5293 (2020).

61. Y. Yu, C. Zhang, X. Xu, Complex macroevolution of pterosaurs. *Curr. Biol.* **33**, 770–779.e4 (2023).

62. J. P. Tennant, P. D. Mannion, P. Upchurch, M. D. Sutton, G. D. Price, Biotic and environmental dynamics through the Late Jurassic–Early Cretaceous transition: Evidence for protracted faunal and ecological turnover. *Biol. Rev. Camb. Philos. Soc.* **92**, 776–814 (2017).

63. E. Romero-Lebrón, J. M. Robledo, X. Delclòs, J. F. Petrulevičius, R. M. Gleiser, Endophytic insect oviposition traces in deep time. *Palaeogeogr. Palaeoclimatol. Palaeoecol.* **590**, 110855 (2022).

64. A. P. Rasnitsyn, D. L. J. Quicke, *History of Insects* (Kluwer Academic Publisher, 2002).

65. C. Jouault, H. Tischlinger, M. Henrotay, A. Nel, Wing coloration patterns in the Early Jurassic dragonflies as potential indicator of increasing predation pressure from insectivorous reptiles. *Palaeoentomology* **5**, 305–318 (2022).

66. M. Kohli, H. Letsch, C. Greve, O. Béthoux, I. Deregnaucourt, S. Liu, X. Zhou, A. Donath, C. Mayer, L. Podsiadlowski, S. Gunkel, R. Machida, O. Niehuis, J. Rust, T. Wappler, X. Yu, B. Misof, J. Ware, Evolutionary history and divergence times of Odonata (dragonflies and damselflies) revealed through transcriptomics. *Iscience* **24**, 103324 (2021).

67. D. L. Swofford, *PAUP: Phylogenetic Analysis Using Parsimony*, *version 4.0b10* (Sinauer Associates, 2002).

68. F. Ronquist, M. Teslenko, P. van der Mark, D. L. Ayres, A. Darling, S. Hohna, B. Larget, L. Liu, M. A. Suchard, J. P. Huelsenbeck, MrBayes 3.2: Efficient Bayesian phylogenetic inference and model choice across a large model space. *Syst. Biol.* **61**, 539–542 (2012).

69. S. J. Gould, The disparity of the Burgess Shale arthropod fauna and the limits of cladistic analysis: Why we must strive to quantify morphospace. *Paleobiology* **17**, 411–423 (1991).

70. M. A. Wills, D. E. G. Briggs, R. A. Fortey, Disparity as an evolutionary index: A comparison of Cambrian and Recent arthropods. *Paleobiology* **20**, 93–130 (1994).

71. T. Guillerme, M. N. Puttick, A. E. Marcy, V. Weisbecker, Shifting spaces: Which disparity or dissimilarity measurement best summarize occupancy in multidimensional spaces? *Ecol. Evol.* **10**, 7261–7275 (2020).

72. G. T. Lloyd, Estimating morphological diversity and tempo with discrete character-taxon matrices: Implementation, challenges, progress, and future directions. *Biol. J. Linn. Soc.* **118**, 131–151 (2016).

73. P. Dixon, Vegan, a package of R functions for community ecology. *J. Veg. Sci.* **14**, 927–930 (2003).

74. T. Guillerme, dispRity: A modular R package for measuring disparity. *Methods Ecol. Evol.* **9**, 1755–1763 (2018).

75. H. Wickham, *ggplot2: Elegant Graphics for Data Analysis* (Springer-Verlag, 2016).

76. L. J. Revell, phytools 2.0: An updated R ecosystem for phylogenetic comparative methods (and other things). *PeerJ* **12**, e16505 (2024).

77. V. Bonhomme, S. Picq, C. Gaucherel, J. Claude, Momocs: Outline analysis Using R. *J. Stat. Softw.* **56**, 1–24 (2014).

78. H. Iwata, Y. Ukai, SHAPE: A computer program package for quantitative evaluation of biological shapes based on elliptic Fourier descriptors. *J. Hered.* **93**, 384–385 (2002).

79. C. A. Schneider, W. S. Rasband, K. W. Eliceiri, NIH Image to ImageJ: 25 years of image analysis. *Nat. Methods* **9**, 671–675 (2012).

80. D. Outomuro, F. Johansson, Bird predation selects for wing shape and coloration in a damselfly. *J. Evol. Biol.* **28**, 791–799 (2015).

81. S. S. Bhat, J. Sheridan, K. Hourigan, M. C. Thompson, Aspect ratio studies on insect wings. *Phys. Fluids* **31**, 121301 (2019).

82. F. J. Rohlf, The Tps series of software. *Hystrix* **26**, 9–12 (2015).

83. R. M. West, Best practice in statistics: The use of log transformation. *Ann. Clin. Biochem.* **59**, 162–165 (2022).

84. A. F. Emeljanov, Phylogeny of Cicadina (Homoptera) on comparatively morphological data (in Russian). *Proc. Russ. Entomol. Sci.* **69**, 19–109 (1987).

85. C. E. Pulz, G. S. Carvalho, Morfologia do adulto de *Deois flexuosa* (Walker, 1851) (Insecta, Hemiptera, Cercopidae). *Biociências* **6**, 95–117 (1998).

86. K. G. A. Hamilton, Spittlebugs of new-world Aphrophorinae, part 1: Ptyelini (Hemiptera, Cercopoidea). *Zootaxa* **3497**, 41–59 (2012).

87. A. Nel, J. Prokop, P. Nel, P. Grandcolas, D. Y. Huang, P. Roques, E. Guilbert, O. Dostál, J. Szwedo, Traits and evolution of wing venation pattern in paraneopteran insects. *J. Morphol.* **273**, 480–506 (2012).

88. J. Chen, B. Wang, H. C. Zhang, X. Wang, X. Zheng, New fossil Procercopidae (Hemiptera: Cicadomorpha) from the Middle Jurassic of Daohugou, Inner Mongolia, China. *Eur. J. Entomol.* **112**, 373–380 (2015).

89. J. Chen, Y. Zheng, G. Wei, X. Wang, New data on Jurassic Sinoalidae from northeastern China (Insecta, Hemiptera). *J. Palaeontol.* **91**, 994–1000 (2017).

90. T. Schubnel, L. Desutter-Grandcolas, F. Legendre, J. Prokop, A. Mazurier, R. Garrouste, P. Grandcolas, A. Nel, To be or not to be: Postcubital vein in insects revealed by icrotomography. *Syst. Entomol.* **45**, 327–336 (2020).

91. T. Bourgoin, R. R. Wang, M. Asche, H. Hoch, A. Soulier-Perkins, A. Stroiński, S. Yap, J. Szwedo, From micropterism to hyperpterism: Recognition strategy and standardized homology-driven terminology of the forewing venation patterns in planthoppers (Hemiptera: Fulgoromorpha). *Zoomorphology* **134**, 63–77 (2015).

92. D. S. Kopylov, A. P. Rasnitsyn, D. S. Aristov, A. S. Bashkuev, N. V. Bazhenova, V. Y. Dmitriev, A. V. Gorochov, M. S. Ignatov, V. D. Ivanov, A. V. Khramov, A. A. Legalov, E. D. Lukashevich, Y. S. Mamontov, S. I. Melnitsky, B. Ogłaza, A. G. Ponomarenko, A. A. Prokin, O. V. Ryzhkova, A. S. Shmakov, N. D. Sinitshenkova, A. Y. Solodovnikov, O. D. Strelnikova, I. D. Sukacheva, A. V. Uliakhin, D. V. Vasilenko, P. Wegierek, E. V. Yan, M. Zmarzły, The Khasurty fossil insect Lagerstätte. *Palaeontol. J.* **54**, 1221–1394 (2021).
